# *Pseudomonas aeruginosa* clinical and environmental isolates constitute a single population with high phenotypic diversity

**DOI:** 10.1186/1471-2164-15-318

**Published:** 2014-04-28

**Authors:** María-Victoria Grosso-Becerra, Christian Santos-Medellín, Abigail González-Valdez, José-Luis Méndez, Gabriela Delgado, Rosario Morales-Espinosa, Luis Servín-González, Luis-David Alcaraz, Gloria Soberón-Chávez

**Affiliations:** 1 Departamento de Biología Molecular y Biotecnología, Instituto de Investigaciones Biomédicas, Universidad Nacional Autónoma de México, Ciudad Universitaria, 04510 México, DF, México; 2 Departamento de Microbiología y Parasitología, Facultad de Medicina, Universidad Nacional Autónoma de México, Ciudad Universitaria, 04510 México, DF, México; 3 Departamento de Ecología de la Biodiversidad, Instituto de Ecología, Universidad Nacional Autónoma de México, Ciudad Universitaria, 04510 México, DF, México

**Keywords:** *P. aeruginosa* genomics and proteomics, Core genome, Pangenomics, Phenotypic diversity

## Abstract

**Background:**

*Pseudomonas aeruginosa* is an opportunistic pathogen with a high incidence of hospital infections that represents a threat to immune compromised patients. Genomic studies have shown that, in contrast to other pathogenic bacteria, clinical and environmental isolates do not show particular genomic differences. In addition, genetic variability of all the *P. aeruginosa* strains whose genomes have been sequenced is extremely low. This low genomic variability might be explained if clinical strains constitute a subpopulation of this bacterial species present in environments that are close to human populations, which preferentially produce virulence associated traits.

**Results:**

In this work, we sequenced the genomes and performed phenotypic descriptions for four non-human *P. aeruginosa* isolates collected from a plant, the ocean, a water-spring, and from dolphin stomach. We show that the four strains are phenotypically diverse and that this is not reflected in genomic variability, since their genomes are almost identical. Furthermore, we performed a detailed comparative genomic analysis of the four strains studied in this work with the thirteen previously reported *P. aeruginosa* genomes by means of describing their core and pan-genomes.

**Conclusions:**

Contrary to what has been described for other bacteria we have found that the *P. aeruginosa* core genome is constituted by a high proportion of genes and that its pan-genome is thus relatively small. Considering the high degree of genomic conservation between isolates of *P. aeruginosa* from diverse environments, including human tissues, some implications for the treatment of infections are discussed. This work also represents a methodological contribution for the genomic study of *P. aeruginosa*, since we provide a database of the comparison of all the proteins encoded by the seventeen strains analyzed.

## Background

*Pseudomonas aeruginosa* is an environmental, ubiquitous γ-proteobacterium that is also an important opportunistic human pathogen [1]. In contrast to other bacterial pathogens, *P. aeruginosa* genomes of clinical and environmental isolates are highly conserved, and all isolates are able to produce virulence-associated traits and are thus potential pathogens [2,3].

The pathogenicity of this bacterium depends on the production and secretion of multiple virulence factors that are regulated at the transcriptional level by the so-called quorum sensing (QS) response [4]. This genetic response is mediated by the bacterial production of acyl-homoserine lactones (autoinducers) that act as signal molecules interacting with transcriptional regulators of the LuxR family. *Pseudomonas aeruginosa* QS is a hierarchical regulatory cascade: LasR interacts with 3-oxo-dodecanoyl-homoserine lactone (3O-C_12_-HSL), produced by the LasI enzyme, and activates the transcription of *lasI*, and of several genes coding for virulence factors. It also activates transcription of *rhlR,* which encodes the second QS transcriptional regulator, and of *rhlI,* which encodes the enzyme that produces butanoyl-homoserine lactone (C_4_-HSL), which is the autoinducer that interacts with RhlR [4]. RhlR/C_4_-HSL in turn promotes the expression of genes responsible for the production of several virulence factors. These include among others, pyocyanin, lectin PA-IL (encoded by *lecA)*, and biosurfactant rhamnolipids (synthesized by the products of the *rhlAB* operon).

This bacterium represents an important public health problem, as one of the leading causes of nosocomial infections [5] and as an infectious agent for cystic fibrosis patients [6]. Additionally, the high level of antibiotic resistance shown by this bacterium [7] makes it very difficult to treat *P. aeruginosa* infections.

The first *P. aeruginosa* genome to be completely sequenced was that of the type strain PAO1 [8], which was described in 1955, as an isolate from the wound of a patient in Australia [9]. At present, the genome sequences of twelve clinical isolates and of a strain isolated from watermelon rhizosphere (M18) have been reported [NCBI data base, [8,10-18]]. Strain M18 produces higher levels of phenazine-carboxylic acid (PCA), the precursor of pyocyanin, at 30°C than at 37°C [19], and the hierarchy of the autoinducer-based transcriptional regulation of virulence associated traits, *i.e.* the QS response, is different to that of the type strain PAO1 [20]. However, the genomes of clinical and environmental isolates, including strain M18 are very highly conserved [21]. This high sequence identity at the genome level is not observed in other bacterial species, such as *Escherichia coli*[22], or even in other *Pseudomonas* species [23]. Furthermore, it has been reported that the sequenced genomes of *P. aeruginosa* strains, including PAO1, show more than 95% sequence identity in the genes of their core genomes. The only sequenced genome that shows a slightly lower sequence identity is that of the multiresistant strain PA7 isolated in Argentina, which belongs to a different clade [16].

It is difficult to explain the high degree of genomic conservation between *P. aeruginosa* clinical and environmental isolates, and one possible explanation is that all genomes sequenced to date belong to a subpopulation of this bacterial species, and thus do not represent the real genomic diversity of the species. In this respect, the existence of environmental strains with higher genomic variability, similar to that presented by other bacteria, would show that clinical and human related environmental isolates constitute a subpopulation that does not represent the real amount of genomic diversity within this bacterial species. We tested this genomic variability hypothesis by carrying out the genomic characterization of three *P. aeruginosa* strains isolated from non-human related environments, and one strain isolated from dolphin stomach, and compared them with the genomes of twelve clinical isolates of *P. aeruginosa* and strain M18 (Table 1).

**Table 1:** **General features of the *P. aeruginosa *** **strains used in this work**

**Strain**	Accession Number*	**Genome Size (Mb)**	**CDS**	**GC%**	**Environment of isolation**	**Reference**
148	ATAJ00000000	6.7	6,194	65.8	Dolphin, gastric juice	This work
ID4365	ATAI00000000	6.8	6,318	65.7	Indian Ocean	24
IGB83	ATAH00000000	6.5	6,106	66.1	Rotten coconut	26
M10	ATAG00000000	6.1	5,642	65.8	Cuatro Ciénegas,Coahuila	This work
2192*	AAKW00000000	6.9	5,915	66.2	Cystic fibrosis patient	10
39016*	AEEX00000000	6.86	6,409	65.55	Cornea from a patient with ulcerative keratitis	11
B136	NCBI-1280938	6.4	5,828	66.4	Isolate from an infant with community acquired diarrhea	NCBI
C3719*	AAKV00000000	6.2	5,221	66.5	Cystic fibrosis patient	10
DK2	NC_018080	6.4	5,960	66.3	Cystic fibrosis patient	12
LESB58	NC_011770	6.6	6,061	66.3	Epidemic strain from Manchester England	13
M18	NC_017548	6.33	5,770	66.5	Sweet melon rhizosphere	14
NCGM2.S1	NC_017549	6.76	6,358	66.1	Urinary tract infection patient	15
PACS2*	AAQW00000000	6.49	5,676	66.3	Cystic fibrosis patient, nosocomial infection	NCBI
PAO1	NC_002516	6.26	5,682	66.6	Burn wound isolate	9
PA7	NC_009656	6.59	6,369	66.4	Non-respiratory human isolate	16
UCBPP-PA14	NC_008463	6.54	5,977	66.3	Human burn patient	17
RP73	NCBI-1340851	6.34	5,762	66.5	Persistent isolate from a Cystic Fibrosis patient	18

*Corresponds to a genome draft.

The characterized environmental isolates are: strain ID4365, a marine isolate from the Indian Ocean which produces very high levels of pyoverdins and phenazines [24]; strain M10, that was isolated from the water spring of the Churince system at Cuatro Cienegas, Coahuila, Mexico (a system of ponds in the middle of the Chihuahuan Desert with features that resemble those of an ancient ocean [25]); and strain IGB83, which is a highly lipolytic strain isolated in the rainforest of the Mexican State of Chiapas from a rotten coconut [26]. We also studied strain 148 (Table 1), since it was isolated from a dolphin kept in captivity in a marine aquarium in Cancún, Quintana Roo, Mexico and it enabled us to determine whether pathogenic interactions with mammals were possibly being selected in the *P. aeruginosa* genomic content, and therefore, whether strain 148 was probably more closely related to clinical strains than to those isolated from the ocean water column or other wild environments.

The genomic similarity of strain M18 to clinical *P. aeruginosa* strains (http://www.pseudomonas.com) suggests that clinical isolates are not a subpopulation of *P. aeruginosa,* which is the main question of our research, but it is still possible that strain M18 is a particular case that does not reflect the general situation of environmental *P. aeruginosa* strains.

In this work we show that the three environmental strains studied, and the dolphin-associated strain, are all pathogenic and phenotypically diverse with respect to production of virulence factors and motility. However, this phenotypic diversity is not reflected in genomic variability, since their genomes have a very high degree of similarity. Furthermore, detailed comparison of all the orthologous proteins encoded in the genomes of the seventeen *P. aeruginosa* strains studied in this work (Table 1), shows that the genomes of such a diverse group of *P. aeruginosa* strains (twelve isolates from human, two water inhabitants, two plant-associated strains and one strain isolated from a dolphin) are surprisingly similar, thus having a large proportion of their genome conserved between them (core genome). We also found that the genomes of the four isolates described in this study (Table 1) harbor sequences that have been reported to be part of genomic islands (GIs) that have been related to pathogenic phenotypes [18,27-29], suggesting that these strains share genetic information with clinical isolates. However the four studied strains do not show antibiotic resistance, suggesting that the high incidence of multi-resistant strains among clinical isolates is due to selection pressures for these traits caused by the use of antibiotics for the treatment of *P. aeruginosa* infections.

The database of all the protein families, their abundance and the known molecular functions encoded by the genomes of the seventeen *P. aeruginosa* strains analyzed in this work, is provided (Additional file 1: Table S1 http://figshare.com/articles/Pan_genome_Pseudomonas_aeruginosa_Supplementary_Table_S1/760583). This constitutes a contribution for researchers working with this opportunistic bacterium.

## Results and discussion

### Phenotypic characterization of three environmental and a dolphin-associated *P. aeruginosa* strains

We tested the resistance-pattern of strains ID4365, M10, IGB83 and 148 to 20 antibiotics (see Methods section) and found that all four were sensitive to all antibiotics tested. The multisensitive pattern of these strains is completely different to the patterns shown by a collection of 100 *P. aeruginosa* clinical isolates, which are predominantly multi-resistant [29]. The result obtained is expected since neither the environmental strains nor the dolphin-associated strain have been subjected to selection by the presence of antibiotics used in the treatment of *P. aeruginosa* infections.

We tested the virulence of the studied *P. aeruginosa* strains by intraperitoneal injection of mice. We found that the strains ID4365, M10, IGB82 and 148 were pathogenic to mice, but that they were only 60% to 80% as virulent as the clinical PAO1 strain (Table 2). If this group of strains constituted a subpopulation of *P. aeruginosa*, the virulence of the three environmental strains would not be expected, since virulence would then be a feature of clinical isolates and strains that live in environments close to humans. In this regard, we expected environmental strains to have a considerably reduced virulence, if at all, but the virulence pattern coincides with that of other strains previously described [19,30].

**Table 2:** Determination of strain virulence in the mouse model*

**STRAIN**	**% lethality (10**^**8**^**)**	**% lethality (10**^**9**^**)**	**% lethality (10**^**10**^**)**
PAO1	100	100	100
ID4365	60	80	100
M10	80	100	100
IGB83	60	100	100
148	60	80	100

*The number between parentheses corresponds to the number of colony forming units that were injected intraperitoneally to each one of the five mice used in each assay.

To determine whether these four strains (ID4365, M10, IGB83 and 148) were able to kill mice by a similar mechanism as that reported for PAO1 and other *P. aeruginosa* clinical isolates, we measured their production of three QS-regulated virulence factors: rhamnolipids, pyocyanin and elastase (Table 3). The genes coding for synthesis of rhamnolipids and pyocyanin are regulated by RhlR/C_4_-HSL, whereas the gene for elastase is regulated by LasR/3O-C_12_-HSL [4]. The production of QS-regulated virulence factors was determined at 30°C, an environmental temperature, and at 37°C, the human body temperature.

**Table 3:** **Phenotypic characterization of *P. aeruginosa *** **strains analyzed compared to the type strain PAO1**^**a**^

**Strain**	**Rhls 30°C**	**Rhls 37°C**	**Pyo 30°C**	**Pyo 37°C**	**Elast 30°C**	**Elast 37°C**	**Swim**	**Swar**
PAO1	141.1 ± 15.7	178.2 ± 16.8	2.34 ± 0.46	5.29 ± 0.23	0.23 ± 0.06	0.34 ± 0.04	+	+
ID4365	170.2 ± 9.9	255 ± 11.1	197.2 ± 6	24.5 ± 1.5	ND*	ND*	-	-
M10	234.5 ± 17.8	152.8 ± 5.3	1.6 ± 0.6	0.36 ± 0.1	0.41 ± 0.04	0.53 ± 0.11	+	++
IGB83	297.6 ± 5	257.6 ± 31.9	ND*	ND*	0.32 ± 0.02	0.43 ± 0.03	+	++
148	87.9 ± 10.4	158.6 ± 5.2	14.05 ± 0.44	8.95 ± 0.28	ND*	ND*	-	-

^a^Rhamnolipids (Rhls), pyocyanin (Pyo) and elastase (Elast) production was determined after 24 hours of growth at 30°C and 37°C. ND* means not detected. Swimming (Swim) and swarming (Swar) phenotypes were determined as described in Materials and Method section. Examples of motility phenotypes are shown in the supplementary materials.

In contrast to what has been reported for the clinical strains PAO1 and PA14 strains (Grosso-Becerra MV, Croda-García G, Merino E, Servín-González L, Mojica-Espinosa R, Soberón-Chávez G: **Regulation of *Pseudomonas aeruginosa *virulence factors by two novel RNA thermometers,** submitted, [31]), we found that the Indian Ocean isolate, strain ID4365 produces high levels of pyocyanin at 37°C [24] and even higher levels at 30°C (Table 3). This pattern of thermoregulation is similar to that reported for strain M18 [19], but the marine strain ID4365 lacks the sequence present in the 3’ region of the operon *phzA1B1C1D1E1F1G1* that has been implicated in thermoregulation in strain M18 [19].

**Figure 1:**
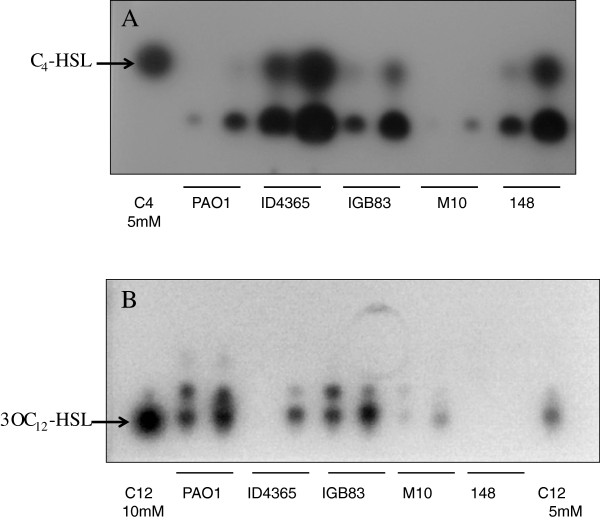
**Detection of autoinducers butanoyl-homoserine lactone (C**_**4**_**-HSL) and 3-oxo-dodecanoyl homoserine lactone (3O-C**_**12**_**-HSL) by thin layer chromatography (TLC) when different *P. aeruginosa *strains were grown at 30°C and 37°C.** The first lane of each strain corresponds to cultures grown at 30°C and the second to cultures grown at 37°C. AHLs extraction was performed as described in Experimental procedures. In **(A)** the plate was overlaid after chromatography with the short-chain AHL biosensor *C. violaceum* CV026 and in **(B)** the silica TLC plate was run in methanol 60% for 4 h and overlaid after chromatography with the long-chain AHL biosensor *E. coli* (pSB1075).

**Figure 2:**
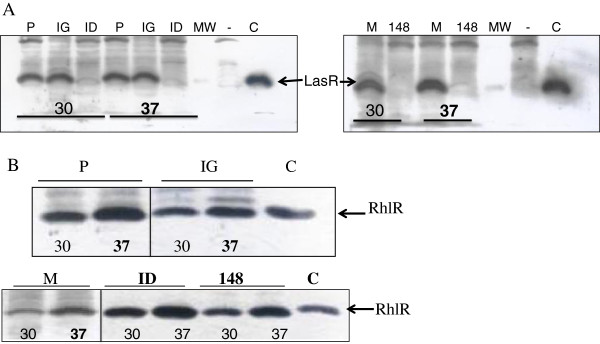
**Quantification by Western blot of the concentration of LasR (A) and RhlR (B) when different *P. aeruginosa *strains were cultivated at 30°C and 37°C.** Strains analyzed are: PAO1 (P), IGB83 (IG), M10 (M), ID4365 (ID) and 148. In panel A PAOR1 (Δ*lasR::Tc*) was used as negative control and is denoted by -. C: corresponds to the expression of LasR from pECP64 (A) and RhlR from pECP61.5 (B). MW refers to protein molecular weight standards. Lanes in B are divides by vertical lines to denote that the picture that is shown has been reconstructed because those lanes corresponding to PAO1 and IGB83 were not contiguous, and in the case of M10, the picture showing both lanes were exposed for a longer period than that where samples of strains ID4365 and 148 are shown.

Even though strain ID4365 is quite distinct to PAO1 with respect to the thermoregulation of pyocyanin production, it is very similar regarding production of rhamnolipids, C_4_-HSL and RhlR concentration at the two tested temperatures, since all these traits are expressed at higher levels at 37°C (Table 3; Figures 1 and 2). However C_4_-HSL levels produced by this strain are much higher than those produced by either PAO1 or the other three *P. aeruginosa* strains studied in this work (Figure 1). Another characteristic of strain ID4365 is its low production of 3O-C_12_-HSL, LasR and elastase activity (Table 3; Figures 1 and 2). This strain is unable to swim or swarm (Table 3; Additional file 2: Figure S1).

Strain M10, the water-spring isolate, produces similar levels of rhamnolipids, pyocyanin, elastase (Table 3), 3O-C_12_-HSL autoinducer (Figure 1) and LasR (Figure 2) to those produced by PAO1, but its production of rhamnolipids and pyocyanin is higher at 30°C than at 37°C (Table 3). Another particular feature of this strain is the low concentration of RhlR and C_4_-HSL produced at both temperatures tested (Figures 1 and 2).

*Pseudomonas aeruginosa* has been found to establish pathogenic interactions with different hosts including plants [32]. Strain IGB83 can be considered not only an environmental strain, but also a potential plant pathogen, since it was isolated from a rotten fruit. We found that this strain produces very low levels of pyocyanin at either temperature tested (Table 3), and that rhamnolipids production is not thermoregulated, as it is in strains PAO1, ID4365 and 148 (Table 3).

Strain 148, isolated from a dolphin stomach shows the same pattern of thermoregulation of rhamnolipids, C_4_-HSL and RhlR production as PAO1 type strain (Table 3; Figures 1 and 2), but its pyocyanin production is higher at 30°C than at 37°C (Table 3). This strain completely lacks elastase activity and production of 3O-C_12_-HSL. We were unable to detect the presence of LasR (Table 3; Figures 1 and 2).

Contrary to what has been found for two *P. aeruginosa* clinical isolates, PAO1 and PA14 (Grosso-Becerra MV, Croda-García G, Merino E, Servín-González L, Mojica-Espinosa R, Soberón-Chávez G: **Regulation of *Pseudomonas aeruginosa *virulence factors by two novel RNA thermometers,** submitted, [31]), three of the strains studied in this work (ID4365, M10 and 148), synthesize higher levels of pyocyanin at an environmental temperature than at the human body temperature (Table 3).

Strain 148 is unable to swim or swarm (Table 3; Additional file 2: Figure S1). The non-motility of the two strains isolated from marine-related habitats (ID4375 and 148) suggests that this might be a common phenotype among *P. aeruginosa* living in the sea. The non-motile phenotype of strain 148 is due to multiple mutations in *fliC* and *fliD* genes (Additional file 3: Figure S2). However the genomic basis of the strain ID4365 inability to swim or swarm was not detected, since there are no-apparent mutations in the different genes involved in swimming or swarming that were analyzed (Adittional file 3: Figure S2).

### Analysis of QS-dependent genes

Detailed analysis of the sequence of QS-dependent genes such as *rhlA, rhlB, rhlR, rhlI, lecA, lasB,* and *lasI* showed that they are identical in the four *P. aeruginosa* strains studied (ID4365, M10, IGB83, and 148), and that is also the case for *lasR* in the three environmental isolates. Genome assembly of these four strains does not enable the full-length sequence annotation of the duplicated *phzA1B1C1D1E1F1G1* and *phzA2B2C2D2E2F2G2* operons since they are nearly identical and all of the sequences collapse into a single contig. This is a well-known problem for duplicated sequences in genome assembly. In order to determine whether both operons are present in the four strains, we amplified the 5’ regions of both operons using PAO1 DNA as a control. We found that both *phz* operons are present in strains 148, ID4365, IGB83 and M10 (Additional file 2: Figure S3).

**Figure 3:**
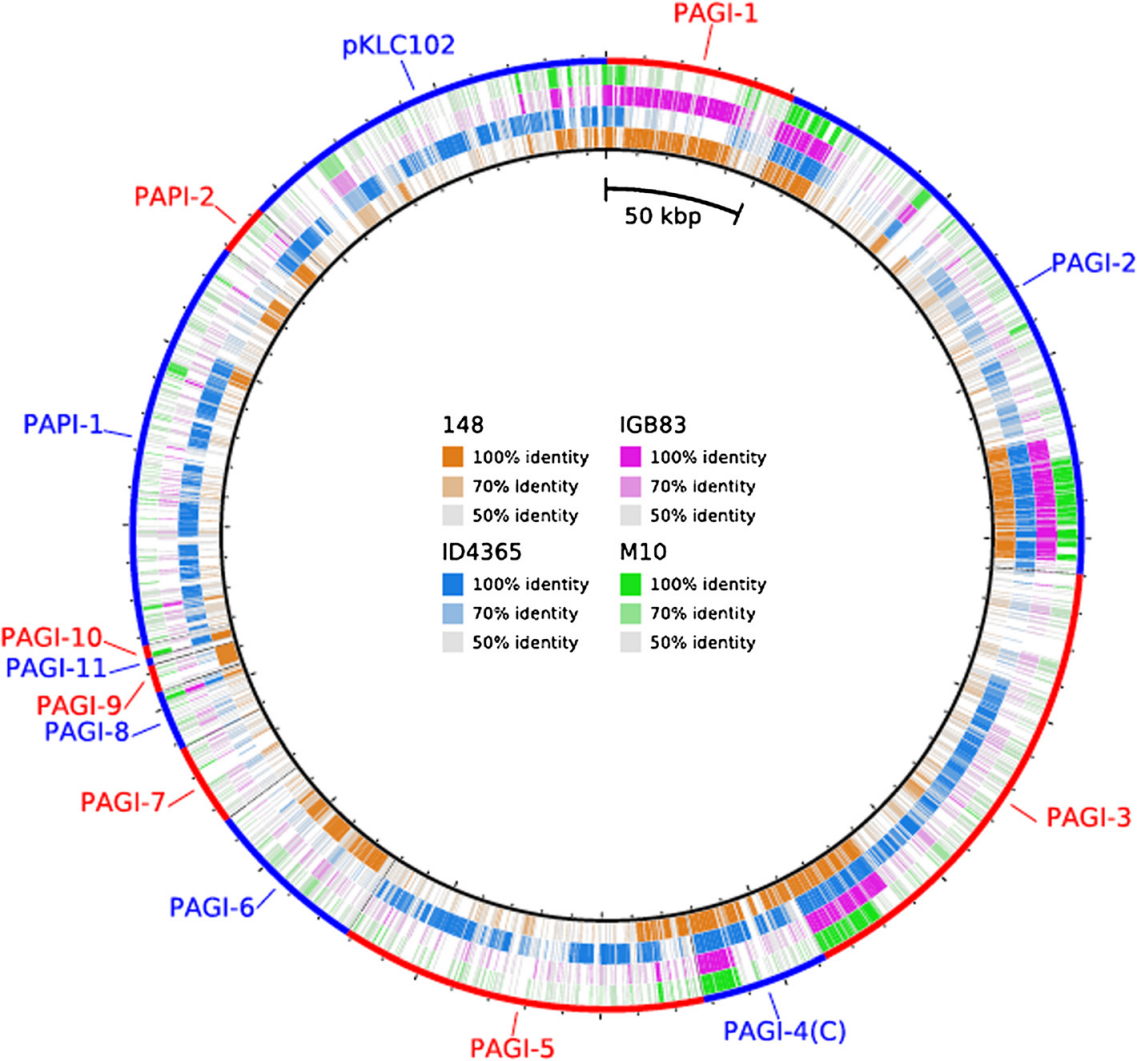
**Presence and variation of GEIs in the genome of strains ID4363, M10, IGB83 and 148.** The rings display tblastn comparisons of the anotated genes in strains 148, ID4365, IGB83 and M10 against the nucleotide sequences of different GIs. Each of the alternating red and blue segments in the outer ring represents the length of a particular island. The image is scaled to the nucleotide length of the genes: long tick marks on the external and internal circumference of the ring indicate increments of 50 kbp, while short tick marks indicate 10 kbp.

Interestingly, we found that the dolphin-associated strain 148 contains a 19,995 base pair deletion, which eliminated a region of the chromosome that corresponds to nucleotide 1,539,243 to nucleotide 1,559,239 of the PAO1 chromosome sequence. One end of this deletion is situated inside the homolog of PA1415, and the other end inside the intergenic region between *lasR* and *lasI*. This chromosomal deletion in strain 148 eliminates the *lasR* gene and the *lasI* promoter, thus explaining the lack of expression in this strain of LasR, 3O-C_12_-HSL and elastase. This chromosomal deletion in strain 148 was confirmed by PCR (Additional file 2: Figure S4). Thus the only apparent difference in the QS-related genes of the *P. aeruginosa* strains described in this study is this chromosomal deletion of nearly 20 kb in the dolphin-associated strain 148, that eliminates *lasR* and the *lasI* promoter region (Figure 3). The isolation of this strain, which is pathogenic, but lacks the QS-regulator LasR and its cognate autoinducer 3O-C_12_-HSL, constitutes a challenge to the current model for explaining the mechanism of *P. aeruginosa* virulence, and to the rationale for searching compounds that inhibit LasR activity as a means to treat infections caused by this bacterium [33].

The comparisons, using RNA-Seq, of transcriptomes from three environmental *P. aeruginosa* strains showed that the expression of quorum-sensing related genes is widely different, but that the master quorum-sensing regulators in all of them are well conserved [34]. Recently it was reported that two *P. aeruginosa* isolates from cystic fibrosis patients showed a wide phenotypic variability, even though both strains have a highly conserved genome sequence [35]. These observations are completely in accordance with the results presented in this work.

We have been unable to detect the genomic characteristics responsible for the low level of expression of the Las regulon in strain ID4365, nor the very high levels of pyocyanin that it produces, particularly at 30°C (Table 3; Figures 1 and 2). The genomic basis responsible for particular phenotypic characteristics of the four studied strains, which are different to those of clinical isolates, are also not clear.

The high genomic conservation of *P. aeruginosa* strains, however, is not reflected in a homogeneous pattern of QS-regulated virulence-traits expression. In this work we detected one strain that does not produce pyocyanin (IGB83, Table 3), two other strains that do not produce elastase (ID4365 and 148), and one strain (148) that does not produce the autoinducer 3O-C_12_-HSL, nor the LasR protein (Table 3; Figures 1 and 2). We detected that, contrary to the case of PAO1 and PA14 strains [31,32], higher levels of pyocyanin are produced at 30°C rather than at 37°C by the three studied strains that synthesize this phenazine, even though RhlR and the autoinducer C_4_-HSL tend to be produced at higher levels at 37°C by all the four strains studied. Despite these differences, all strains are virulent on the mice model, but show a slightly lower virulence than PAO1 strain (Table 2). It is our interest to determine the molecular mechanisms that underlie the different patterns of QS-regulated virulence trait expression on strains 148, ID4365, IGB83 and M10.

### Presence of genomic islands in strains 148, ID4365, IGB83 and M10

All sequenced *P. aeruginosa* strains sequenced to date show DNA sequences inserted in the core genome that are strain-specific or shared only by a fraction of isolates, which appeared to be transferred horizontally between strains [18,27,28]. Among these insertions, called genomic islands (GIs), there are some that are implicated in pathogenic interactions, and are thus assumed to be a characteristic of clinical isolates. The resistance to multiple antibiotics is also assumed to be only present in clinical isolates.

It was recently reported that a collection of *P. aeruginosa* clinical isolates has a high incidence of GIs [29]. The best characterized *P. aeruginosa* GIs related to pathogenicity are PAGI-1 to PAGI-4, PAPI-1, PAPI-2 and pKLC102 [27], but it has not been determined whether these elements can also be found in environmental isolates. We therefore searched for the presence of GIs in the chromosome of strains 148, ID4365, IGB83 and M10, and found that all four contain at least one ORF that has been reported to be part of GIs (Figure 3). The marine strain ID4365 has the highest number of GIs-related genes (Figure 3). This result reinforces the observation that environmental and clinical *P. aeruginosa* isolates do not constitute different subpopulations, and that GIs sequences are transferred horizontally between strains present in environments that are not close to humans and clinical strains.

### *P. aeruginosa* core and pan-genome analysis

Global analysis of seventeen *P. aeruginosa* strains (twelve isolated from humans, two from water, two from plants and one from a dolphin, Table 1) was performed by best reciprocal blast hits (BBH) of all the proteins encoded in their genomes. Using this analysis we found that the *P. aeruginosa* core genome consists of 4,455 orthologous encoding genes present in all the seventeen strains analyzed. Considering that some of the genes in the core genome are part of paralogous gene families we determined a total of 74,731 core genes in all the seventeen *P. aeruginosa* strains analyzed.

**Figure 4:**
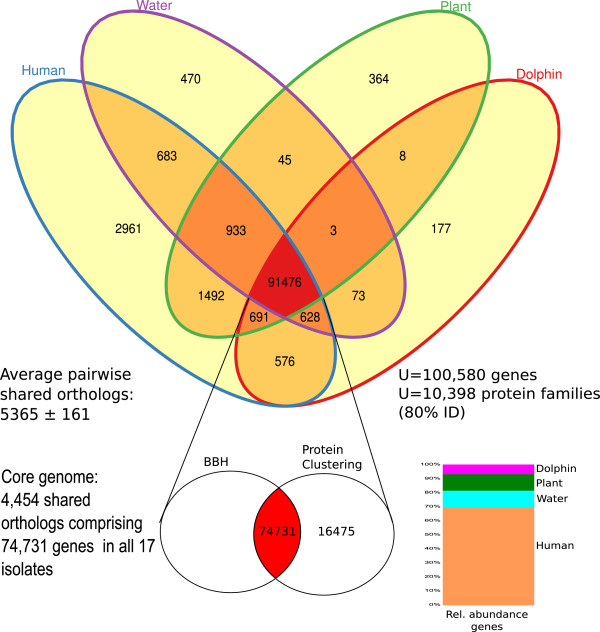
**Venn diagram showing the total number of predicted proteins shared by the distinct groups of *P. aeruginosa *strains.** A grand total of 100,580 predicted proteins were clustered into 10,398 families at 80% identity cut-off. The total overlap consists in 91,476 proteins that define the core genome *sensu latu*, protein families’ clusters, and proteins in at least 1 strain of all the 4 groups showed. The complete list of proteins, its annotation, the presence in each of the showed groups, and representative sequences are available at Additional file 1: Table S1 (http://figshare.com/articles/Pan_genome_Pseudomonas_aeruginosa_Supplementary_Table_S1/760583).

Another approach to determine the core genome is by means of clustering its predicted genes and quantifying genes present in all the strains analyzed and having ≥80% amino-acid identity. We were able to group 63,981 predicted genes into 5,484 protein families, which comprise the protein-clustering core. Both BBH and relaxed protein clustering (at least 1 gene present in each group) share a common set of 74,731 core genes clustered into 5,484 protein families. A Venn diagram displaying the shared genes between each one of the genomes and the environment from which the strains were isolated is shown in Figure 4.

This conserved genomic structure of *P. aeruginosa* is completely different to that reported for other bacterial species. A unique feature is that the core genome of this species constitutes more than 80% of its genetic repertoire and that its pan-genome is considerably smaller than the ones previously defined for other bacteria. For example the analysis of 61 sequenced *Escherichia coli* genomes showed that only 993 genes were present in all the genomes analyzed (core genome), and that this represented only 6% of the total number of gene families identified [36].

**Figure 5:**
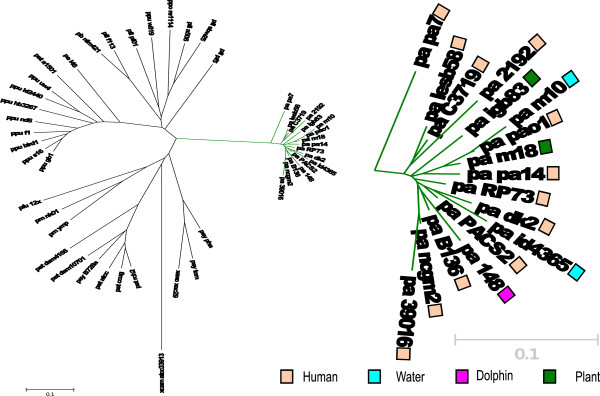
**Neighbor joining dendrogram showing the genomic similarity score (GSS) for selected genomes of the *Pseudomonas *genus, which depicts the overall similarity of the pairwise shared orthologous.** The average is to have 5366 ± 161 pair-wise shared orthologs within the *P. aeruginosa* group. The inset shows a close up of the *P. aeruginosa*'s branch. Abbreviations used: pa*, P. aeruginosa; pfl, P. fluorescens; ppo, P. poae; ppu, P. putida; pb, P. brassicacearum; pe, P. entomophila; pst, P. stutzeri; pm P. mendocina; pfu P. fulva; psy P. syringae; xaxo Xanthomonas axonopodis; xacam X. campestris*.

Using a genomic similarity score (GSS) [37] which depicts the overall similarity of the pairwise shared orthologs for selected genomes of the *Pseudomonas* genus, and the information of all the pairwise shared genes across all the isolates of *Pseudomonas* sp. completed genome sequences, we were able to build a dendrogram showing the overall genomic similarity (Figure 5). It is clear from the resulting GSS-tree (Figure 5) that all *P. aeruginosa* strains cluster in a single branch, which includes even strain PA7, which has been reported to be an outlier [16]. In the *P. aeruginosa* branch, strains life-styles and hosts are mixed (Figure 5), supporting the view that at the overall genomic level, human pathogenic strains are not a special structured subgroup within *P. aeruginosa*.

The analysis of the core genome and of the pan-genome by GSS provides the opportunity to get a broader view of the shared features across all the strains and to highlight the differences that can help us to understand particular niche adaptation strategies. The complete list of predicted proteins of the *P. aeruginosa* pan-genome and their distribution among strains is a valuable tool for the identification of genes that might code for traits that are important for niche adaptation (Additional file 1: Table S1). Particularly, this is a valuable resource to target human-related traits for diagnosis and direct therapy efforts. In this respect, the group of human-related *P. aeruginosa* strains is the one with the largest number of unique genes (Figure 4). The high number of unique genes in this group might be due to the over-representation of these strains among our sample (they constitute more than two thirds of the strains analyzed). Among the isolates from humans, there are several examples of genes that might be related to virulence, like those coding for a transcription regulator of the AraC family with high similarity to CdhR transcription regulators [38], fimbria related proteins, type IV secretion system, and adhesion related proteins, for example.

## Conclusion

In conclusion, the analysis of the genome sequence of three environmental and one dolphin-associated *P. aeruginosa* strains shows that their genomic content is extremely well conserved among them and with respect to previously sequenced strains (Figure 5). The seventeen strains analyzed (Table 1), which have representatives of isolates from diverse environments (human-, water-, plant- and dolphin-associated strains), grouped as a single clade with very low genetic diversity. The inset in Figure 5 shows that there is no such thing as a subgroup of the species that clusters apart based on the isolation environment. Strains isolated from humans distribute evenly and across the *P. aeruginosa* cluster and they can be more closely related to water, dolphin or plant isolated strains than to each other. Furthermore we found that the core genome defined as the number of proteins that are encoded in all the seventeen genomes sequences analyzed in detail represents a high proportion (more than 80%) of the total number of proteins encoded in them. These results rule out the possibility that clinical strains constitute a subpopulation of *P. aeruginosa* and that the genetic variability of this bacterial species is presented in environmental isolates. Our results reinforce previously reported results that suggest that genetic variability of *P. aeruginosa* is extremely low with high levels of recombination within the species [2,3,10,21,39].

The extremely high sequence similarity among different *P. aeruginosa* isolates is difficult to explain. One possibility to account for this high degree of genetic conservation between *P. aeruginosa* strains isolated from such diverse habitats would be that these bacteria have an inefficient mechanism of DNA exchange, but this does not seem to be the case as judged by the prevalence of GIs among clinical [29] and environmental isolates (Figure 3), and the population studies suggesting high levels of homologous recombination [39]. Other possibilities for explaining the low genetic variability of *P. aeruginosa* are that the population size of this bacterium is very low, or that a mechanism of gene conversion exists among different strains. These possibilities remain to be evaluated.

The high degree of sequence conservation of *P. aeruginosa* strains makes it a difficult task to device strategies for the treatment of infections caused by these bacteria that are based on vaccination or hygiene measures, and strengthens the need for designing strategies that inhibit the expression of virulence associated traits to contend with *P. aeruginosa* infections. Novel mechanisms of virulence inhibition, which take into account all the phenotypic diversity of this ubiquitous opportunistic pathogen, remain to be discovered.

## Methods

### Strains, culture conditions and microbiological procedures

*P. aeruginosa* strains (Table 1) were routinely propagated at 37°C on Luria-Bertani (LB) agar or LB broth [40] and all liquid cultures were grown with shaking (225 rpm). For experiments assessing the effect of temperature PPGAS media (phosphate-limited-peptone-glucose-ammonium) [41], was inoculated at a starting OD_600_ of 0.1, with overnight cultures in LB supplemented with the appropriate antibiotics and incubated at 30°C or at 37°C.

Swimming and swarming ability of P. aeruginosa strains was determined as described before [42,43].

### SDS gel electrophoresis and western blot analysis

For the preparation of crude cell extracts, PPGAS cultures were grown at 30°C and 37°C to an OD_600_ of 1.5. Cells were harvested and re-suspended in PA buffer (10 mM sodium phosphate buffer, 30 mM NaCl, 0.25% Tween-20, 10 mM EDTA, 10 mM β-mercaptoethanol, pH 7.5) prior to cell disruption through sonication. Cellular debris was removed by centrifugation (13,000 *g* for 15 min at 4°C) and the supernatant (containing soluble protein fraction) was mixed 1:1 v/v with Laemmli loading buffer [44]. Total protein concentration was determined by protein assay kit (Bio-Rad) with bovine serum albumin as standard. Equal amounts of proteins were separated by 12% sodium dodecyl sulfate-polyacrylamide gel electrophoresis (SDS-PAGE) and gels were electro-transferred onto a Hybond-C Extra nitrocellulose membrane (Amersham Biosciences). The membrane was blocked by 5% nonfat milk and incubated with a 1:1000 dilution of rabbit polyclonal antibody of either anti-RhlR or anti-LasR. Goat anti-rabbit immunoglobulin G (Santa Cruz Biotechnology) secondary antibody conjugated to horseradish peroxidase was used at a 1:10,000 of dilution. Detection was performed with a chemiluminescence-based system SuperSignal West Femto (Pierce) followed by exposure to an X-ray film (Amersham Biosciences) for autoradiography. PAO1 derived *rhlR* and *lasR* mutants were used as negative controls and as positive controls we use *Escherichia coli* strains harboring plasmids expressing *rhlR* (pECP61.5) and *lasR* (pECP64) [45].

### Virulence factors production

Pyocyanin was extracted from culture supernatants and measured as previously described [46]. The pyocyanin assay is based on the absorbance of pyocyanin at 520 nm in acidic solution. A 5 ml sample of culture supernatant was extracted with 3 ml of chloroform and then re-extracted into 1 ml of 0.2 N HCl to give a pink to deep red solution. The absorbance of this solution was measured at 520 nm. Concentrations, expressed as micrograms of pyocyanin produced per milliliter of culture supernatant, were determined by multiplying the optical density at 520 nm (OD_520_) by 17.072.

The concentration of rhamnolipids in the sample was estimated by the Orcinol method [47]. A 333 μl portion of the filtered supernatant was extracted twice with 1 ml of diethyl ether. The diethyl ether was evaporated to dryness and dissolved in 1 ml of deionized water. To 100 μl of each sample, 900 μl of a solution containing 0.19% orcinol (in 53% sulfuric acid) was added. The samples were heated at 80°C in a water-bath for 30 min and cooled for 15 min at room temperature and the A_421_ was measured. Concentrations of rhamnolipids were determined by comparing the data with those obtained with L-rhamnose standards between 0 and 50 μg/ml.

The elastolytic activity of LasB elastase was determinate using elastin Congo red as substrate and the procedure was modified from that described previously [48]. Briefly, the cells were grown in LB broth at 30° and 37°C, respectively for 24 h. Samples of the filter-sterilized supernatants were diluted 1:10 with LB and 1 mL was added 10 mg of Elastin Congo Red (Sigma) in glass tubes. The mixture was incubated at 37°C for 16 h with constant rotation (225 rpm), insoluble substrate was pelleted with centrifugation (1300 *g* for 10 min at 4°C) and absorbance of the supernatant was measured at 495 nm with a spectrophotometer using as blank elastin Congo red sample incubated with medium alone. The experiment was performed three times in triplicate with supernatants from three individual growth experiments. The values from the triplicate experiments were averaged and used as one value to represent each of the three experiments.

### Acyl-homoserine lactone (AHL) extraction and analytical thin-layer chromatography (TLC)

To evaluate profiles of AHLs, cells were grown in PPGAS media at 30°C and 37°C, respectively for 24 h. A 10-ml sample of culture supernatant was extracted twice with equal volumes of acidified ethyl acetate and then dried in a fume hood. The residues of extraction were then dissolved in 100 μl ethyl acetate and 5 μl were analyzed by thin layer chromatography (TLC). Analytical TLC was performed on reverse phase aluminium-backed RP18 F254S TLC plates (20 cm X 20 cm; Merck) [49]. Chromatograms were developed with methanol: water (60:40, v/v), then air-dried in a fume hood. The TLC plate was then overlaid with a thin film of agar seeded with the AHL reporter strain *C. violaceum* CV026 that produces the purple colour violacein in response to AHLs with N-acyl side chains between 4 and 8 carbons in length [50]. After incubation of the plate at 30°C for 24 h, AHLs were located as purple spots on a white background. Alternatively, TLC plates were overlaid with a thin layer of agar seeded with a culture of *E. coli* biosensor strain containing lux-based bioluminescence AHL reporter plasmid (pSB1075). This reporter contains the *P. aeruginosa lasR* gene and *lasI* promoter fused to *luxCDABE* from *P. luminescens* and detects 3-oxo-substituted AHL derivatives with acyl chain length from 4 to 12 carbons [51]. Light emission was detected using a system for chemiluminescence detection Gel Logic 112 (Kodak). All the experiments were performed at least twice.

### Determination of antibiotic resistance

To assess the susceptibility profiles to 20 antimicrobial agents of the strains of *Pseudomonas aeruginosa* the agar dilution method was used according to the guidelines established by the National Committee for Clinical Laboratory Standards (NCCLS), as previously described [29]. ATCC 27853 *Pseudomonas aeruginosa,* ATCC 25922 *Escherichia coli,* ATCC 35218 *Escherichia coli*, ATCC 29213 *Staphylococcus aureus,* and ATCC 29212 *Enterococcus faecalis* were used as controls in the susceptibility tests. All the strains were grown in Muller Hinton agar and harvested in sterile saline solution to achieve a turbidity equivalent to that of a No. 0.5 McFarland opacity standard. The antimicrobial agents tested against *P. aeruginosa* were: carbenicillin (16-64 μg/mL), ticarcillin (8-32 μg/mL), piperacillin (1-8 μg/mL) ticarcillin/clavulanic acid (8/2-32/2 μg/mL), piperacillin/tazobactam (1/4-8/4 μg/mL) ceftazidime (1-4 μg/mL) ceftriaxone (8-64 μg/mL) cefotaxime (8-32 μg/mL) cefepime (1-8 μg/mLl) imipenem (1-4 μg/mL) meropenem (0.25-1 μg/mlL) aztreonam (2-8 μg/mL) amikacin (1-4 μg/mL) gentamicin (0.5-2 μg/mL) tobramycin (0.25-1 μg/mL) polymyxin b (0.25-2 μg/mL) ciprofloxacin (0.25-1 μg/mL) norfloxacin (1-4 μg/mL) and levofloxacin (0.5-4 μg/mL). Agar dilution was performed using two-fold increments (across a range of 0.125 to 512 μg/mL) of each antimicrobial agent incorporated into Muller-Hinton agar. The concentration range for susceptibility and resistance are indicated in parenthesis with a MIC value lower than the cut-off to indicate susceptibility and two-fold dilutions above the cut-off to determine resistance. The criterion for intermediate susceptibility was based on isolates growing within one-fold dilution higher than the MIC value.

### Ethics statement

All mouse studies were conducted in accord with Guide for the Care and Use of Laboratory Animals (Committee for the Update of the Guide for the Care and Use of Laboratory Animals and Institute for Laboratory Animal Research, Washington D. C., 2011) and the Comité para el Cuidado y Uso de Animales de Laboratorio (CCUAL) and were approved by the Ethics committees of Instituto de Investigaciones Biomédicas - UNAM (approval No. ID201 09/02-2010).

### Virulence model

Bacteria to be injected into mice were cultured as described previously and quantitated by plate serial dilution method. For virulence studies, groups of five female BALB/cAnNHsd mice 6–8 weeks old (Harlan, Indianapolis, IN, United States) were inoculated intraperitoneally at day 0 with 100 μl of bacterial suspension at 1X10^7^, 1X10^8^, 1X10^9^ and 1X10^10^ colony forming units (CFU) of the each *P. aeruginosa* strain, respectively. Bacterial suspensions were prepared in sterile water for injection. Intraperitoneal injection without bacterial cells was used as a control group. Before and after experimental use, the animals were maintained in cages at 5 mice per cage with food and water available *ad libitum*. Mice from all treatment groups were monitored at 24 h after infection and died mice were incinerated.

### Genomes assembly and annotation

The genomes of isolates 148, ID4365, IGB83, and M10 were sequenced using the Illumina Genome Analyzer II. The libraries were prepared using a pair-ended protocol with an insertion average length of 500 bp. Quality control analysis and trimmi [52] and Celera assemblers [53]. The read average length goes to 35.99 bp. The estimated coverage goes from 39.49 to 73.48X fold. The draft genomes assemblies were done first by quality control check by using FastX (http://hannonlab.cshl.edu/fastx_toolkit), and then inputted into Velvet genome assembler [54]. Multiple assemblies were conducted to calibrate the optimum kmers parameters. We chose a global optimum of 23 kmers for *de novo* pair-ended assembly. The genomic alignments against the *P. aeruginosa* reference genomes were done with the MUMmeralignment suite [54] using the maxmatch option for nucleotide alignments. The MUMmer alignments were used to aid in the further assembly and scaffolding of the sequenced genomes, additional processing of the assembly was done with the Bambus program of the AMOS suite [55]. A second independent assembly and processing was done by the CLC Genomics Workbench version 5.5.1, and scaffolding and gap closure using SSPACE [56] and GapFiller version 1.10 [57]. Automated gene calling and annotation was conducted using the RAST Server [58].

The Whole Genome Shotgun (WGS) projects for *P. aerugionsa* isolates M10, IGB83, 148, ID4365 have been deposited at DDBJ/EMBL/Genbank under the project accessions ATAG00000000, ATAH00000000, ATAI00000000, ATAJ00000000, respectively. The versions described in this paper are the first versions.

### Genome Similarity Score

Genome Similarity Score (GSS) [38] was built with seven of the public released *Pseudomonas* strains. This is done by getting all the pairwise orthologs by means of reciprocal best blast hits (BBH) and getting individual bit-scores, comparing the sum of the comparison bit-scores and normalizing it against the self GSS bit-scores sum. This score has been successfully used before when comparing the overall genomic similarity of related species [59,60]. The range of GSS goes from 1 (identical strains) to 0 (non-related strains). The strength of this score relies on having genetic distances by means of comparing all the pairwise shared orthologs. The genetic distances are then sorted into a distance matrix and then plotted as a dendrogram. We also calculated the GSS distance with two out-groups strains of the genus *Xanthomonas.* To plot the GSS as dendrograms we got the inverse (1-GSS) distance value and then plot it using Mega (Neighbor-Joining) [61], and Figtree v 1.4.0 (http://tree.bio.ed.ac.uk/software/figtree/). The accessions for the genomes used in the GSS calculations are: NC_002516.2, NC_002947.3, NC_004129.6, NC_004578.1, NC_004632.1, NC_004633.1, NC_005244.2, NC_005773.3, NC_007005.1, NC_007274.1, NC_007275.1, NC_007492.2, NC_008027.1, NC_008463.1, NC_009434.1, NC_009439.1, NC_009512.1, NC_009656.1, NC_010322.1, NC_010501.1, NC_011770.1, NC_012660.1, NC_015379.1, NC_015410.1, NC_015556.1, NC_015733.1, NC_015740.1, NC_016830.1, NC_017530.1, NC_017532.1, NC_017548.1, NC_017549.1, NC_017911.1, NC_017986.1, NC_018028.1, NC_018080.1, NC_018177.1, NC_018220.1, NC_018746.1, NC_019670.1, NC_019905.1, NC_019906.1, NC_019936.1, NC_019937.1, NC_019938.1, NC_019939.1, NC_020209.1, NC_020829.1, NC_020912.1, and the out-groups NC_003902, NC_20800.1. The *P. aeruginosa* genomes 39016, 2192, B136, RP73, C3719, and PACS2 where downloaded in its last version from http://www.pseudomonas.org.

### GIs analysis

In order to assess the presence, absence and variation of certain GIs in the environmental strains’ genomes, we performed tblastn comparisons. As database, we used a Multi-FASTA file with the nucleotide sequences of different genomic islands from the PAGI and PAPI families, as well as from the integrative plasmid pKLC102; as query, we used the protein sequences from all the annotated genes in strains 148, ID4365, IGB83 and M10. A ring representation was then created with the BLAST Ring Image Generator [62]. The accessions for the GIs used in this analysis are: AF241171, AF440523, AF440524, AY258138, EF611301, EF611302, EF611303, EF611304, EF611305, EF611306, EF611307, NC_019202, AY273869, and AY273870.

### Core-genome analysis

The core genome *sensu stricto* was obtained by means of BBH, as described before [59], of all the available *P. aeruginosa* complete genomes available up-to date (May 2013). We define the core genome as the orthologs that are present in each and every one of the analyzed genomes. This is done by parsing the BBH results into tables and sorting them out by the presence/absence of a BBH match. The complete list of the isolates used in this study is shown in Table 1. The core genome *sensu latu* was done by protein clustering (80% identity) and present in at least 11 representatives in each cluster.

### Pan-genome analysis

The pan-genome was described by means of comparing and clustering all the predicted proteins of the eleven analyzed strains (Table 1). The clustering was done using CD-HIT version 4.6 [63], using a 0.80 identity threshold, with longest-sequence-first list removal algorithm. Results were parsed into a table (Additional file 1: Table S1) where a representative sequence was selected to represent every single protein family, each *P. aeruginosa* isolate has a column indicating the protein family representatives copy numbers within each of the analyzed genomes. Next, some basic calculations are shown displaying the total number of the members of each protein family. Each orthologous identified by the core genome analysis is shown in the column titled “Core Orthologous”. A second way to identify the core genome would be to make protein clustering and a cut-off of abundance, this is shown in the column named “Core.Prot.Fam”, using a threshold of 11 for the minimum abundance. The genomes where clustered based on its environmental isolation types defining clusters of Water (ID4365, M10), Plant (IGB83, M18), Dolphin (148), and human-associated isolates (NCGM2, LESB58, PAO1, PA14, DK2, PA7). Additional file 1: Table S1 shows the count of the predicted proteins that are exclusive to one kind of these environmental types (Water.only, Plant.only, Dolphin.only, Human.only). Finally the predicted protein sequence is available to perform further analysis, at the last field of the table.

### Statistical analyses

All the statistical analyses were done on R (ver 2.14.1) [64]. Using the gplots [65], and vennerable (https://r-forge.r-project.org/projects/vennerable/) packages.

### Availability of supporting data

The supporting data of our paper is now available. The reference is: Pan-genome Pseudomonas aeruginosa Additional file 1: Table S1. (http://figshare.com/articles/Pan_genome_Pseudomonas_aeruginosa_Supplementary_Table_S1/760583).

## Competing interests

The authors declare that they have no competing interests.

## Authors’ contributions

MVGB carried out the experiments shown in Table 3 and Figures 1 and 2; CSM participated in the bioinformatic analysis of specific genomic regions including those coding for motility and QS-related genes (and also for data shown in Additional file 1: Table S1) and Gis (he constructed Figure 3); AGV determined the virulence of strains in the mouse models and data shown in Additional file 2: Figure S1; JLM and GD did preliminary experiments for GIs detection by hybridization and contributed in the microbiological characterization of *P. aeruginosa* strains, including antibiotic resistance. RME participated in designing the research project specially the part related to the initial detection of GIs by hybridization and in antibiotic resistance determination; LSG was also involved in the design of the research and was involved in data analysis particularly the data related to pyocyanin production; LDA designed all bioinformatics strategies used from annotation of genomes to the analysis of *P. aeruginosa* pangenome (specifically the data shown on Figures 4 and 5 and Additional file 1: Table S1); GSC is the PI researcher who coordinated and designed most of the project, analyzed the data and wrote the paper.

## Supplementary Material

Additional file 1: Table S1 Constructed from biom file Water Plant Dolphin Human. Click here for file

Additional file 2: Figure S1 Swarming and swimming motility-phenotype of different *P. aeruginosa* strains. **Figure S3.** Identification of the presence of two reiterated *phz* (*phz1* and *phz2*) operons in different *P. aeruginosa* strains by PCR amplification. **Figure S4.** Corroboration of the 20 Kb deletion in the strain 148, which includes *lasR* and *lasI* promoter region. Click here for file

Additional file 3: Figure S2 Amino-acids sequence alignment of proteins encoded by different genes that participate on cell swimming and swarming, of strains 148, ID4365, IGB83, M10 and PAO1. Click here for file
