# Supplementary material for: Pseudomonas aeruginosa clinical and environmental isolates constitute a single population with high phenotypic diversity
Source: BMC Genomics. 2014 Apr 28;15:318. doi: 10.1186/1471-2164-15-318 (PMC4234422; doi:10.1186/1471-2164-15-318)
Supplement: Additional file 2: Figure S1 — Swarming and swimming motility-phenotype of different P. aeruginosa strains. Figure S3. Identification of the presence of two reiterated phz (phz1 and phz2) operons in different P. aeruginosa strains by PCR amplification. Figure S4. Corroboration of the 20 Kb deletion in the strain 148, which includes lasR and lasI promoter region. [file 1471-2164-15-318-S2.doc]

**Supplementary information**

Additional file 2.

Grosso-Becerra *et al. Pseudomonas aeruginosa* clinical and environmental isolates constitute a single population with high phenotypic diversity.

**Figure S1. Swarming and swimming motility phenotype of *P. aeruginosa* strains.**

**Figure S3. Identification of the presence of two reiterated *phz* (*phz1* and *phz2*) operons in different *P. aeruginosa* strains by PCR amplification.**

**Figure S4. Corroboration of the 20 Kb deletion in the strain 148, which includes *lasR.***

**A**


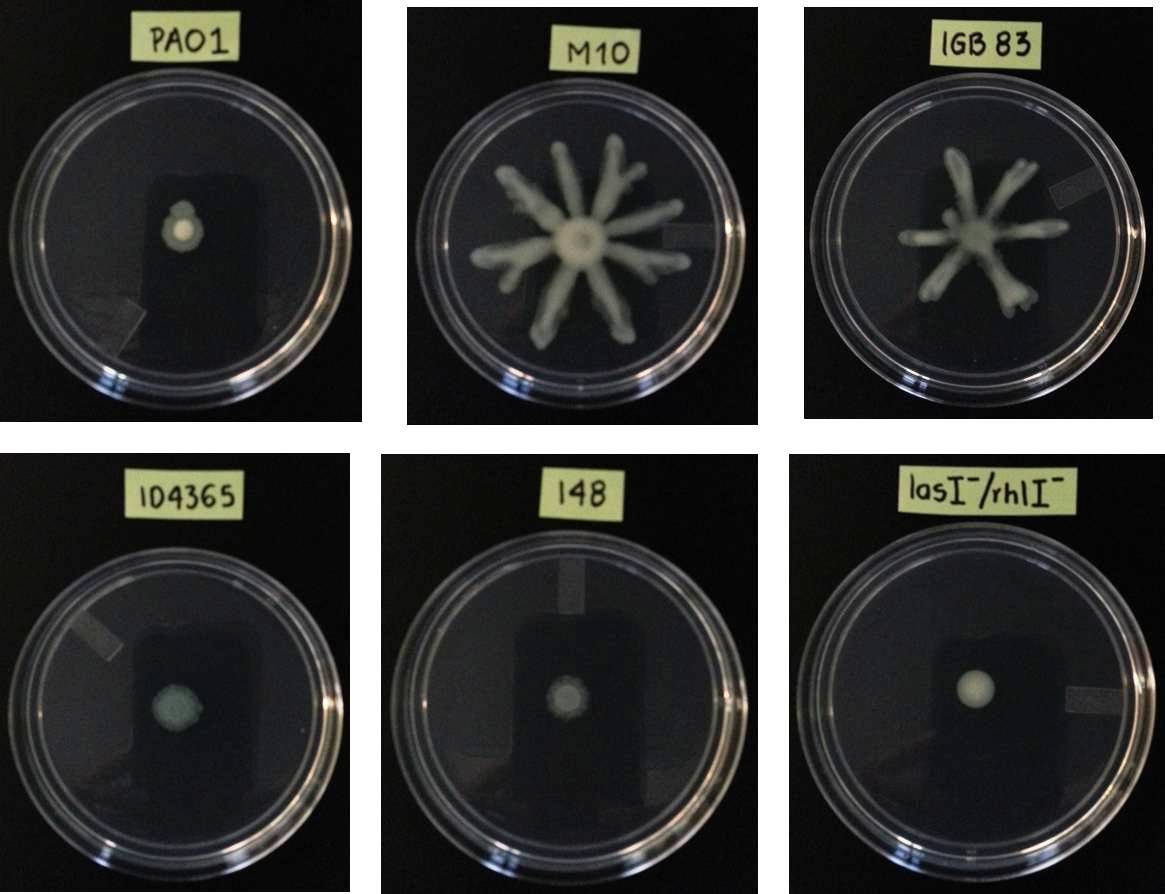


**B**


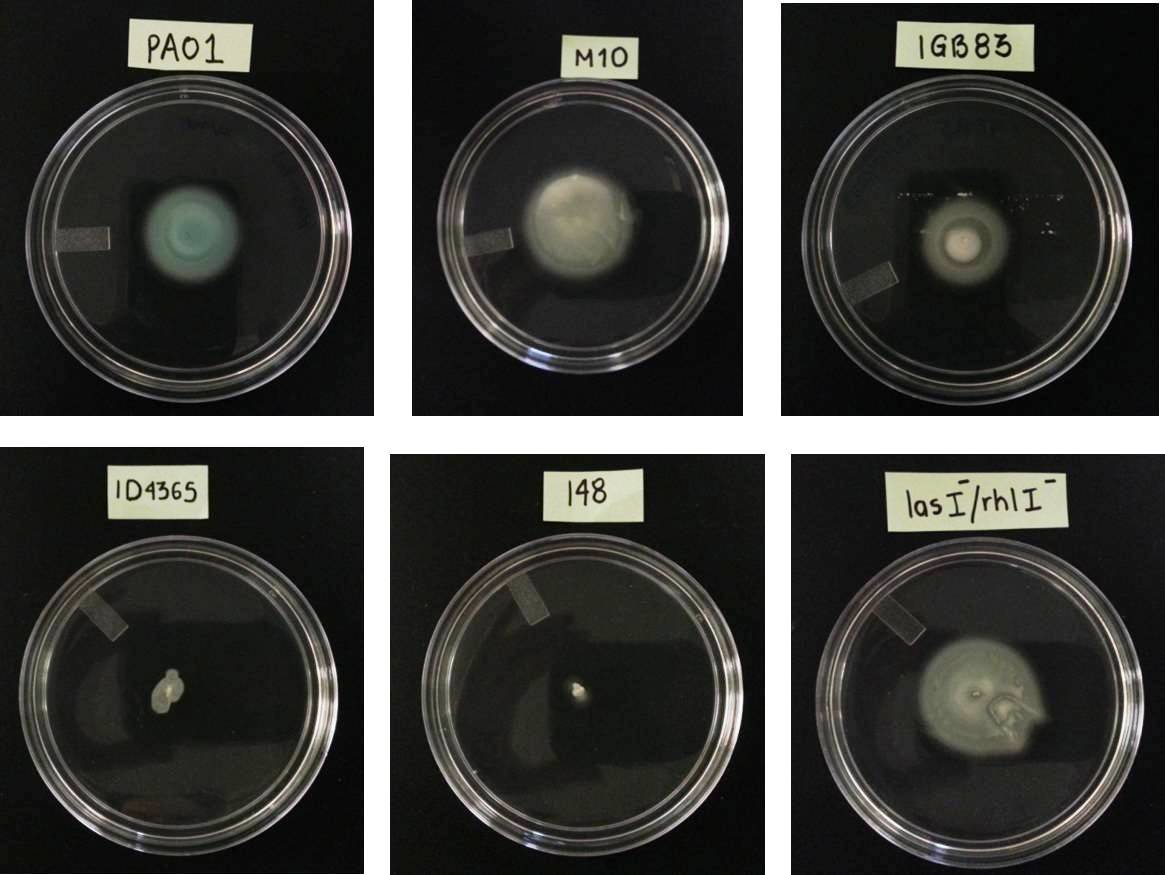


**Figure S1. Swarming and swimming motility-phenotype of *P. aeruginosa* strains.** PAO1 and *lasI* / *rhlI* mutant, were used as positive and negative controls respectively in both assays. (A) For swarming, 5 µl of each strain was inoculated on swarm plates at OD_600_ 3.0, and incubated at 30°C for 24 h. (B) For swimming, a single colony was inoculated onto the medium with a sterile toothpick, and incubated wrapped for 24 h. at 37 °C.


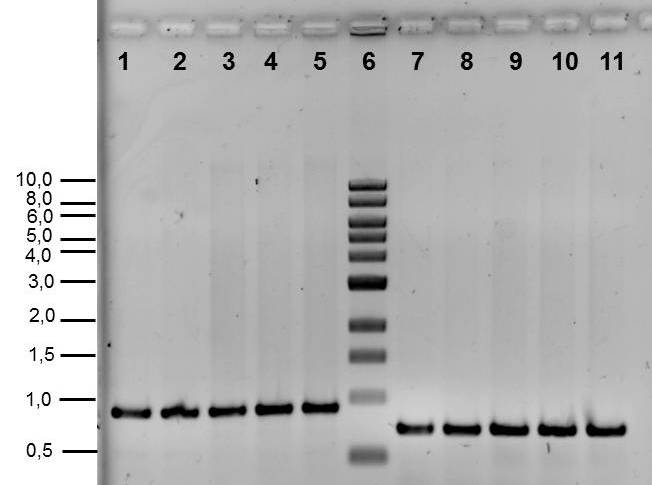


**Figure S3.** **Identification of the presence of two reiterated *phz* (*phz1* and *phz2*) operons in different *P. aeruginosa* strains by PCR amplification.** Lanes1 to 5 show the PCR product of *phz1*, using as forward primer, an oligonucleotide at the 5’ region (UTR) and as reverse primer an oligonucleotide with an internal *phzA1* as reverse primer (R-phzA). Lanes 7 to 11 show the amplification of *phz2* using as forward oligonucleotide a sequence in the phzA2 5’ UTR and reverse primer R-phzA. Strains analyzed correspond to PAO1 (lanes 1 and 7); M10 (lanes 2 and 8); IGB83 (lanes 3 and 9); 148 (lanes 4 and 10) and ID4365 (lanes 5 and 11). Lanes 6 shows molecular size standards.

1 2 3


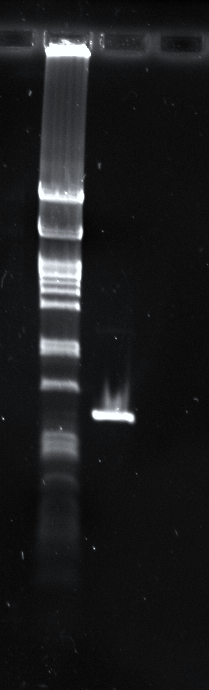


**Figure S4. Corroboration of the 20 Kb deletion in the strain 148, which includes *lasR*.**  Lane 1 shows molecular size markers, lane 2 shows the PCR product obtained using as template the genome of strain 148 and as primers oligonucleotides that are 20 kb appart in the genome of strain PAO1. Lane 3 shows that no PCR product is obtained when PAO1 genome is used as template and the same oligonucleotides as in lane 2 are used as primers.
