# Supplementary material for: Pseudomonas aeruginosa clinical and environmental isolates constitute a single population with high phenotypic diversity
Source: BMC Genomics. 2014 Apr 28;15:318. doi: 10.1186/1471-2164-15-318 (PMC4234422; doi:10.1186/1471-2164-15-318)
Supplement: Additional file 3: Figure S2 — Amino-acids sequence alignment of proteins encoded by different genes that participate on cell swimming and swarming, of strains 148, ID4365, IGB83, M10 and PAO1. [file 1471-2164-15-318-S3.pdf]

fliA

```
PAO1            MTAASGVRMYSKAQAQNSQEQLIQRYAPLVKRIAYHLLGRLPASVQVEDLMQAGMIGLLE 60
148             --------MYSKAQAQNSQEQLIQRYAPLVKRIAYHLLGRLPASVQVEDLMQAGMIGLLE 52
ID4365          --------MYSKAQAQNSQEQLIQRYAPLVKRIAYHLLGRLPASVQVEDLMQAGMIGLLE 52
IGB83           --------MYSKAQAQNSQEQLIQRYAPLVKRIAYHLLGRLPASVQVEDLMQAGMIGLLE 52
M10             --------MYSKAQAQNSQEQLIQRYAPLVKRIAYHLLGRLPASVQVEDLMQAGMIGLLE 52
                        ****************************************************

PAO1            AAKKYDAGKGASFETYAGIRIRGAMLDEVRKGDWAPRSVHRNTRMVTDAIRAIEARTGRD 120
148             AAKKYDAGKGASFETYAGIRIRGAMLDEVRKGDWAPRSVHRNTRMVTDAIRAIEARTGRD 112
ID4365          AAKKYDAGKGASFETYAGIRIRGAMLDEVRKGDWAPRSVHRNTRMVTDAIRAIEARTGRD 112
IGB83           AAKKYDAGKGASFETYAGIRIRGAMLDEVRKGDWAPRSVHRNTRMVTDAIRAIEARTGRD 112
M10             AAKKYDAGKGASFETYAGIRIRGAMLDEVRKGDWAPRSVHRNTRMVTDAIRAIEARTGRD 112
                ************************************************************

PAO1            AKDHEVAAELQLSLEDYYGILSDTQGSRLYSFDDLLQDGEHGLPEDTSLSHNEPIHGLLD 180
148             AKDHEVAAELQLSLEDYYGILSDTQGSRLYSFDDLLQDGEHGLPEDTSLSHNEPIHGLLD 172
ID4365          AKDHEVAAELQLSLEDYYGILSDTQGSRLYSFDDLLQDGEHGLPEDTSLSHNEPIHGLLD 172
IGB83           AKDHEVAAELQLSLEDYYGILSDTQGSRLYSFDDLLQDGEHGLPEDTSLSHNEPIHGLLD 172
M10             AKDHEVAAELQLSLEDYYGILSDTQGSRLYSFDDLLQDGEHGLPEDTSLSHNEPIHGLLD 172
                ************************************************************

PAO1            ERFQAALADAIAKLPERERLVLALYYDEELNLKEIGEVLGVSESRVSQLHSQCAARLRAR 240
148             ERFQAALADAIAKLPERERLVLALYYDEELNLKEIGEVLGVSESRVSQLHSQCAARLRAR 232
ID4365          ERFQAALADAIAKLPERERLVLALYYDEELNLKEIGEVLGVSESRVSQLHSQCAARLRAR 232
IGB83           ERFQAALADAIAKLPERERLVLALYYDEELNLKEIGEVLGVSESRVSQLHSQCAARLRAR 232
M10             ERFQAALADAIAKLPERERLVLALYYDEELNLKEIGEVLGVSESRVSQLHSQCAARLRAR 232
                ************************************************************

PAO1            LADWRSA 247
148             LADWRSA 239
ID4365          LADWRSA 239
IGB83           LADWRSA 239
M10             LADWRSG 239
                ******.
```

fliC

```
PAO1            -------------------------MALTVNTNIASLNTQRNLNASSNDLNTSLQRLTTG 35
M10             -------------------------MALTVNTNIASLNTQRNLNASSNDLNTSLQRLTTG 35
IGB83           MGHLSKQAERSQAQVTEIGPLEEITMALTVNTNIASLNTQRNLNASSNDLNTSLQRLTTG 60
ID4365          MGHLSKQAERSQAQVTEIGPLEEITMALTVNTNIASLNTQRNLNASSNDLNTSLQRLTTG 60
148             -------------------------MALTVNTNIASLNTQRNLNNSSASLNTSLQRLSTG 35
                                         ******************* ** .********:**

PAO1            YRINSAKDDAAGLQISNRLSNQISGLNVATRNANDGISLAQTAEGALQQSTNILQRIRDL 95
M10             YRINSAKDDAAGLQISNRLSNQISGLNVATRNANDGISLAQTAEGALQQSTNILQRIRDL 95
IGB83           YRINSAKDDAAGLQISNRLSNQISGLNVATRNANDGISLAQTAEGALQQSTNILQRIRDL 120
ID4365          YRINSAKDDAAGLQISNRLSNQISGLNVATRNANDGISLAQTAEGALQQSTNILQRIRDL 120
148             SRINSAKDDAAGLQIANRLTSQVNGLNVATKNANDGISLAQTAEGALQQSTNILQRMRDL 95
                 **************:***:.*:.******:*************************:***

PAO1            ALQSANGSNSDADRAALQKEVAAQQAELTRISDTTTFGGRKLLDGSFGTTSFQVGSNAYE 155
M10             ALQSANGSNSDADRAALQKEVAAQQAELTRISDTTTFGGRKLLDGSFGTTSFQVGSNAYE 155
IGB83           ALQSANGSNSDADRAALQKEVAAQQAELTRISDTTTFGGRKLLDGSFGTTSFQVGSNAYE 180
ID4365          ALQSANGSNSDADRAALQKEVAAQQAELTRISDTTTFGGRKLLDGSFGTTSFQVGSNAYE 180
148             SLQSANGSNSDSERTALNGEVKQLQKELDRISNTTTFGGRKLLDGSFGVASFQVGSAANE 155
                :**********::*:**: **   * ** ***:***************.:****** * *

PAO1            TIDISLQNASASAIGSYQVGSNGAGTVASVAGTATASGIASGTVNLVGGGQVKN-IAIAA 214
M10             TIDISLQNASASAIGSYQVGSNGAGTVASVAGTATASGIASGTVNLVGGGQVKN-IAIAA 214
IGB83           TIDISLQNASASAIGSYQVGSNGAGTVASVAGTATASGIASGTVNLVGGGQVKN-IAIAA 239
ID4365          TIDISLQNASASAIGSYQVGSNGAGTVASVAGTATASGIASGTVNLVGGGQVKN-IAIAA 239
148             IISVGIDEMSAESLNGTYFTATGGGAVA----AATASGTVDIAIGITGGSAVNVKVDMKG 211
                 *.:.::: **.::..  . :.*.*:**    :***** .. ::.:.**. *:  : : .

PAO1            GDSAKAIAEKMDGAIPNLSARARTVFTADVSGVTGGSLNFDVTVGSNTVSLAGVTSTQDL 274
M10             GDSAKAIAEKMDGAIPNLSARARTVFTADVSGVTGGSLNFDVTVGSNTVSLAGVTSTQDL 274
IGB83           GDSAKAIAEKMDGAIPNLSARARTVFTADVSGVTGGSLNFDVTVGSNTVSLAGVTSTQDL 299
ID4365          GDSAKAIAEKMDGAIPNLSARARTVFTADVSGVTGGSLNFDVTVGSNTVSLAGVTSTQDL 299
148             NETAEQAAAKIAAAV--------------------------------------------- 226
                .::*:  * *: .*:

PAO1            ADQLNSNSSKLGITASINDKGVLTITSATGENVKFGAQTGTATAGQVAVKVQGSDGKFEA 334
M10             ADQLNSNSSKLGITASINDKGVLTITSATGENVKFGAQTGTATAGQVAVKVQGSDGKFEA 334
IGB83           ADQLNSNSSKLGITASINDKGVLTITSATGENVKFGAQTGTATAGQVAVKVQGSDGKFEA 359
ID4365          ADQLNSNSSKLGITASINDKGVLTITSATGENVKFGAQTGTATAGQVAVKVQGSDGKFEA 359
148             ------NDANVGIGAFTDGAQISYVSKAS-------ADGTTSAVSGVAITDTGSTG---- 269
                      *.:::** *  :.  :  ::.*:       *:  *::.. **:.  ** *

PAO1            AAKNVVAAGTAATTTIVTGYVQLNSPTAYSVSGTGTQASQVFGNASAAQKSSVASVDIST 394
```

```
M10             AAKNVVAAGTAATTTIVTGYVQLNSPTAYSVSGTGTQASQVFGNASAAQKSSVASVDIST 394
IGB83           AAKNVVAAGTAATTTIVTGYVQLNSPTAYSVSGTGTQASQVFGNASAAQKSSVASVDIST 419
ID4365          AAKNVVAAGTAATTTVVTGYVQLNSPTAYSVSGTGTQASQVFGNASAAQKSSVASVDIST 419
148             -------AGTAAGTTTFT-----------------------------EANDTVAKIDIST 293
                       ***** ** .*                             :.:**.:****

PAO1            ADGAQNAIAVVDNALAAIDAQRADLGAVQNRFKNTIDNLTNISENATNARSRIKDTDFAA 454
M10             ADGAQNAIAVVDNALAAIDAQRADLGAVQNRFKNTIDNLTNISENATNARSRIKDTDFAA 454
IGB83           ADGAQNAIAVVDNALAAIDAQRADLGAVQNRFKNTIDNLTNISENATNARSRIKDTDFAA 479
ID4365          ADGAQNAIAVVDNALAAIDAQRADLGAVQNRFKNTIDNLTNISENATNARSRIKDTDFAA 479
148             AKGAQSAVLVIDEAIKQIDAQRADLGAVQNRFDNTINNLKNIGENVSAARGRIEDTDFAA 353
                *.***.*: *:*:*:  ***************.***:**.**.**.: **.**:******

PAO1            ETAALSKNQVLQQAGTAILAQANQLPQAVLSLLR 488
M10             ETAALSKNQVLQQAGTAILAQANQLPQAVLSLLR 488
IGB83           ETAALSKNQVLQQAGTAILAQANQLPQAVLSLLR 513
ID4365          ETAALSKNQVLQQAGTAILAQANQLPQAVLSLLR 513
148             ETANLTKNQVLQQAGTAILAQANQLPQSVLSLLR 387
                *** *:*********************:******
```

fliD

```
IGB83           MAG-ISIG--VGSTDYTDLVNKMVNLEGAAKTNQLATLEKTTTTRLTALGQFKSAISAFQ 57
M10             MAG-ISIG--VGSTDYTDLVNKMVNLEGAAKTNQLATLEKTTTTRLTALGQFKSAISAFQ 57
PAO1            MAG-ISIG--VGSTDYTDLVNKMVNLEGAAKTNQLATLEKTTTTRLTALGQFKSAISAFQ 57
ID4365          MAG-ISIG--VGSTDYTDLVNKMVNLEGAAKTNQLATLEKTTTTRLTALGQFKSAISAFQ 57
148             MANSTTINGYNSGLDIKNIVSTLVAAEKAPKEAQLKRLESDTTAKFTGIGQLKSAISDLQ 60
                **.  :*.    .. * .::*..:*  * *.*  **  **. **:::*.:**:***** :*

IGB83           TALTALNSNAVFMARTAKSSNEDILKASATQSAVAGTYQIQVNSLATSSKIALQAIADPA 117
M10             TALTALNSNAVFMARTAKSSNEDILKASATQSAVAGTYQIQVNSLATSSKIALQAIADPA 117
PAO1            TALTALNSNAVFMARTAKSSNEDILKASATQSAVAGTYQIQVNSLATSSKIALQAIADPA 117
ID4365          TALTALNSNAVFMARTAKSSNEDILKASATQSAVAGTYQIQVNSLATSSKIALQAIADPA 117
148             TILKELNKPELFQKRSASTSDEKFATATATKDALPGIYKLEVTQLASVSKVATASFAD-G 119
                * *. **.  :*  *:*.:*:*.: .*:**:.*:.* *:::*..**: **:*  ::** .

IGB83           NAKFNSGTLNISVGDTKLPAITVDSSNNTLAGMRDAINQAGKEAGVSATIITD--NSGSR 175
M10             NAKFNSGTLNISVGDTKLPAITVDSSNNTLAGMRDAINQAGKEAGVSATIITD--NSGSR 175
PAO1            NAKFNSGTLNISVGDTKLPAITVDSSNNTLAGMRDAINQAGKEAGVSATIITD--NSGSR 175
ID4365          NAKFNSGTLNISVGDTKLPAITVDSSNNTLAGMRDAINQAGKEAGVSATIITD--NSGSR 175
148             YKTTSGGTLTIKQGADDAGVTVNVAAGATLAEVRDSLNAQLKDKGITANIVNNPGDGTSR 179
                  . ..***.*. *  .  . .  ::. *** :**::*   *: *::*.*:.:  :. **

IGB83           LVLSSTKTGDGKDIKVEVSDDGSGGNTSLSQLAFDPATAPKLSDG---AAAGYVTKAANG 232
M10             LVLSSTKTGDGKDIKVEVSDDGSGGNTSLSQLAFDPATAPKLSDG---AAAGYVTKAANG 232
PAO1            LVLSSTKTGDGKDIKVEVSDDGSGGNTSLSQLAFDPATAPKLSDG---AAAGYVTKAANG 232
ID4365          LVLSSTKTGDGKDIKVEVSDDGSGGNTSLSQLAFDPATAPKLSDG---AAAGYVTKAANG 232
148             LVFTGKDSGAGKDVFVQGSS--GLENFNIGSVGADGKLTLSQLDGTSSSSSGYITQAKNA 237
                **::...:* ***: *: *.  .  * .:..:. *   : .  **   :::**:*:* *.

IGB83           EITVDGLKRSIASNSVSDVIDGVSFDVKAVTEAGKPITLTVSRDDAGVKDNVKKFVEAYN 292
M10             EITVDGLKRSIASNSVSDVIDGVSFDVKAVTEAGKPITLTVSRDDAGVKDNVKKFVEAYN 292
PAO1            EITVDGLKRSIASNSVSDVIDGVSFDVKAVTEAGKPITLTVSRDDAGVKDNVKKFVEAYN 292
ID4365          EITVDGLKRSIASNSVSDVIDGVSFDVKAVTEAGKPITLTVSRDDAGVKDNVKKFVEAYN 292
148             KFSIDGLTLESPTNTVDKVINGVTFELKTVTDTNKPITISVEQDRGGVKDNIKKFVEAYN 297
                ::::***. . .:*:*..**:**:*::*:**::.****::*.:* .*****:********

IGB83           TLTKFINEQTVVTKVGEDKNPVTGALLGDASVRALVNTMRSELIASNENGSVRNLAALGI 352
M10             TLTKFINEQTVVTKVGEDKNPVTGALLGDASVRALVNTMRSELIASNENGSVRNLAALGI 352
PAO1            TLTKFINEQTVVTKVGEDKNPVTGALLGDASVRALVNTMRSELIASNENGSVRNLAALGI 352
ID4365          TLTKFINEQTVVTKVGEDKTPVTGALLGDASVRALVNTMRSELIASNENGSVRNLAALGI 352
148             KLVGVTSELTGVTKVGDDKAPVVGALVGDSSVRNLLTTMRNEMVQPGQGTDVRMLADMGI 357
                .*. . .* * *****:** **.***:**:*** *:.***.*:: ..:. .** ** :**

IGB83           TTTKDGTLEIDEKKLDKAISADFEGVASYFTGDTGLAKRLGDKMKPYTDAQGILDQRTTT 412
M10             TTTKDGTLEIDEKKLDKAISADFEGVASYFTGDTGLAKRLGDKMKPYTDAQGILDQRTTT 412
PAO1            TTTKDGTLEIDEKKLDKAISADFEGVASYFTGDTGLAKRLGDKMKPYTDAQGILDQRTTT 412
ID4365          TTTKDGTLEIDEKKLDKAISADFEGVASYFTGDTGLAKRLGDKMKPYTDAQGILDQRTTT 412
148             TTKDGTLEIDDKKLDKVLKDKFESVSALFTGDTGLMKRLDDKLTPYTQTGGVLQQRLDG 417
                **.********:*****.:.  .**.*:: ******* ***.**:.***:: *:*:**

IGB83           LQKTLSNVDTQKADLAKRLAALQEKLTTQFNLLSAMQDEMTKRQKSITDNLASLPYGSGK 472
M10             LQKTLSNVDTQKADLAKRLAALQEKLTTQFNLLSAMQDEMTKRQKSITDNLASLPYGSGK 472
PAO1            LQKTLSNVDTQKADLAKRLAALQEKLTTQFNLLSAMQDEMTKRQKSITDNLASLPYGSGK 472
ID4365          LQKTLSNVDTQKADLAKRLAALQEKLTTQFNLLSAMQDEMTKRQKSITDNLASLPYGSGK 472
148             LQDTIKSVDTQRQALDRRVEQLQDRLLKQFTAMDQLIGQLNQTSGRMAQALSSLP-GLVK 476
                **.*:..****:  * :*:  **::* .**. :. : .::.: .  ::: *:*** *  *

IGB83           KT 474
M10             KT 474
PAO1            KT 474
ID4365          KT 474
148             KS 478
```

```
                *:
```

fliE

```
PAO1            MSQGVEFNRLMLEMRSMQMEAMAKAKPVQAPAEVGAPSFSEMLSQAVDKVNETQQASTAM 60
148             MSQGVEFNRLMLEMRSMQMEAMAKAKPVQAPAEVGAPSFSEMLSQAVDKVNETQQASTAM 60
ID4365          MSQGVEFNRLMLEMRSMQMEAMAKAKPVQAPAEVGAPSFSEMLSQAVDKVNETQQASTAM 60
M10             MSQGVEFNRLMLEMRSMQMEAMAKAKPVQAPAEVGAPSFSEMLSQAVDKVNETQQASTAM 60
IGB83           MSQGVEFNRLMLEMRSMQMEAMAKAKPVQAPAEAGAPSFSEMLSQAVDKVNETQQASTAM 60
                *********************************.**************************

PAO1            ANAFEVGQSGVDLTDVMIASQKASVSFQAMTQVRNKLVQAYQDIMQMPV 109
148             ANAFEVGQSGVDLTDVMIASQKASVSFQAMTQVRNKLVQAYQDIMQMPV 109
ID4365          ANAFEVGQSGVDLTDVMIASQKASVSFQAMTQVRNKLVQAYQDIMQMPV 109
M10             ANAFEVGQSGVDLTDVMIASQKASVSFQAMTQVRNKLVQAYQDIMQMPV 109
IGB83           ANAFEVGQSGVDLTDVMIASQKASVSFQAMTQVRNKLVQAYQDIMQMPV 109
                *************************************************
```

fliF

```
PAO1            MADALIDSQVPAKSGGAGMLKKSFPGLSFLDNLSEMTMLRQIGLLVGLAASVAIGFAVVL 60
148             MADALIDSQVPAKSGGAGMLKKSFPGLSFLDNLSEMTMLRQIGLLVGLAASVAIGFAVVL 60
ID4365          MADALIDSQVPAKSGGAGMLKKSFPGLSFLDNLSEMTMLRQIGLLVGLAASVAIGFAVVL 60
M10             MADALIDSQVPAKSGGAGMLKKSFPGLSFLDNLSEMTMLRQIGLLVGLAASVAIGFAVVL 60
IGB83           MADALIDSQVPAKSGGAGMLKKSFPGLSFLDNLSEMTMLRQIGLLVGLAASVAIGFAVVL 60
                ************************************************************

PAO1            WSQQPDYKPLYGSLNGVDANRVVEALTAADIPYKVEPNSGALLVKADDLGRARMKVASAG 120
148             WSQQPDYKPLYGSLNGVDANRVVEALTAADIPYKVEPNSGALLVKADDLGRARMKVASAG 120
ID4365          WSQQPDYKPLYGSLNGVDANRVVEALTAADIPYKVEPNSGALLVKADDLGRARMKVASAG 120
M10             WSQQPDYKPLYGSLNGVDANRVVEALTAADIPYKVEPNSGALLVKADDLGRARMKVASAG 120
IGB83           WSQQPDYKPLYGSLNGVDANRVVEALTAADIPYKVEPNSGALLVKADDLGRARMKVASAG 120
                ************************************************************

PAO1            VAPTDNNVGFEILDKEQALGTSQFMEATNYRRGLEGELARTVSSLNNVKAARVHLAIPKS 180
148             VAPTDNNVGFEILDKEQALGTSQFMEATNYRRGLEGELARTVSSLNNVKAARVHLAIPKS 180
ID4365          VAPTDNNVGFEILDKEQALGTSQFMEATNYRRGLEGELARTVSSLNNVKAARVHLAIPKS 180
M10             VAPTDNNVGFEILDKEQALGTSQFMEATNYRRGLEGELARTVSSLNNVKAARVHLAIPKS 180
IGB83           VAPTDNNVGFEILDKEQALGTSQFMEATNYRRGLEGELARTVSSLNNVKAARVHLAIPKS 180
                ************************************************************

PAO1            SVFVRDDRKPSASVLVELYPGRSLEPSQVMAIVNLVATSVPELDKSQVTVVDQKGNLLSD 240
148             SVFVRDDRKPSASVLVELYPGRSLEPSQVMAIVNLVATSVPELDKSQVTVVDQKGNLLSD 240
ID4365          SVFVRDDRKPSASVLVELYPGRSLEPSQVMAIVNLVATSVPELDKSQVTVVDQKGNLLSD 240
M10             SVFVRDDRKPSASVLVELYPGRSLEPSQVMAIVNLVATSVPELDKSQVTVVDQKGNLLSD 240
IGB83           SVFVRDDRKPSASVLVELYPGRSLEPSQVMAIVNLVATSVPELDKSQVTVVDQKGNLLSD 240
                ************************************************************

PAO1            QQELSELTMAGKQFDFTRRMEGLLTQRVHNILQPVLGNGRYKAEVSADVDFSAVESTSEM 300
148             QQELSELTMAGKQFDFTRRMEGLLTQRVHNILQPVLGNGRYKAEVSADVDFSAVESTSEM 300
ID4365          QQELSELTMAGKQFDFTRRMEGLLTQRVHNILQPVLGNGRYKAEVSADVDFSAVESTSEM 300
M10             QQELSELTMAGKQFDFTRRMEGLLTQRVHNILQPVLGNGRYKAEVSADVDFSAVESTSEM 300
IGB83           QQELSELTMAGKQFDFTRRMEGLLTQRVHNILQPVLGNGRYKAEVSADVDFSAVESTSEM 300
                ************************************************************

PAO1            YNPDQPALRSEQRNNEERQNSSGPQGVPGALSNQPPGPASAPQQATASAPADYVAPGQPL 360
148             YNPDQPALRSEQRNNEERQNSSGPQGVPGALSNQPPGPASAPQQATASAPADYVAPGQPL 360
ID4365          YNPDQPALRSEQRNNEERQNSSGPQGVPGALSNQPPGPASAPQQATASAPADYVAPGQPL 360
M10             YNPDQPALRSEQRNNEERQNSSGPQGVPGALSNQPPGPASAPQQATASAPADYVAPGQPL 360
IGB83           YNPDQPALRSEQRNNEERQNSSGPQGVPGALSNQPPGPASAPQQATASAPADYVAPGQPL 360
                ************************************************************

PAO1            KDANGQTIIDPKTGKPELAPYPTDKRDQTTRNYELDRSISYTKQQQGRLRRLSVAVVLDD 420
148             KDANGQTIIDPKTGKPELAPYPTDKRDQTTRNYELDRSISYTKQQQGRLRRLSVAVVLDD 420
ID4365          KDANGQTIIDPKTGKPELAPYPTDKRDQTTRNYELDRSISYTKQQQGRLRRLSVAVVLDD 420
M10             KDANGQTIIDPKTGKPELAPYPTDKRDQTTRNYELDRSISYTKQQQGRLRRLSVAVVLDD 420
IGB83           KDANGQTIIDPKTGKPELAPYPTDKRDQTTRNYELDRSISYTKQQQGRLRRLSVAVVLDD 420
                ************************************************************

PAO1            QMKVDAKTGEVSHQPWSADELARFTRLVQDSVGYDASRGDSVSVINAPFAPAQAEEIDSI 480
148             QMKVDAKTGEVSHQPWSADELARFTRLVQDSVGYDASRGDSVSVINAPFAPAQAEEIDSI 480
ID4365          QMKVDAKTGEVSHQPWSADELARFTRLVQDSVGYDASRGDSVSVINAPFAPAQAEEIDSI 480
M10             QMKVDAKTGEVSHQPWSADELARFTRLVQDSVGYDASRGDSVSVINAPFAPAQAEEIDSI 480
IGB83           QMKVDAKTGEVSHQPWSADELARFTRLVQDSVGYDASRGDSVSVINAPFAPAQAEEIDSI 480
                ************************************************************

PAO1            PFYSQPWFWDIVKQVLGVLFILVLVFGVLRPVLSNITGGGKGKSLAGGGGRDGDLALGES 540
148             PFYSQPWFWDIVKQVLGVLFILVLVFGVLRPVLSNITGGGKGKSLAGGGGRDGDLALGES 540
ID4365          PFYSQPWFWDIVKQVLGVLFILVLVFGVLRPVLSNITGGGKGKSLAGGGGRDGDLALGES 540
M10             PFYSQPWFWDIVKQVLGVLFILVLVFGVLRPVLSNITGGGKGKSLAGGGGRDGDLALGES 540
IGB83           PFYSQPWFWDIVKQVLGVLFILVLVFGVLRPVLSNITGGGKGKSLAGGGGRDGDLALGES 540
                ************************************************************
```

```
PAO1            GLEGSLADDRVSIGGPSSILLPSPTEGYDAQLNAIKNLVAQDPGRVAQVVKEWINADE 598
148             GLEGSLADDRVSIGGPSSILLPSPTEGYDAQLNAIKNLVAQDPGRVAQVVKEWINADE 598
ID4365          GLEGSLADDRVSIGGPSSILLPSPTEGYDAQLNAIKNLVAQDPGRVAQVVKEWINADE 598
M10             GLEGSLADDRVSIGGPSSILLPSPTEGYDAQLNAIKNLVAQDPGRVAQVVKEWINADE 598
IGB83           GLEGSLADDRVSIGGPSSILLPSPTEGYDAQLNAIKNLVAQDPGRVAQVVKEWINADE 598
                **********************************************************
```

fliG

```
PAO1            MSENRLAAKLTKVDKAAILLLSLGETDAAQVLRHMGPKEVQRVGVAMASMRNVHREQVEQ 60
148             MSENRLAAKLTKVDKAAILLLSLGETDAAQVLRHMGPKEVQRVGVAMASMRNVHREQVEQ 60
ID4365          MSENRLAAKLTKVDKAAILLLSLGETDAAQVLRHMGPKEVQRVGVAMASMRNVHREQVEQ 60
M10             MSENRLAAKLTKVDKAAILLLSLGETDAAQVLRHMGPKEVQRVGVAMASMRNVHREQVEQ 60
IGB83           MSENRLAAKLTKVDKAAILLLSLGETDAAQVLRHMGPKEVQRVGVAMASMRNVHREQVEQ 60
                ************************************************************

PAO1            VMGEFVEVVGDQTSLGVGADGYIRKMLTQALGEDKANNLIDRILLGGSTSGLDSLKWMEP 120
148             VMGEFVEVVGDQTSLGVGADGYIRKMLTQALGEDKANNLIDRILLGGSTSGLDSLKWMEP 120
ID4365          VMGEFVEVVGDQTSLGVGADGYIRKMLTQALGEDKANNLIDRILLGGSTSGLDSLKWMEP 120
M10             VMGEFVEVVGDQTSLGVGADGYIRKMLTQALGEDKANNLIDRILLGGSTSGLDSLKWMEP 120
IGB83           VMGEFVEVVGDQTSLGVGADGYIRKMLTQALGEDKANNLIDRILLGGSTSGLDSLKWMEP 120
                ************************************************************

PAO1            RAVADVIRYEHPQIQAIVVAYLDPDQAAEVLSHFDHKVRLDIVLRVSSLNTVQPSALKEL 180
148             RAVADVIRYEHPQIQAIVVAYLDPDQAAEVLSHFDHKVRLDIVLRVSSLNTVQPSALKEL 180
ID4365          RAVADVIRYEHPQIQAIVVAYLDPDQAAEVLSHFDHKVRLDIVLRVSSLNTVQPSALKEL 180
M10             RAVADVIRYEHPQIQAIVVAYLDPDQAAEVLSHFDHKVRLDIVLRVSSLNTVQPSALKEL 180
IGB83           RAVADVIRYEHPQIQAIVVAYLDPDQAAEVLSHFDHKVRLDIVLRVSSLNTVQPSALKEL 180
                ************************************************************

PAO1            NLILEKQFAGNSNATRTTMGGVKRAADIMNYLDSSIEGQLMDSIREVDEDLSGQIEDLMF 240
148             NLILEKQFAGNSNATRTTMGGVKRAADIMNYLDSSIEGQLMDSIREVDEDLSGQIEDLMF 240
ID4365          NLILEKQFAGNSNATRTTMGGVKRAADIMNYLDSSIEGQLMDSIREVDEDLSGQIEDLMF 240
M10             NLILEKQFAGNSNATRTTMGGVKRAADIMNYLDSSIEGQLMDSIREVDEDLSGQIEDLMF 240
IGB83           NLILEKQFAGNSNATRTTMGGVKRAADIMNYLDSSIEGQLMDSIREVDEDLSGQIEDLMF 240
                ************************************************************

PAO1            VFDNLADVDDRGIQALLREVSSDVLVLALKGSDEAIREKVFKNMSKRAAELLRDDLEAKG 300
148             VFDNLADVDDRGIQALLREVSSDVLVLALKGSDEAIREKVFKNMSKRAAELLRDDLEAKG 300
ID4365          VFDNLADVDDRGIQALLREVSSDVLVLALKGSDEAIREKVFKNMSKRAAELLRDDLEAKG 300
M10             VFDNLADVDDRGIQALLREVSSDVLVLALKGSDEAIREKVFKNMSKRAAELLRDDLEAKG 300
IGB83           VFDNLADVDDRGIQALLREVSSDVLVLALKGSDEAIREKVFKNMSKRAAELLRDDLEAKG 300
                ************************************************************

PAO1            PVRVSEVEGAQKEILTIARRMAESGDIVLGGKGGEEMI 338
148             PVRVSEVEGAQKEILTIARRMAESGDIVLGGKGGEEMI 338
ID4365          PVRVSEVEGAQKEILTIARRMAESGDIVLGGKGGEEMI 338
M10             PVRVSEVEGAQKEILTIARRMAESGDIVLGGKGGEEMI 338
IGB83           PVRVSEVEGAQKEILTIARRMAESGDIVLGGKGGEEMI 338
                **************************************
```

fliI

```
PAO1            MRLERTSFARRLEGYTEAVSLPAQPVVEGRLLRMVGLTLEAEGLQAAVGSRCNVINESGY 60
148             MRLERTSFARRLEGYTEAVSLPAQPVVEGRLLRMVGLTLEAEGLQAAVGSRCNVINESGY 60
ID4365          MRLERTSFARRLEGYTEAVSLPAQPVVEGRLLRMVGLTLEAEGLQAAVGSRCNVINESGY 60
M10             MRLERTSFARRLEGYTEAVSLPAQPVVEGRLLRMVGLTLEAEGLQAAVGSRCNVINESGY 60
IGB83           MRLERTSFARRLEGYTEAVSLPAQPVVEGRLLRMVGLTLEAEGLQAAVGSRCNVINESGY 60
                ************************************************************

PAO1            HPVQVEAEVMGFSGSKVYLMPVGSLAGIAPGARVVPLPDTGRLPMGMSMLGRVLDGAGRA 120
148             HPVQVEAEVMGFSGSKVYLMPVGSLAGIAPGARVVPLPDTGRLPMGMSMLGRVLDGAGRA 120
ID4365          HPVQVEAEVMGFSGSKVYLMPVGSLAGIAPGARVVPLPDTGRLPMGMSMLGRVLDGAGRA 120
M10             HPVQVEAEVMGFSGSKVYLMPVGSLAGIAPGARVVPLPDTGRLPMGMSMLGRVLDGAGRA 120
IGB83           HPVQVEAEVMGFSGSKVYLMPVGSLAGIAPGARVVPLPDTGRLPMGMSMLGRVLDGAGRA 120
                ************************************************************

PAO1            LDGKGGMRAEDWVPMDGPTINPLKRHPISEPLDVGIRSINGLLTVGRGQRLGLFAGTGVG 180
148             LDGKGGMRAEDWVPMDGPTINPLKRHPISEPLDVGIRSINGLLTVGRGQRLGLFAGTGVG 180
ID4365          LDGKGGMRAEDWVPMDGPTINPLKRHPISEPLDVGIRSINGLLTVGRGQRLGLFAGTGVG 180
M10             LDGKGGMRAEDWVPMDGPTINPLKRHPISEPLDVGIRSINGLLTVGRGQRLGLFAGTGVG 180
IGB83           LDGKGGMRAEDWVPMDGPTINPLKRHPISEPLDVGIRSINGLLTVGRGQRLGLFAGTGVG 180
                ************************************************************

PAO1            KSVLLGMMTRFTRADIIVVGLIGERGREVKEFIDEILGEEGLKRSVVVASPADDAPLMRL 240
148             KSVLLGMMTRFTRADIIVVGLIGERGREVKEFIDEILGEEGLKRSVVVASPADDAPLMRL 240
ID4365          KSVLLGMMTRFTRADIIVVGLIGERGREVKEFIDEILGEEGLKRSVVVASPADDAPLMRL 240
M10             KSVLLGMMTRFTRADIIVVGLIGERGREVKEFIDEILGEEGLKRSVVVASPADDAPLMRL 240
IGB83           KSVLLGMMTRFTRADIIVVGLIGERGREVKEFIDEILGEEGLKRSVVVASPADDAPLMRL 240
                ************************************************************

PAO1            RAAQYCTRIAEYFRDKGKNVLLLMDSLTRYAQAQREIALAIGEPPATKGYPPSVFAKLPK 300
148             RAAQYCTRIAEYFRDKGKNVLLLMDSLTRYAQAQREIALAIGEPPATKGYPPSVFAKLPK 300
ID4365          RAAQYCTRIAEYFRDKGKNVLLLMDSLTRYAQAQREIALAIGEPPATKGYPPSVFAKLPK 300
```

```
M10             RAAQYCTRIAEYFRDKGKNVLLLMDSLTRYAQAQREIALAIGEPPATKGYPPSVFAKLPK 300
IGB83           RAAQYCTRIAEYFRDKGKNVLLLMDSLTRYAQAQREIALAIGEPPATKGYPPSVFAKLPK 300
                ************************************************************

PAO1            LVERAGNAEAGGGSITAFYTVLSEGDDQQDPIADAARGVLDGHFVLSRRLAEEGHYPAID 360
148             LVERAGNAEAGGGSITAFYTVLSEGDDQQDPIADAARGVLDGHFVLSRRLAEEGHYPAID 360
ID4365          LVERAGNAEAGGGSITAFYTVLSEGDDQQDPIADAARGVLDGHFVLSRRLAEEGHYPAID 360
M10             LVERAGNAEAGGGSITAFYTVLSEGDDQQDPIADAARGVLDGHFVLSRRLAEEGHYPAID 360
IGB83           LVERAGNAEAGGGSITAFYTVLSEGDDQQDPIADAARGVLDGHFVLSRRLAEEGHYPAID 360
                ************************************************************

PAO1            IEASISRVMPQVVEAEHLRDAQRFKQLWSRYQQSRDLISVGAYVAGGDPETDLAIARFPV 420
148             IEASISRVMPQVVEAEHLRDAQRFKQLWSRYQQSRDLISVGAYVAGGDPETDLAIARFPV 420
ID4365          IEASISRVMPQVVEAEHLRDAQRFKQLWSRYQQSRDLISVGAYVAGGDPETDLAIARFPV 420
M10             IEASISRVMPQVVEAEHLRDAQRFKQLWSRYQQSRDLISVGAYVAGGDPETDLAIARFPV 420
IGB83           IEASISRVMPQVVEAEHLRDAQRFKQLWSRYQQSRDLISVGAYVAGGDPETDLAIARFPV 420
                ************************************************************

PAO1            MRQFLRQGLDESESLAESRARLASLLAGGQA 451
148             MRQFLRQGLDESESLAESRARLASLLAGGQA 451
ID4365          MRQFLRQGLDESESLAESRARLASLLAGGQA 451
M10             MRQFLRQGLDESESLAESRARLASLLAGGQA 451
IGB83           MRQFLRQGLDESESLAESRARLASLLAGGQA 451
                *******************************
```

fliJ

```
IGB83           MEKRAARLAPVVDMASKAERDAATQLGRCQQQLLAAQQKLAELERYRNDYQQQWISQGQK 60
M10             MEKRAARLAPVVDMASKAERDAATQLGRCQQQLLAAQQKLAELERYRNDYQQQWISQGQK 60
PAO1            MEKRAARLAPVVDMASKAERDAATQLGRCQQQLLAAQQKLAELERYRNDYQQQWISQGQK 60
148             MEKRAARLAPVVDMASKAERDAATQLGRCQQQLLAAQQKLAELERYRNDYQQQWISQGQK 60
ID4365          MEKRAARLAPVVDMASKAERDAATQLGRCQQQLLAAQQKLAELERYRNDYQQQWISQGQK 60
                ************************************************************

IGB83           GVSGQWLMNYQRFLSQLETAVAQQANSVTWHREAVDKARLNWQERYARLEGLRKLVERYL 120
M10             GVSGQWLMNYQRFLSQLETAVAQQANSVTWHREAVDKARLNWQERYARLEGLRKLVERYL 120
PAO1            GVSGQWLMNYQRFLSQLETAVAQQANSVTWHREAVDKARLNWQERYARLEGLRKLVERYL 120
148             GVSGQWLMNYQRFLSQLETAVVQQANSVTWHREAVDKARLNWQERYARLEGLRKLVERYL 120
ID4365          GVSGQWLMNYQRFLSQLETAVVQQANSVTWHREAVDKARLNWQERYARLEGLRKLVERYL 120
                *********************.**************************************

IGB83           EEARQAEDKREQKQLDELAQRTRRQDD 147
M10             EEARQAEDKREQKQLDELAQRTRRQDD 147
PAO1            EEARQAEDKREQKQLDELAQRTRRQDD 147
148             EEARQAEDKREQKQLDELAQRTRRQDD 147
ID4365          EEARQAEDKREQKQLDELAQRTRRQDD 147
                ***************************
```

fliM

```
PAO1            MAVQDLLSQDEIDALLHGVDDGLVETEVEATPGSVKSYDLTSQDRIVRGRMPTLEMINER 60
148             MAVQDLLSQDEIDALLHGVDDGLVETEVEATPGSVKSYDLTSQDRIVRGRMPTLEMINER 60
ID4365          MAVQDLLSQDEIDALLHGVDDGLVETEVEATPGSVKSYDLTSQDRIVRGRMPTLEMINER 60
M10             MAVQDLLSQDEIDALLHGVDDGLVETEVEATPGSVKSYDLTSQDRIVRGRMPTLEMINER 60
IGB83           MAVQDLLSQDEIDALLHGVDDGLVETEVEATPGSVKSYDLTSQDRIVRGRMPTLEMINER 60
                ************************************************************

PAO1            FARYTRISMFNLLRRSADVAVGGVQVMKFGEYVHSLYVPTSLNLVKMKPLRGTALFILDA 120
148             FARYTRISMFNLLRRSADVAVGGVQVMKFGEYVHSLYVPTSLNLVKMKPLRGTALFILDA 120
ID4365          FARYTRISMFNLLRRSADVAVGGVQVMKFGEYVHSLYVPTSLNLVKMKPLRGTALFILDA 120
M10             FARYTRISMFNLLRRSADVAVGGVQVMKFGEYVHSLYVPTSLNLVKMKPLRGTALFILDA 120
IGB83           FARYTRISMFNLLRRSADVAVGGVQVMKFGEYVHSLYVPTSLNLVKMKPLRGTALFILDA 120
                ************************************************************

PAO1            KLVFKLVDNFFGGDGRHAKIEGREFTPTELRVVRMVLEQAFVDLKEAWQAVLEMNFEYVN 180
148             KLVFKLVDNFFGGDGRHAKIEGREFTPTELRVVRMVLEQAFVDLKEAWQAVLEMNFEYVN 180
ID4365          KLVFKLVDNFFGGDGRHAKIEGREFTPTELRVVRMVLEQAFVDLKEAWQAVLEMNFEYVN 180
M10             KLVFKLVDNFFGGDGRHAKIEGREFTPTELRVVRMVLEQAFVDLKEAWQAVLEMNFEYVN 180
IGB83           KLVFKLVDNFFGGDGRHAKIEGREFTPTELRVVRMVLEQAFVDLKEAWQAVLEMNFEYVN 180
                ************************************************************

PAO1            SEVNPAMANIVSPSEVVVVSTFHIELDGGGGDLHITMPYSMIEPIREMLDAGFQSDHDDQ 240
148             SEVNPAMANIVSPSEVVVVSTFHIELDGGGGDLHITMPYSMIEPIREMLDAGFQSDHDDQ 240
ID4365          SEVNPAMANIVSPSEVVVVSTFHIELDGGGGDLHITMPYSMIEPIREMLDAGFQSDHDDQ 240
M10             SEVNPAMANIVSPSEVVVVSTFHIELDGGGGDLHITMPYSMIEPIREMLDAGFQSDHDDQ 240
IGB83           SEVNPAMANIVSPSEVVVVSTFHIELDGGGGDLHITMPYSMIEPIREMLDAGFQSDHDDQ 240
                ************************************************************

PAO1            DERWIKALREDVLDVQVPLGATVVRRQLKLRDILHMQPGDVIPVEMPEHMVMRANGVPAF 300
148             DERWIKALREDVLDVQVPLGATVVRRQLKLRDILHMQPGDVIPVEMPEHMVMRANGVPAF 300
ID4365          DERWIKALREDVLDVQVPLGATVVRRQLKLRDILHMQPGDVIPVEMPEHMVMRANGVPAF 300
M10             DERWIKALREDVLDVQVPLGATVVRRQLKLRDILHMQPGDVIPVEMPEHMVMRANGVPAF 300
IGB83           DERWIKALREDVLDVQVPLGATVVRRQLKLRDILHMQPGDVIPVEIPEHMVMRANGVPAF 300
```

```
               ********************************************:**************

PAO1           KVKLGAHKGNLALQILEAVERSR 323
148            KVKLGAHKGNLALQILEAVERSR 323
ID4365         KVKLGAHKGNLALQILEAVERSR 323
M10            KVKLGAHKGNLALQILEAVERSR 323
IGB83          KVKLGAHKGNLALQILEAVERSR 323
               ***********************
```

fliN

```
PAO1           MADEEKVTTEEQALADEWAAALAEAGDASQDDIDALMAQGGATPVAEPSTPRAPMEEFGA 60
148            MADEEKVTTEEQALADEWAAALAEAGDASQDDIDALMAQGGATPVAEPSTPRAPMEEFGA 60
ID4365         MADEEKVTTEEQALADEWAAALAEAGDASQDDIDALMAQGGATPVAEPSTPRAPMEEFGA 60
M10            MADEEKVTTEEQALADEWAAALAEAGDASQDDIDALMAQGGATPVAEPSTPRAPMEEFGA 60
IGB83          MADEEKVTTEEQALADEWAAALAEAGDASQDDIDALMAQGGATPVAEPSTPRAPMEEFGA 60
               ************************************************************

PAO1           SPKAPTISGLEGPNLDVILDIPVTISMEVGHTDISIRNLLQLNQGSVIELDRLAGEPLDV 120
148            SPKAPTISGLEGPNLDVILDIPVTISMEVGHTDISIRNLLQLNQGSVIELDRLAGEPLDV 120
ID4365         SPKAPTISGLEGPNLDVILDIPVTISMEVGHTDISIRNLLQLNQGSVIELDRLAGEPLDV 120
M10            SPKAPTISGLEGPNLDVILDIPVTISMEVGHTDISIRNLLQLNQGSVIELDRLAGEPLDV 120
IGB83          SPKAPTISGLEGPNLDVILDIPVTISMEVGHTDISIRNLLQLNQGSVIELDRLAGEPLDV 120
               ************************************************************

PAO1           LVNGTLIAHGEVVVVNEKFGIRLTDVISPSERIKKLR 157
148            LVNGTLIAHGEVVVVNEKFGIRLTDVISPSERIKKLR 157
ID4365         LVNGTLIAHGEVVVVNEKFGIRLTDVISPSERIKKLR 157
M10            LVNGTLIAHGEVVVVNEKFGIRLTDVISPSERIKKLR 157
IGB83          LVNGTLIAHGEVVVVNEKFGIRLTDVISPSERIKKLR 157
               *************************************
```

fliO

```
IGB83          MRRYLFAGFLPALASLSAPLCAAEGTTGAAAPTVGAASGAAAQLAQLVLGLGLVIGLIFL 60
M10            MRRYLFAGFLPALASLSAPLCAAEGTTGAAAPTVGAASGAAAQLAQLVLGLGLVIGLIFL 60
ID4365         MRRYLFAGFLPALASLSAPLCAAEGTTGAAAPTVGAASGAAAQLAQLVLGLGLVIGLIFL 60
PAO1           MRRYLFAGFLPALASLSAPLCAAEGTTGAAAPTVGAASGAAAQLAQLVLGLGLVIGLIFL 60
148            MRRYLFAGFLPALASLSAPLCAAEGTTGAAAPTVGAASGAAAQLAQLVLGLGLVIGLIFL 60
               ************************************************************

IGB83          LAWLVRRVQQAGPRGNRLIRTLASQPLGPRDRLVLVQVGEEQILLGLTPGRITPLHVLKE 120
M10            LAWLVRRVQQAGPRGNRLIRTLASQPLGPRDRLVLVQVGEEQILLGLTPGRITPLHVLKE 120
ID4365         LAWLVRRVQQAGPRGNRLIRTLASQPLGPRDRLVLVQVGEEQILLGLTPGRITPLHVLKE 120
PAO1           LAWLVRRVQQAGPRGNRLIRTLASQPLGPRDRLVLVQVGEEQILLGLTPGRITPLHVLKE 120
148            LAWLVRRVQQAGPRGNRLIRTLASQPLGPRDRLVLVQVGEEQILLGLTPGRITPLHVLKE 120
               ************************************************************

IGB83          PVHLPDGEPATPEFAQRLLELLNKDPKGKP 150
M10            PVHLPDGEPATPEFAQRLLELLNKDPKGKP 150
ID4365         PVHLPDGEPATPEFAQRLLELLNKDPKGKP 150
PAO1           PVHLPDGEPATPEFAQRLLELLNKDPKGKP 150
148            PIHLPDGEPATPEFAQRLLELLNKDPKGKP 150
               *:****************************
```

fliP

```
PAO1           MTPRISLAGALAALCLLLLAPWPALAADPTSISAITVTTNGQGQQEYSVSLQILLIMTAL 60
148            MTPRISLAGALAALCLLLLAPWPALAADPTSISAITVTTNGQGQQEYSVSLQILLIMTAL 60
ID4365         MTPRISLAGALAALCLLLLAPWPALAADPTSISAITVTTNGQGQQEYSVSLQILLIMTAL 60
M10            MTPRISLAGALAALCLLLLAPWPALAADPTSISAITVTTNGQGQQEYSVSLQILLIMTAL 60
IGB83          MTPRISLAGALAALCLLLLAPWPALAADPTSISAITVTTNGQGQQEYSVSLQILLIMTAL 60
               ************************************************************

PAO1           SFIPAFVMLMTSFTRIIIVFSILRQALGLQSTPSNQVLVGLALFLTMFVMAPVFDKINSQ 120
148            SFIPAFVMLMTSFTRIIIVFSILRQALGLQSTPSNQVLVGLALFLTMFVMAPVFDKINSQ 120
ID4365         SFIPAFVMLMTSFTRIIIVFSILRQALGLQSTPSNQVLVGLALFLTMFVMAPVFDKINSQ 120
M10            SFIPAFVMLMTSFTRIIIVFSILRQALGLQSTPSNQVLVGLALFLTMFVMAPVFDKINSQ 120
IGB83          SFIPAFVMLMTSFTRIIIVFSILRQALGLQSTPSNQVLVGLALFLTMFVMAPVFDKINSQ 120
               ************************************************************

PAO1           ALQPYLNEQIPAQEALQKAEVPLKAFMLAQTRTSDLELFVRLSKRTDIGSPEATPLTILV 180
148            ALQPYLNEQIPAQEALQKAEVPLKAFMLAQTRTSDLELFVRLSKRTDIGSPEATPLTILV 180
ID4365         ALQPYLNEQIPAQEALQKAEVPLKAFMLAQTRTSDLELFVRLSKRTDIGSPEATPLTILV 180
M10            ALQPYLNEQIPAQEALQKAEVPLKAFMLAQTRTSDLELFVRLSKRTDIGSPEATPLTILV 180
IGB83          ALQPYLNEQIPAQEALQKAEVPLKAFMLAQTRTSDLELFVRLSKRTDIGSPEATPLTILV 180
               ************************************************************

PAO1           PAFVTSELKTAFQIGFMIFIPFLIIDLVVSSVLMAMGMMMLSPLIISLPFKIMLFVLVDG 240
148            PAFVTSELKTAFQIGFMIFIPFLIIDLVVSSVLMAMGMMMLSPLIISLPFKIMLFVLVDG 240
ID4365         PAFVTSELKTAFQIGFMIFIPFLIIDLVVSSVLMAMGMMMLSPLIISLPFKIMLFVLVDG 240
M10            PAFVTSELKTAFQIGFMIFIPFLIIDLVVSSVLMAMGMMMLSPLIISLPFKIMLFVLVDG 240
IGB83          PAFVTSELKTAFQIGFMIFIPFLIIDLVVSSVLMAMGMMMLSPLIISLPFKIMLFVLVDG 240
```

```
                 ************************************************************

PAO1             WALIIGTLAGSFGTV 255
148              WALIIGTLAGSFGTV 255
ID4365           WALIIGTLAGSFGTV 255
M10              WALIIGTLAGSFGTV 255
IGB83            WALIIGTLAGSFGTV 255
                 ***************
```

fliQ

```
PAO1             MTPEVALDLFREALWLTAMIVGVLVVPSLLVGLVVAMFQAATQINEQTLSFLPRLMVILL 60
148              MTPEVALDLFREALWLTAMIVGVLVVPSLLVGLVVAMFQAATQINEQTLSFLPRLMVILL 60
ID4365           MTPEVALDLFREALWLTAMIVGVLVVPSLLVGLVVAMFQAATQINEQTLSFLPRLMVILL 60
M10              MTPEVALDLFREALWLTAMIVGVLVVPSLLVGLVVAMFQAATQINEQTLSFLPRLMVILL 60
IGB83            MTPEVALDLFREALWLTAMIVGVLVVPSLLVGLVVAMFQAATQINEQTLSFLPRLMVILL 60
                 ************************************************************

PAO1             TLIVLGPWLLRQLMEYTQTLIGNIPLLIG 89
148              TLIVLGPWLLRQLMEYTQTLIGNIPLLIG 89
ID4365           TLIVLGPWLLRQLMEYTQTLIGNIPLLIG 89
M10              TLIVLGPWLLRQLMEYTQTLIGNIPLLIG 89
IGB83            TLIVLGPWLLRQLMEYTQTLIGNIPLLIG 89
                 *****************************
```

fliR

```
ID4365           MLELTNAQIGGWIASFVLPLFRVAALLMTMPVIGTQLVPVRVRLYLALGVCVVLVPNLPP 60
IGB83            MLELTNAQIGGWIASFVLPLFRVAALLMTMPVIGTQLVPVRVRLYLALGVCVVLVPNLPP 60
148              MLELTNAQIGGWIASFVLPLFRVAALLMTMPVIGTQLVPVRVRLYLALGVCVVLVPNLPP 60
PAO1             MLELTNAQIGGWIASFVLPLFRVAALLMTMPVIGTQLVPVRVRLYLALGVCVVLVPNLPS 60
M10              MLELTNAQIGGWIASFVLPLFRVAALLMTMPVIGTQLVPVRVRLYLALGVCVVLVPNLPS 60
                 ***********************************************************.

ID4365           MPQVDALSMKAMLLIGEQILVGALLGFSLQLLFHAFVIAGQIISMQMGLGFASMVDPANG 120
IGB83            MPQVDALSMKAMLLIGEQILVGALLGFSLQLLFHAFVIAGQIISMQMGLGFASMVDPANG 120
148              MPQVDALSMKAMLLIGEQILVGALLGFSLQLLFHAFVIAGQIISMQMGLGFASMVDPANG 120
PAO1             MPQVDALSMKAMLLIGEQILVGALLGFSLQLLFHAFVIAGQIISMQMGLGFASMVDPANG 120
M10              MPQVDALSMKAMLLIGEQILVGALLGFSLQLLFHAFVIAGQIISMQMGLGFASMVDPANG 120
                 ************************************************************

ID4365           VSVPVLGQFFTMLVTLLFLAMNGHLVVFEVIAESFVTLPVGEGLSGNHFWIIAGKLGWVM 180
IGB83            VSVPVLGQFFTMLVTLLFLAMNGHLVVFEVIAESFVTLPVGEGLSGNHFWIIAGKLGWVM 180
148              VSVPVLGQFFTMLVTLLFLAMNGHLVVFEVIAESFVTLPVGEGLSGNHFWIIAGKLGWVM 180
PAO1             VSVPVLGQFFTMLVTLLFLAMNGHLVVFEVIAESFVTLPVGEGLSGNHFWIIAGKLGWVM 180
M10              VSVPVLGQFFTMLVTLLFLAMNGHLVVFEVIAESFVTLPVGEGLSGNHFWIIAGKLGWVM 180
                 ************************************************************

ID4365           GAALLLALPAITALLVVNLAFGAMTRAAPQLNIFSIGFPLTLVLGLVILWIGTADLLSQY 240
IGB83            GAALLLALPAITALLVVNLAFGAMTRAAPQLNIFSIGFPLTLVLGLVILWIGTADLLSQY 240
148              GAALLLALPAITALLVVNLAFGAMTRAAPQLNIFSIGFPLTLVLGLVILWIGTADLLSQY 240
PAO1             GAALLLALPAITALLVVNLAFGAMTRAAPQLNIFSIGFPLTLVLGLVILWIGTADLLSQY 240
M10              GAALLLALPAITALLVVNLAFGAMTRAAPQLNIFSIGFPLTLVLGLVILWIGTADLLSQY 240
                 ************************************************************

ID4365           QVLAGEALQFLRELVRAK 258
IGB83            QVLAGEALQFLRELVRAK 258
148              QVLAGEALQFLRELVRAK 258
PAO1             QVLAGEALQFLRELVRAK 258
M10              QVLAGEALQFLRELVRAK 258
                 ******************
```

flgB

```
PAO1             MSISFDRALGIHQQALSFRAQRAEVLANNLANADTPNYKARDLDFAAVLAEQKDKAAKGT 60
148              MSISFDRALGIHQQALSFRAQRAEVLANNLANADTPNYKARDLDFAAVLAEQKDKAAKGT 60
ID4365           MSISFDRALGIHQQALSFRAQRAEVLANNLANADTPNYKARDLDFAAVLAEQKDKAAKGT 60
M10              MSISFDRALGIHQQALSFRAQRAEVLANNLANADTPNYKARDLDFAAVLAEQKDKAAKGT 60
IGB83            MSISFDRALGIHQQALSFRAQRAEVLANNLANADTPNYKARDLDFAAVLAEQKDKAAKGT 60
                 ************************************************************

PAO1             FATRTTNERHIAAEGFGMGEGSLQYRIPVQPSIDQNTVDAQIEQANYAENAMHFQASFTF 120
148              FATRTTNERHIAAEGFGMGEGSLQYRIPVQPSIDQNTVDAQIEQANYAENAMHFQASFTF 120
ID4365           FATRTTNERHIAAEGFGMGEGSLQYRIPVQPSIDQNTVDAQIEQANYAENAMHFQASFTF 120
M10              FATRTTNERHIAAEGFGMGEGSLQYRIPVQPSIDQNTVDAQIEQANYAENAMHFQASFTF 120
IGB83            FATRTTNERHIAAEGFGMGEGSLQYRIPVQPSIDQNTVDAQIEQANYAENAMHFQASFTF 120
                 ************************************************************

PAO1             LNSKFKGLVSALRGE 135
148              LNSKFKGLVSALRGE 135
ID4365           LNSKFKGLVSALRGE 135
M10              LNSKFKGLVSALRGE 135
IGB83            LNSKFKGLVSALRGE 135
```

```
                    ***************
```

flgC

```
PAO1                MSLASVFNIAGSGMSAQSTRLNTVASNIANAETVSSSVDKTYRARHPVFSTMFQQAQGDS 60
148                 MSLASVFNIAGSGMSAQSTRLNTVASNIANAETVSSSVDKTYRARHPVFSTMFQQAQGDS 60
ID4365              MSLASVFNIAGSGMSAQSTRLNTVASNIANAETVSSSVDKTYRARHPVFSTMFQQAQGDS 60
M10                 MSLASVFNIAGSGMSAQSTRLNTVASNIANAETVSSSVDKTYRARHPVFSTMFQQAQGDS 60
IGB83               MSLASVFNIAGSGMSAQSTRLNTVASNIANAETVSSSVDKTYRARHPVFSTMFQQAQGDS 60
                    ************************************************************

PAO1                GSLFADQDSSGAGVQVLGIVEDQSSLMPRYEPNHPAADANGYVYYPNVNVVEEMADMISA 120
148                 GSLFADQDSSGAGVQVLGIVEDQSSLMPRYEPNHPAADANGYVYYPNVNVVEEMADMISA 120
ID4365              GSLFADQDSSGAGVQVLGIVEDQSSLMPRYEPNHPAADANGYVYYPNVNVVEEMADMISA 120
M10                 GSLFADQDSSGAGVQVLGIVEDQSSLMPRYEPNHPAADANGYVYYPNVNVVEEMADMISA 120
IGB83               GSLFADQDSSGAGVQVLGIVEDQSSLMPRYEPNHPAADANGYVYYPNVNVVEEMADMISA 120
                    ************************************************************

PAO1                SRAFQTNAEMMNTAKQMMQKVLTLGQ 146
148                 SRAFQTNAEMMNTAKQMMQKVLTLGQ 146
ID4365              SRAFQTNAEMMNTAKQMMQKVLTLGQ 146
M10                 SRAFQTNAEMMNTAKQMMQKVLTLGQ 146
IGB83               SRAFQTNAEMMNTAKQMMQKVLTLGQ 146
                    **************************
```

flgD

```
PAO1                MSIDNVSGTSSNTGNVNGSKRAAGSGATETGQSVKGSSNLGKDEFLKLLVAQLKNQDPMS 60
148                 MSIDNVSGTSSNTGNVNGSKRAAGSGATETGQSVKGSSNLGKDEFLKLLVAQLKNQDPMS 60
IGB83               MSIDNVSGTSSNTGNVNGSKRAAGSGATETGQSVKGSSNLGKDEFLKLLVAQLKNQDPMS 60
M10                 MSIDNVSGTSSNTGNVNGSKRAAGSGATETGQSVKGSSNLGKDEFLKLLVAQLKNQDPMS 60
ID4365              MSIDNVSGTSSNTGNVNGSNRAAGSGATETGQSVKGSSNLGKDEFLKLLVAQLKNQDPMS 60
                    *******************:****************************************

PAO1                PQQNGEFIAQLAQFSTVEGVQSLNKSMESILSNYQSSQALQASSLVGRKVIVATDKSVVD 120
148                 PQQNGEFIAQLAQFSTVEGVQSLNKSMESILSNYQSSQALQASSLVGRKVIVATDKSVVD 120
IGB83               PQQNGEFIAQLAQFSTVEGVQSLNKSMESILSNYQSSQALQASSLVGRKVIVATDKSVVD 120
M10                 PQQNGEFIAQLAQFSTVEGVQSLNKSMESILSNYQSSQALQASSLVGRKVIVATDKSVVD 120
ID4365              PQQNGEFIAQLAQFSTVEGVQSLNKSMESILSNYQSSQALQASSLVGRKVIVATDKSVVD 120
                    ************************************************************

PAO1                TKDTFKASLNLPVSSSNVWVNVYDDKGTVVNRINLGQQAAGSVSFMWDGKDSSGNIMPPG 180
148                 TKDTFKASLNLPVSSSNVWVNVYDDKGTVVNRINLGQQAAGSVSFMWDGKDSSGNIMPPG 180
IGB83               TKDTFKASLNLPVSSSNVWVNVYDDKGTVVNRINLGQQAAGSVSFMWDGKDSSGNIMPPG 180
M10                 TKDTFKASLNLPVSSSNVWVNVYDDKGTVVNRINLGQQAAGSVSFMWDGKDSSGNIMPPG 180
ID4365              TKDTFKASLNLPVSSSNVWVNVYDDKGTVVNRINLGQQAAGSVSFMWDGKDSSGNIMPPG 180
                    ************************************************************

PAO1                TYKFEAQTSIDGKTYGLQTYLPANVDSVTLGQNGGELMLNLAGLGSIALSKVQIIGQ 237
148                 TYKFEAQTSIDGKTYGLQTYLPANVDSVTLGQNGGELMLNLAGLGSIALSKVQIIGQ 237
IGB83               TYKFEAQTSIDGKTYGLQTYLPANVDSVTLGQNGGELMLNLAGLGSIALSKVQIIGQ 237
M10                 TYKFEAQTSIDGKTYGLQTYLPANVDSVTLGQNGGELMLNLAGLGSIALSKVQIIGQ 237
ID4365              TYKFEAQTSIDGKTYGLQTYLPANVDSVTLGQNGGELMLNLAGLGSIALSKVQIIGQ 237
                    *********************************************************
```

flgE

```
IGB83               MSFNIGLSGIQAASSGLNVTGNNIANAGTVGFKQSRAEFADVYAASVLGSGSNPQGSGVL 60
M10                 MSFNIGLSGIQAASSGLNVTGNNIANAGTVGFKQSRAEFADVYAASVLGSGSNPQGSGVL 60
PAO1                MSFNIGLSGIQAASSGLNVTGNNIANAGTVGFKQSRAEFADVYAASVLGSGSNPQGSGVL 60
148                 MSFNIGLSGIQAASSGLNVTGNNIANAGTVGFKQSRAEFADVYAASVLGSGSNPQGSGVL 60
ID4365              MSFNIGLSGIQAASSGLNVTGNNIANAGTVGFKQSRAEFADVYAASVLGSGSNPQGSGVL 60
                    ************************************************************

IGB83               LSDVSQMFKQGNIDSTNSVLDLAINGNGFFVTSNNGAISYTRAGYFNTDKQDFIVDNNGY 120
M10                 LSDVSQMFKQGNIDSTNSVLDLAINGNGFFVTSNNGAISYTRAGYFNTDKQDFIVDNNGY 120
PAO1                LSDVSQMFKQGNIDSTNSVLDLAINGNGFFVTSNNGAISYTRAGYFNTDKQDFIVDNNGY 120
148                 LSDVSQMFKQGNIDSTNSVLDLAINGNGFFVTSNNGAISYTRAGYFNTDKQDFIVDNNGY 120
ID4365              LSDVSQMFKQGNIDSTNSVLDLAINGNGFFVTSNNGAISYTRAGYFNTDKQDFIVDNNGY 120
                    ************************************************************

IGB83               RLQGYAVGPNGQLQNGVVTDLKVERANQAPQATSSIQQSYNLNSTLKPPTVTPFDPSDAA 180
M10                 RLQGYAVGPNGQLQNGVVTDLKVERANQAPQATSSIQQSYNLNSTLKPPTVTPFDPSDAA 180
PAO1                RLQGYAVGPNGQLQNGVVTDLKVERANQAPQATSSIQQSYNLNSTLKPPTVTPFDPSDAA 180
148                 RLQGYAVGPNGQLQNGVVTDLKVERANQAPQATSSIQQSYNLNSTLKPPTVTPFDPSDAA 180
ID4365              RLQGYAVGPNGQLQNGVVTDLKVERANQAPQATSSIQQSYNLNSTLKPPTVTPFDPSDAA 180
                    ************************************************************

IGB83               TYNSSSSLGIYDSQGNSHTMSQFFIKNEPDPNATPPIPENSWTMKVLIDGVNPLDPSNKT 240
M10                 TYNSSSSLGIYDSQGNSHTMSQFFIKNEPDPNATPPIPENSWTMKVLIDGVNPLDPSNKT 240
PAO1                TYNSSSSLGIYDSQGNSHTMSQFFIKNEPDPNATPPIPENSWTMKVLIDGVNPLDPSNKT 240
148                 TYNSSSSLGIYDSQGNSHTMSQFFIKNEPDPNATPPIPENSWTMKVLIDGVNPLDPSNKT 240
ID4365              TYNSSSSLGIYDSQGNSHTMSQFFIKNEPDPNATPPIPENSWTMKVLIDGVNPLDPSNKT 240
```

```
                ************************************************************

IGB83           PMSFNVTFDASGQMTSVRAPDGSTSGPGFSIDATTNVIQFSPATGNPPTPGTGWIPAASD 300
M10             PMSFNVTFDASGQMTSVRAPDGSTSGPGFSIDATTNVIQFSPATGNPPTPGTGWIPAASD 300
PAO1            PMSFNVTFDASGQMTSVRAPDGSTSGPGFSIDATTNVIQFSPATGNPPTPGTGWIPAASD 300
148             PMSFNVTFDASGQMTSVRAPDGSTSGPGFSIDATTNVIQFSPATGNPPTPGTGWIPAASD 300
ID4365          PMSFNVTFDASGQMTSVRAPDGSTSGPGFSIDATTNVIQFSPATGNPPTPGTGWIPAASD 300
                ************************************************************

IGB83           GKTPPTYAWNGATGAASGISFDMRKTTQYSTAFAQSNPIQDGYTTGQLAGLEIDDTGVIF 360
M10             GKTPPTYAWNGATGAASGISFDMRKTTQYSTAFAQSNPIQDGYTTGQLAGLEIDDTGVIF 360
PAO1            GKTPPTYAWNGATGAASGISFDMRKTTQYSTAFAQSNPIQDGYTTGQLAGLEIDDTGVIF 360
148             GKTPPTYAWNGATGAASGISFDMRKTTQYSTAFAQSNPIQDGYTTGQLAGLEIDDTGVIF 360
ID4365          GKTPPTYAWNGATGAASGISFDMRKTTQYSTAFAQSNPIQDGYTTGQLAGLEIDDTGVIF 360
                ************************************************************

IGB83           ARYTNGQSKVQGQVVLANFANIQGLTPIGKTSWVQSSESGEPAVGAPRSGTLGALQSGAL 420
M10             ARYTNGQSKVQGQVVLANFANIQGLTPIGKTSWVQSSESGEPAVGAPRSGTLGALQSGAL 420
PAO1            ARYTNGQSKVQGQVVLANFANIQGLTPIGKTSWVQSSESGEPAVGAPRSGTLGALQSGAL 420
148             ARYTNGQSKVQGQVVLANFANIQGLTPIGKTSWVQSAESGEPAVGAPRSGTLGALQSGAL 420
ID4365          ARYTNGQSKVQGQVVLANFANIQGLTPIGKTSWVQSAESGEPAVGAPRSGTLGALQSGAL 420
                ************************************:***********************

IGB83           EASNVDISNELVNLIVHQRNYQANAKTIQTEDAVTQTIINLR 462
M10             EASNVDISNELVNLIVHQRNYQANAKTIQTEDAVTQTIINLR 462
PAO1            EASNVDISNELVNLIVHQRNYQANAKTIQTEDAVTQTIINLR 462
148             EASNVDISNELVNLIVHQRNYQANAKTIQTEDAVTQTIINLR 462
ID4365          EASNVDISNELVNLIVHQRNYQANAKTIQTEDAVTQTIINLR 462
                ******************************************
```

flgF

```
PAO1            MDKMLYVSMSGASQNTLAMRAHANNLANISTSGFRRDFEQARSMQVFGDSFPARVFAMSE 60
148             MDKMLYVSMSGASQNTLAMRAHANNLANISTSGFRRDFEQARSMQVFGDSFPARVFAMSE 60
ID4365          MDKMLYVSMSGASQNTLAMRAHANNLANISTSGFRRDFEQARSMQVFGDSFPARVFAMSE 60
M10             MDKMLYVSMSGASQNTLAMRAHANNLANISTSGFRRDFEQARSMQVFGDSFPARVFAMSE 60
IGB83           MDKMLYVSMSGASQNTLAMRAHANNLANISTSGFRRDFEQARSMQVFGDSFPARVFAMSE 60
                ************************************************************

PAO1            RPGTDFSHGSLQETGNELDIAIDGDGFVAVQAPDGSEAYVRTAGMHIDALGMLRTGDGLP 120
148             RPGTDFSHGSLQETGNELDIAIDGDGFVAVQAPDGSEAYVRTAGMHIDALGMLRTGDGLP 120
ID4365          RPGTDFSHGSLQETGNELDIAIDGDGFVAVQAPDGSEAYVRTAGMHIDALGMLRTGDGLP 120
M10             RPGTDFSHGSLQETGNELDIAIDGDGFVAVQAPDGSEAYVRTAGMHIDALGMLRTGDGLP 120
IGB83           RPGTDFSHGSLQETGNELDIAIDGDGFVAVQAPDGSEAYVRTAGMHIDALGMLRTGDGLP 120
                ************************************************************

PAO1            VLGNGGPIAVPPEEKVEIGQDGTISIRALGENPNVVAVVDRIKLVNPNLKQMEKGTDGLL 180
148             VLGNGGPIAVPPEEKVEIGQDGTISIRALGENPNVVAVVDRIKLVNPNLKQMEKGTDGLL 180
ID4365          VLGNGGPIAVPPEEKVEIGQDGTISIRALGENPNVVAVVDRIKLVNPNLKQMEKGTDGLL 180
M10             VLGNGGPIAVPPEEKVEIGQDGTISIRALGENPNVVAVVDRIKLVNPNLKQMEKGTDGLL 180
IGB83           VLGNGGPIAVPPEEKVEIGQDGTISIRALGENPNVVAVVDRIKLVNPNLKQMEKGTDGLL 180
                ************************************************************

PAO1            HYKPQPGEQAPLADANVKVVSGFLESSNVNAVEEMTAILSLSRQFELHVKMMRTAEDDSA 240
148             HYKPQPGEQAPLADANVKVVSGFLESSNVNAVEEMTAILSLSRQFELHVKMMRTAEDDSA 240
ID4365          HYKPQPGEQAPLADANVKVVSGFLESSNVNAVEEMTAILSLSRQFELHVKMMRTAEDDSA 240
M10             HYKPQPGEQAPLADANVKVVSGFLESSNVNAVEEMTAILSLSRQFELHVKMMRTAEDDSA 240
IGB83           HYKPQPGEQAPLADANVKVVSGFLESSNVNAVEEMTAILSLSRQFELHVKMMRTAEDDSA 240
                ************************************************************

PAO1            AMARVLQIS 249
148             AMARVLQIS 249
ID4365          AMARVLQIS 249
M10             AMARVLQIS 249
IGB83           AMARVLQIS 249
                *********
```

flgG

```
PAO1            MLSALWVSKTGLSAQDMNLTTISNNLANVSTTGFKRDRAEFQDLLYQIRRQPGGQSTQDS 60
148             MLSALWVSKTGLSAQDMNLTTISNNLANVSTTGFKRDRAEFQDLLYQIRRQPGGQSTQDS 60
ID4365          MLSALWVSKTGLSAQDMNLTTISNNLANVSTTGFKRDRAEFQDLLYQIRRQPGGQSTQDS 60
M10             MLSALWVSKTGLSAQDMNLTTISNNLANVSTTGFKRDRAEFQDLLYQIRRQPGGQSTQDS 60
IGB83           MLSALWVSKTGLSAQDMNLTTISNNLANVSTTGFKRDRAEFQDLLYQIRRQPGGQSTQDS 60
                ************************************************************

PAO1            ELPSGLQLGTGVRVVGTQKIFTPGSLQTTEQPLDMAVNGRGFFQVLLPDGTVSYTRDGSF 120
148             ELPSGLQLGTGVRVVGTQKIFTPGSLQTTEQPLDMAVNGRGFFQVLLPDGTVSYTRDGSF 120
ID4365          ELPSGLQLGTGVRVVGTQKIFTPGSLQTTEQPLDMAVNGRGFFQVLLPDGTVSYTRDGSF 120
M10             ELPSGLQLGTGVRVVGTQKIFTPGSLQTTEQPLDMAVNGRGFFQVLLPDGTVSYTRDGSF 120
IGB83           ELPSGLQLGTGVRVVGTQKIFTPGSLQTTEQPLDMAVNGRGFFQVLLPDGTVSYTRDGSF 120
                ************************************************************
```

```
PAO1            HLNSDGQIVTSNGFALEPAIVVPNETQTFTVGQDGTVSVTTTGNAQPQVIGNIQTADFIN 180
148             HLNSDGQIVTSNGFALEPAIVVPNETQTFTVGQDGTVSVTTTGNAQPQVIGNIQTADFIN 180
ID4365          HLNSDGQIVTSNGFALEPAIVVPNETQTFTVGQDGTVSVTTTGNAQPQVIGNIQTADFIN 180
M10             HLNSDGQIVTSNGFALEPAIVVPNETQTFTVGQDGTVSVTTTGNAQPQVIGNIQTADFIN 180
IGB83           HLNSDGQIVTSNGFALEPAIVVPNETQTFTVGQDGTVSVTTTGNAQPQVIGNIQTADFIN 180
                ************************************************************

PAO1            PAGLQAIGNNLFLETGSSGAPQVGTPGLNGLGTVAQNTLENSNVNVVEELVNMITTQRAY 240
148             PAGLQAIGNNLFLETGSSGAPQVGTPGLNGLGTVAQNTLENSNVNVVEELVNMITTQRAY 240
ID4365          PAGLQAIGNNLFLETGSSGAPQVGTPGLNGLGTVAQNTLENSNVNVVEELVNMITTQRAY 240
M10             PAGLQAIGNNLFLETGSSGAPQVGTPGLNGLGTVAQNTLENSNVNVVEELVNMITTQRAY 240
IGB83           PAGLQAIGNNLFLETGSSGAPQVGTPGLNGLGTVAQNTLENSNVNVVEELVNMITTQRAY 240
                ************************************************************

PAO1            EMNSKVISTADQMLSFVTQNL 261
148             EMNSKVISTADQMLSFVTQNL 261
ID4365          EMNSKVISTADQMLSFVTQNL 261
M10             EMNSKVISTADQMLSFVTQNL 261
IGB83           EMNSKVISTADQMLSFVTQNL 261
                *********************
```

flgH

```
PAO1            MNRLMIVSLLGIATALGGCVNPPPKPNDPYYAPVLPRTPLPAAQNNGAIYQAGFEQNLYD 60
148             MNRLMIVSLLGIATALGGCVNPPPKPNDPYYAPVLPRTPLPAAQNNGAIYQAGFEQNLYD 60
ID4365          MNRLMIVSLLGIATALGGCVNPPPKPNDPYYAPVLPRTPLPAAQNNGAIYQAGFEQNLYD 60
M10             MNRLMIVSLLGIATALGGCVNPPPKPNDPYYAPVLPRTPLPAAQNNGAIYQAGFEQNLYD 60
IGB83           MNRLMIVSLLGIATALGGCVNPPPKPNDPYYAPVLPRTPLPAAQNNGAIYQAGFEQNLYD 60
                ************************************************************

PAO1            DRKAFRVGDIITITLNEKTQASKKANSDIQKDSKTKMGLTSLFGSGMTTNNPIGGGDLSL 120
148             DRKAFRVGDIITITLNEKTQASKKANSDIQKDSKTKMGLTSLFGSGMTTNNPIGGGDLSL 120
ID4365          DRKAFRVGDIITITLNEKTQASKKANSDIQKDSKTKMGLTSLFGSGMTTNNPIGGGDLSL 120
M10             DRKAFRVGDIITITLNEKTQASKKANSDIQKDSKTKMGLTSLFGSGMTTNNPIGGGDLSL 120
IGB83           DRKAFRVGDIITITLNEKTQASKKANSDIQKDSKTKMGLTSLFGSGMTTNNPIGGGDLSL 120
                ************************************************************

PAO1            SAEYGGSRDAKGDSQAGQSNSLTGSITVTVAEVLPNGILSVRGEKWMTLNTGNELVRIAG 180
148             SAEYGGSRDAKGDSQAGQSNSLTGSITVTVAEVLPNGILSVRGEKWMTLNTGNELVRIAG 180
ID4365          SAEYGGSRDAKGDSQAGQSNSLTGSITVTVAEVLPNGILSVRGEKWMTLNTGNELVRIAG 180
M10             SAEYGGSRDAKGDSQAGQSNSLTGSITVTVAEVLPNGILSVRGEKWMTLNTGNELVRIAG 180
IGB83           SAEYGGSRDAKGDSQAGQSNSLTGSITVTVAEVLPNGILSVRGEKWMTLNTGNELVRIAG 180
                ************************************************************

PAO1            LVRADDIATDNTVSSTRVADARITYSGTGAFADASQPGWLDRFFLSPLWPF 231
148             LVRADDIATDNTVSSTRVADARITYSGTGAFADASQPGWLDRFFLSPLWPF 231
ID4365          LVRADDIATDNTVSSTRVADARITYSGTGAFADASQPGWLDRFFLSPLWPF 231
M10             LVRADDIATDNTVSSTRVADARITYSGTGAFADASQPGWLDRFFLSPLWPF 231
IGB83           LVRADDIATDNTVSSTRVADARITYSGTGAFADASQPGWLDRFFLSPLWPF 231
                ***************************************************
```

flgI

```
PAO1            MTKFKHLLALAALLLAAGAAQAERLKDIASIQGVRTNQLIGYGLVVGLSGSGDQTTQTPF 60
148             MTKFKHLLALAALLLAAGAAQAERLKDIASIQGVRTNQLIGYGLVVGLSGSGDQTTQTPF 60
ID4365          MTKFKHLLALAALLLAAGAAQAERLKDIASIQGVRTNQLIGYGLVVGLSGSGDQTTQTPF 60
M10             MTKFKHLLALAALLLAAGAAQAERLKDIASIQGVRTNQLIGYGLVVGLSGSGDQTTQTPF 60
IGB83           MTKFKHLLALAALLLAAGAAQAERLKDIASIQGVRTNQLIGYGLVVGLSGSGDQTTQTPF 60
                ************************************************************

PAO1            TLQTFNNMLAQFGIKVPANVGNVQLKNVAAVSVHADLPPFAKPGQPIDVTVSSIGNAKSL 120
148             TLQTFNNMLAQFGIKVPANVGNVQLKNVAAVSVHADLPPFAKPGQPIDVTVSSIGNAKSL 120
ID4365          TLQTFNNMLAQFGIKVPANVGNVQLKNVAAVSVHADLPPFAKPGQPIDVTVSSIGNAKSL 120
M10             TLQTFNNMLAQFGIKVPANVGNVQLKNVAAVSVHADLPPFAKPGQPIDVTVSSIGNAKSL 120
IGB83           TLQTFNNMLAQFGIKVPANVGNVQLKNVAAVSVHADLPPFAKPGQPIDVTVSSIGNAKSL 120
                ************************************************************

PAO1            RGGSLLMTPLKGIDGQVYAVAQGNLVVGGFDAEGRDGSKITVNVPSAGRIPAGATVERAV 180
148             RGGSLLMTPLKGIDGQVYAVAQGNLVVGGFDAEGRDGSKITVNVPSAGRIPAGATVERAV 180
ID4365          RGGSLLMTPLKGIDGQVYAVAQGNLVVGGFDAEGRDGSKITVNVPSAGRIPAGATVERAV 180
M10             RGGSLLMTPLKGIDGQVYAVAQGNLVVGGFDAEGRDGSKITVNVPSAGRIPAGATVERAV 180
IGB83           RGGSLLMTPLKGIDGQVYAVAQGNLVVGGFDAEGRDGSKITVNVPSAGRIPAGATVERAV 180
                ************************************************************

PAO1            PSGFDQGNSLTLNLNRPDFTTAKRIVDRINELLGPGVAHAVDGGSVRVSAPLDPNQRVDY 240
148             PSGFDQGNSLTLNLNRPDFTTAKRIVDRINELLGPGVAHAVDGGSVRVSAPLDPNQRVDY 240
ID4365          PSGFDQGNSLTLNLNRPDFTTAKRIVDRINELLGPGVAHAVDGGSVRVSAPLDPNQRVDY 240
M10             PSGFDQGNSLTLNLNRPDFTTAKRIVDRINELLGPGVAHAVDGGSVRVSAPLDPNQRVDY 240
IGB83           PSGFDQGNSLTLNLNRPDFTTAKRIVDRINELLGPGVAHAVDGGSVRVSAPLDPNQRVDY 240
                ************************************************************

PAO1            LSILENLDVQPGEAVAKVIINSRTGTIVIGQNVKVSPAAVTHGSLTVSITEDPIVSQPGA 300
148             LSILENLDVQPGEAVAKVIINSRTGTIVIGQNVKVSPAAVTHGSLTVSITEDPIVSQPGA 300
```

```
ID4365          LSILENLDVQPGEAVAKVIINSRTGTIVIGQNVKVSPAAVTHGSLTVSITEDPIVSQPGA 300
M10             LSILENLDVQPGEAVAKVIINSRTGTIVIGQNVKVSPAAVTHGSLTVSITEDPIVSQPGA 300
IGB83           LSILENLDVQPGEAVAKVIINSRTGTIVIGQNVKVSPAAVTHGSLTVSITEDPIVSQPGA 300
                ************************************************************

PAO1            FSNGQTAVVPRSRVNAEEETKPMFKFGPGTTLDDIVRAVNQVGAAPSDLMAILEALKQAG 360
148             FSNGQTAVVPRSRVNAEEETKPMFKFGPGTTLDDIVRAVNQVGAAPSDLMAILEALKQAG 360
ID4365          FSNGQTAVVPRSRVNAEEETKPMFKFGPGTTLDDIVRAVNQVGAAPSDLMAILEALKQAG 360
M10             FSNGQTAVVPRSRVNAEEETKPMFKFGPGTTLDDIVRAVNQVGAAPSDLMAILEALKQAG 360
IGB83           FSNGQTAVVPRSRVNAEEETKPMFKFGPGTTLDDIVRAVNQVGAAPSDLMAILEALKQAG 360
                ************************************************************

PAO1            ALQADLIVI 369
148             ALQADLIVI 369
ID4365          ALQADLIVI 369
M10             ALQADLIVI 369
IGB83           ALQADLIVI 369
                *********
```

flgJ

```
PAO1            MDSRLLSGIGAGPDSGSYTDLNRLNQLKVGKDRDGEANIRKVAQEFESLFLNEMLKSMRS 60
M10             MDSRLLSGIGAGPDSGSYTDLNRLNQLKVGKDRDGEANIRKVAQEFESLFLNEMLKSMRS 60
148             MDSRLLSGIGAGPDSGSYTDLNRLNQLKVGKDRDGEANIRKVAQEFESLFLNEMLKSMRS 60
ID4365          MDSRLLSGIGAGPDSGSYTDLNRLNQLKVGKDRDGEANIRKVAQEFESLFLNEMLKSMRS 60
IGB83           MDSRLLSGIGAGPDSGSYTDLNRLNQLKVGKDRDGEANIRKVAQEFESLFLNEMLKSMRS 60
                ************************************************************

PAO1            ANEALGDGNFMNSQTTKQYQDMYDQQLSVSLSKNAGGIGLADVLVRQLSKMKQGSRGNGE 120
M10             ANEALGDGNFMNSQTTKQYQDMYDQQLSVSLSKNAGGIGLADVLVRQLSKMKQGSRGNGE 120
148             ANEALGDGNFMNSQTTKQYQDMYDQQLSVSLSKNAGGIGLADVLVRQLSKMKQGSRGNGE 120
ID4365          ANEALGDGNFMNSQTTKQYQDMYDQQLSVSLSKNAGGIGLADVLVRQLSKMKQGSRGNGE 120
IGB83           ANEALGDGNFMNSQTTKQYQDMYDQQLSVSLSKNAGGIGLADVLVRQLSKMKQGSRGNGE 120
                ************************************************************

PAO1            NPFARVAENGAGRWPSNPSAQAGKALPMPEAGRDDSKLLNQRRLALPGKLAERMLAGIVP 180
M10             NPFARVAENGAGRWPSNPSAQAGKALPMPEAGRDDSKLLNQRRLALPGKLAERMLAGIVP 180
148             NPFARVAENGAGRWPSNPSAQAGKALPMPEAGRDDSKLLNQRRLALPGKLAERMLAGIVP 180
ID4365          NPFARVAENGAGRWPSNPSAQAGKALPMPEAGRDDSKLLNQRRLALPGKLAERMLAGIVP 180
IGB83           NPFARVAENGAGRWPSNPSAQAGKALPMPEAGRDDSKLLNQRRLALPGKLAERMLAGIVP 180
                ************************************************************

PAO1            SASPAASQMQSLGQDSYLPAQSYPAASRRGFSTDGVDSQGSRRIAQPPLARGKSMFASAD 240
M10             SASPAASQMQSLGQDSYLPAQSYPAASRRGFSTDGVDSQGSRRIAQPPLARGKSMFASAD 240
148             SASPAASQMQSLGQDSYLPAQSYPAASRRGFSTDGVDSQGSRRIAQPPLARGKSMFASAD 240
ID4365          SASPAASQMQSLGQDSYLPAQSYPTASRRGFSTDGVDSQGSRRIAQPPLARGKSMFASAD 240
IGB83           SASPAASQMQSLGQDSYLPAQSYPTASRRGFSTDGVDSQGSRRIAQPPLARGKSMFASAD 240
                ************************:***********************************

PAO1            EFIATMLPMAQKAAERIGVDARYLVAQAALETGWGKSIIRQQDGGSSHNLFGIKTGSRWD 300
M10             EFIATMLPMAQKAAERIGVDARYLVAQAALETGWGKSIIRQQDGGSSHNLFGIKTGSRWD 300
148             EFIATMLPMAQKAAERIGVDARYLVAQAALETGWGKSIIRQQDGGSSHNLFGIKTGSRWD 300
ID4365          EFIATMLPMAQKAAERIGVDARYLVAQAALETGWGKSIIRQQDGGSSHNLFGIKTGSRWD 300
IGB83           EFIATMLPMAQKAAERIGVDARYLVAQAALETGWGKSIIRQQDGGSSHNLFGIKTGSRWD 300
                ************************************************************

PAO1            GASARALTTEYEGGKAVKEIAAFRSYSSFEQSFHDYVSFLQGNDRYQNALDSAANPERFM 360
M10             GASARALTTEYEGGKAVKEIAAFRSYSSFEQSFHDYVSFLQGNDRYQNALDSAANPERFM 360
148             GASARALTTEYEGGKAVKEVAAFRSYSSFEQSFHDYVSFLQGNDRYQNALDSAANPERFM 360
ID4365          GASARALTTEYEGGKAVKEVAAFRSYSSFEQSFHDYVSFLQGNDRYQNALDSAANPERFM 360
IGB83           GASARALTTEYEGGKAVKEVAAFRSYSSFEQSFHDYVSFLQGNDRYQNALDSAANPERFM 360
                *******************:****************************************

PAO1            QELQRAGYATDPQYARKVAQIARQMQTYQAVAAAGTPPLG 400
M10             QELQRAGYATDPQYARKVAQIARQMQTYQAVAAAGTPPLG 400
148             QELQRAGYATDPQYARKVAQIARQMQTYQAVAAAGTPPLG 400
ID4365          QELQRAGYATDPQYARKVAQIARQMQTYQAVAAAGTPPLG 400
IGB83           QELQRAGYATDPQYARKVAQIARQMQTYQAVAAAGTPPLG 400
                ****************************************
```

flgK

```
PAO1            MSDLLSIGLSGLGTSQTWLTVTGHNITNVKTPGYSRQDAIQQTRIPQFSGAGYMGSGSQI 60
M10             MSDLLSIGLSGLGTSQTWLTVTGHNITNVKTPGYSRQDAIQQTRIPQFSGAGYMGSGSQI 60
IGB83           MSDLLSIGLSGLGTSQTWLTVTGHNITNVKTPGYSRQDAIQQTQVPQFSGAGYMGSGSQI 60
ID4365          MSDLLSIGLSGLGTSQTWLTITGHNITNVKTPGYSRQDAIQQTRIPQFSGAGYMGSGSQI 60
148             MSDLLSIGLSGLGTSQTWLTITGHNITNVKTPGYSRQDAIQQTQVPQFSGAGYMGSGSQI 60
                ********************:**********************::***************

PAO1            VDVRRLASDFLTGQLRNATSQNSELNAFLGQIDQLNSLLADNTTGVSPAMQRFFSALQTA 120
M10             VDVRRLASDFLTGQLRNATSQNSELNAFLGQIDQLNSLLADNTTGVSPAMQRFFSALQTA 120
IGB83           VDVRRLASDFLTGQLRNATSQNSELNAFLGQIDQLNSLLADNTTGVSPAMQRFFSALQTA 120
ID4365          VDVRRLASDFLTGQLRNATSQNSELNAFLGQIDQLNSLLADNTTGVSPAMQRFFSALQTA 120
```

```
148              VDVRRLASDFLTGQLRNATSQNSELSAFRSQIEQLDGLLSNTTTGVSPAMQRFFAALQAA 120
                 ************************.** .**:**:.**::.************:***:*

PAO1             AQNPSSTEAREAVLAQAQGLSKTFNTLYDQLDKQNSLINQQLGALTSQVNNLSQSVAEYN 180
M10              AQNPSSTEAREAVLAQAQGLSKTFNTLYDQLDKQNSLINQQLGALTSQVNNLSQSVAEYN 180
IGB83            AQNPSSTEAREAVLAQAQGLSKTFNTLYDQLDKQNSLINQQLGALTSQVNNLSQSVAEYN 180
ID4365           AQNPSSTEAREAVLAQAQGLSKTFNTLYDQLDKQNSLINQQLGALTSQVNNLSQSVAEYN 180
148              ANNPSSTEAREAVLAQAEGLGKTFNTLYDQLDKQNSLINQQLGALASQVNHLSQSVASYN 180
                 *:***************:**.************************:****:******.**

PAO1             DAIAKAKSAGAVPNDLLDARDEAVRKLSEMVGVTAVTQDDNSVSLFIGSGQPLVVGNTVS 240
M10              DAIAKAKSAGAVPNDLLDARDEAVRKLSEMVGVTAVTQDDNSVSLFIGSGQPLVVGNTVS 240
IGB83            DAIAKAKSAGAVPNDLLDARDEAVRKLSEMVGVTAVTQDDNSVSLFIGSGQPLVVGNTVS 240
ID4365           DAIAKAKSAGAVPNDLLDARDEAVRKLSEMVGVTAVTQDDNSVSLFIGSGQPLVVGNTVS 240
148              DAIAKAKSAGAVPNDLMDARDEAVRKLSEMIGVTAVTQDDNSVSLFIGSGQPLVVGNTVS 240
                 ****************:*************:*****************************

PAO1             TLSVVPGLDDPTRYQVQLTLGDSTQNVTRLVSGGQMGGLLAYRDTVLDSSYNKLGQLALT 300
M10              TLSVVPGLDDPTRYQVQLTLGDSTQNVTRLVSGGQMGGLLAYRDTVLDSSYNKLGQLALT 300
IGB83            TLSVVPGLDDPTRYQVQLTLGDSTQNVTRLVSGGQMGGLLAYRDTVLDSSYNKLGQLALT 300
ID4365           TLSVVPGLDDPTRYQVQLTLGDSTQNVTRLVSGGQMGGLLAYRDTVLDSSYNKLGQLALT 300
148              TLSVVPGLDDPTRYQVQLSNGNSIQNVTGLVSGGEMGGLLAYRNSALDSSYNKLGQLAIT 300
                 ******************: *:* **** *****:********::.************:*

PAO1             FADTVNKQLGQGLDLAGKAGANLFGDINDPDITALRVLAKNGNTGNVHANLNITDTSKLN 360
M10              FADTVNKQLGQGLDLAGKAGANLFGDINDPDITALRVLAKNGNTGNVHANLNITDTSKLN 360
IGB83            FADTVNKQLGQGLDLAGKAGANLFGDINDPDITALRVLAKNGNTGNVHANLNITDTSKLN 360
ID4365           FADTVNKQLGQGLDLAGKAGANLFGDINDPDITALRVLAKNGNTGNVHANLNITDTSKLN 360
148              LADTINKQLGQGLDLAGKAGANLFGDINDPDITALRVLAKNGNTGNVHANLNITDTSKLN 360
                 :***:*******************************************************

PAO1             SSDFRLDFDGTNFTARRLGDDASMQVTVSGTGPYTLSFKDANGVDQGFSVTLDQLPAAGD 420
M10              SSDFRLDFDGTNFTARRLGDDASMQVTVSGTGPYTLSFKDANGVDQGFSVTLDQLPAAGD 420
IGB83            SSDFRLDFDGTNFTARRLGDDASMQVTVSGTGPYTLSFKDANGVDQGFSVTLDQLPAAGD 420
ID4365           SSDFRLDFDGTNFTARRLGDDASMQVTVSGTGPYTLSFKDANGVDQGFSVTLDQLPAAGD 420
148              SSDFRLDFDGTNFTARRLGDDASMQVTVSGTGPYTLSFKDANGIDQGFNLTLDQLPAAGD 420
                 *******************************************:****.:**********

PAO1             RFTLQPTRRGASDIETTLKNASQLAFAGSARAEATTNNRGSGAIGQPNLVDGPSPIDPAV 480
M10              RFTLQPTRRGASDIETTLKNASQLAFAGSARAEATTNNRGSGAIGQPNLVDGPSPIDPAV 480
IGB83            RFTLQPTRRGASDIETTLKNASQLAFAGSARAEATTNNRGSGAIGQPNLVDGPSPIDPAV 480
ID4365           RFTLQPTRRGASDIETTLKNASQLAFAGSARAEATTNNRGSGAIGQPNLVGGPSPIDPAV 480
148              RFTLQPTRRGAADIEATLKNASQLAFAGTARTESTTENRGTGKIGAPILTSGPSPVDPAV 480
                 ***********:***:************:**:*:**:***:* ** * *..****:****

PAO1             LQNAFGANGLPLSATVSADGKTYTMTSPLPAGWSYVDKDGNALPGSPTLNSGTSNSVRMA 540
M10              LQNAFGANGLPLSATVSADGKTYTMTSPLPAGWSYVDKDGNALPGSPTLNSGTSNSVRMA 540
IGB83            LQNAFGANGLPLSATVSANGKTYTMTSPLPAGWSYVDKDGNALPGSPTLNSGTSNSVRMA 540
ID4365           LQNAFGANGLPLSATVSADGKTYTMTSPLPAGWSYVDKDGNALPGSPTLNSGTSNSVRMA 540
148              LQGAFGPNGISLGATLSADGKTYTLSSPLPAGWSYVDKDGNALTGSPTLTSGNTNTVRMA 540
                 **.***.**:.*.**:**:*****::*****************.*****.**.:*:****

PAO1             YTDPGSGQTYTYEFNLSNVPQTGDSFTLSFNKDGIADNRNALNLNALQTKPTVGGTDSTG 600
M10              YTDPGSGQTYTYEFNLSNVPQTGDSFTLSFNKDGIADNRNALNLNALQTKPTVGGTDSTG 600
IGB83            YTDPGSGQTYTYEFNLSNVPQTGDSFTLSFNKDGIADNRNALNLNALQTKPTVGGTDSTG 600
ID4365           YTDPGSGQTYTYEFNLSNVPQTGDSFTLSFNKDGISDNRNALNLNALQTKPTVGGTGSTG 600
148              FTDP-SGQKYSYEFELSGVPQNSDSFKLGFNDKGISDNRNALNLLALQTKPTVGGTDNTG 599
                 :*** ***.*:***:**.***..***.*.**..**:******** ***********..**

PAO1             STYNDAYGGLVERVGTLTAQARASADASQTVLKQAQDSRDSLSGVSLDEEAANLIQFQQY 660
M10              STYNDAYGGLVERVGTLTAQARASADASQTVLKQAQDSRDSLSGVSLDEEAANLIQFQQY 660
IGB83            STYNDAYGGLVERVGTLTAQARASADASQTVLKQAQDSRDSLSGVSLDEEAANLIQFQQY 660
ID4365           STYNDAYGGLVERVGTLTAQARASADASQTVLKQAQDSRDSLSGVSLDEEAANLIQFQQY 660
148              STYNEAYGGLVERVGTLTAQVRASSEASATVLKQAQDSRDSLSGVSLDEEAANLIQFQQY 659
                 ****:***************.***::** *******************************

PAO1             YSASAQVIQVARSLFDTLIGAFR 683
M10              YSASAQVIQVARSLFDTLIGAFR 683
IGB83            YSASAQVIQVARSLFDTLIGAFR 683
ID4365           YSASAQVIQVARSLFDTLIGAFR 683
148              YGASAQVIQVARTLFDTLIGAFR 682
                 *.**********:**********
```

flgL

```
IGB83            MRISTIQAFNNSVSGISRNYADLTRTQAEISAGKRLLTPADDPVGAVRLLQLNQEQALNS 60
M10              MRISTIQAFNNSVSGISRNYADLTRTQAEISAGKRLLTPADDPVGAVRLLQLNQEQALNS 60
PAO1             MRISTIQAFNNSVSGISRNYADLTRTQAEISAGKRLLTPADDPVGAVRLLQLNQEQALNS 60
ID4365           MRISTIQAFNNSVSGISRNYADLTRTQAEISAGKRLLTPADDPVGAVRLLQLNQEQALNS 60
148              MRISTIQAFNNSVNGISRNYADLNRTFEQISTGKRILTPADDPVGSVRLLRLDQEQGLNE 60
                 *************.*********.**  :**:***:*********:****:*:***.**.

IGB83            QYKSGITAAKNSLQQEETILNSVGTVIHRIREIAVQAGNGGLDASDKNALATELAQREDE 120
```

```
M10             QYKSGITAAKNSLQQEETILNSVGTVIHRIREIAVQAGNGGLDASDKNALATELAQREDE 120
PAO1            QYKSGITAAKNSLQQEETILNSVGTVIHRIREIAVQAGNGGLDASDKNALATELAQREDE 120
ID4365          QYKSGITAAKNSLQQEETILNSVGTVIHRIREIAVQAGNGGLDASDKNALATELAQREDE 120
148             QYKTGMTEAKNSLSQEETILRSVGNVLQRIREIAGQAGDGALDSNDKKSLASELRQREDE 120
                ***:*:* *****.******.***.*::****** ***:*.**:.**::**:** *****

IGB83           LLNLLNSRDASGKYLFSGSQGDTQPFVRNPDGTYSYNGDEGQREVQIASSTFIAISDNGK 180
M10             LLNLLNSRDASGKYLFSGSQGDTQPFVRNPDGTYSYNGDEGQREVQIASSTFIAISDNGK 180
PAO1            LLNLLNSRDASGKYLFSGSQGDTQPFVRNPDGTYSYNGDEGQREVQIASSTFIAISDNGK 180
ID4365          LLNLLNSRDASGKYLFSGSQGDTQPFVRNPDGTYSYNGDEGQREVQIASSTFIAISDNGK 180
148             LLNLLNSRDASGKYLFSGSQGSVQPFVRNEDGTYSYMGDESQREVQIASSTRIPVSDSGK 180
                *********************..****** ****** ***.********** *.:**.**

IGB83           ILFESGSNANRVSTGKDAAGPDASGNPSDSSISLGLVTDKEAYDTVFPSSTPPLASDGVG 240
M10             ILFESGSNANRVSTGKDAAGPDASGNPSDSSISLGLVTDKEAYDTVFPSSTPPLASDGVG 240
PAO1            ILFESGSNANRVSTGKDAAGPDASGNPSDSSISLGLVTDKEAYDTVFPSSTPPLASDGVG 240
ID4365          ILFESGSNANRVSTGKDAAGLDASGNPSDSSISLGLVTDKEAYDTVFPSSTPPLASDGVG 240
148             VLFEDIVNAARLDT-KAAAG-----NTGDGRISVGLVEDELAFDSQFPASNPPAATDGFN 234
                :***.  ** *:.* * ***     *..*. **:*** *: *:*: **:*.** *:**..

IGB83           IHFTDAKNYVVYDLKTIPPGYDWSTSDPNPPSFATLASGKIDDNAQTSDFITFGGVKVQI 300
M10             IHFTDAKNYVVYDLKTIPPGYDWSTSDPNPPSFATLASGKIDDNAQTSDFITFGGVKVQI 300
PAO1            IHFTDAKNYVVYDLKTIPPGYDWSTSDPNPPSFATLASGKIDDNAQTSDFITFGGVKVQI 300
ID4365          IHFTDAKNYVVYDLKTIPPGYDWSTSDPNPPSFATLASGKIDDNAQTSDFITFGGVKVQI 300
148             IHFVSDKEYVVYDPKSLPPGYDWTTYDPNSPPAWQLSKGAIDDDPKTIDKVLYAGVSVTI 294
                ***.. *:***** *::******:* ***.*.   *:.* ***:.:* * : :.**.* *

IGB83           DGEPQGGDTFSIKRQPDQEKRSLLNTVSDLRKALLSAEDTPAGNLAIRDAVGVAISNLDS 360
M10             DGEPQGGDTFSIKRQPDQEKRSLLNTVSDLRKALLSAEDTPAGNLAIRDAVGVAISNLDS 360
PAO1            DGEPQGGDTFSIKRQPDQEKRSLLNTVSDLRKALLSAEDTPAGNLAIRDAVGVAISNLDS 360
ID4365          DGEPQGGDTFSIKRQPDQEKRSLLNTVSDLRKALLSAEDTPAGNLAIRDAVGVAISNLDS 360
148             DGTPKAGDEFNVNYKPGSEKRSLLNVVSDLRKALESSTDNQAGNDAIRDATAVALTNLSA 354
                ** *:.** *.:: :*..*******.******** *: *. *** *****..**::**.:

IGB83           SNSQILTGQGRIGARMNVAESTETFIDDVTLVNTAVISQIQDLDYPEALSRLTLQSTIMD 420
M10             SNSQILTGQGRIGARMNVAESTETFIDDVTLVNTAVISQIQDLDYPEALSRLTLQSTIMD 420
PAO1            SNSQILTGQGRIGARMNVAESTETFIDDVTLVNTAVISQIQDLDYPEALSRLTLQSTIMD 420
ID4365          SNSQILTGQGRIGARMNVAESTETFIDDVTLVNTAVISQIQDLDYPEALSRLTLQSTIMD 420
148             VAAAVDGGQGKIGARLNTVESTETFIDDVKLVNASVMSQIQDLDYAEALSRLSLQSTIMD 414
                  : :  ***:****:*..**********.***::*:********.******:*******

IGB83           AAQQSFVKIRGLSLFNYLS 439
M10             AAQQSFVKIRGLSLFNYLS 439
PAO1            AAQQSFVKIRGLSLFNYLS 439
ID4365          AAQQSFVKIRGLSLFNYLS 439
148             AAQQSYVKIQGLSLFNYLK 433
                *****:***:********.
```

flgM

```
IGB83           MVIDFNRLNPGSTPATTGRTGSTAAGRPDATGADKAGQAATSAPKSGESVQISETAQNMQ 60
M10             MVIDFNRLNPGSTPATTGRTGSTAAGRPDATGADKAGQAATSAPKSGESVQISETAQNMQ 60
ID4365          MVIDFNRLNPGSTPATTGRTGSTAAGRPDATGADKAGQAATSAPKSGESVQISETAQNMQ 60
PAO1            MVIDFNRLNPGSTPATTGRTGSTAAGRPDATGADKAGQAATSAPKSGESVQISETAQNMQ 60
148             MVIDFNRLNPGSTPATTGRTGSTAAGRPDATGADKAGPAATSAPKSGESVQISETAQNMQ 60
                ************************************* **********************

IGB83           KVTDQLQTLPVVDNDKVARIKQAIADGTYQVDSERVASKLLDFESQR 107
M10             KVTDQLQTLPVVDNDKVARIKQAIADGTYQVDSERVASKLLDFESQR 107
ID4365          KVTDQLQTLPVVDNDKVARIKQAIADGTYQVDSERVASKLLDFESQR 107
PAO1            KVTDQLQTLPVVDNDKVARIKQAIADGTYQVDSERVASKLLDFESQR 107
148             KVTDQLQTLPVVDNDKVARIKQAIADGTYQVDSERVASKLLDFESQR 107
                ***********************************************
```

fleQ

```
PAO1            MWRETKLLLIDDNLDRSRDLAVILNFLGEDQLTCNSEDWREVAAGLSNSREALCVLLGSV 60
148             MWRETKLLLIDDNLDRSRDLAVILNFLGEDQLTCNSEDWREVAAGLSNSREALCVLLGSV 60
IGB83           MWRETKLLLIDDNLDRSRDLAVILNFLGEDQLTCNSEDWREVAAGLSNSREALCVLLGSV 60
M10             MWRETKLLLIDDNLDRSRDLAVILNFLGEDQLTCNSEDWREVAAGLSNSREALCVLLGSV 60
ID4365          MWRETKLLLIDDNLDRSRDLAVILNFLGEDQLTCNSEDWREVAAGLSNSREALCVLLGSV 60
                ************************************************************

PAO1            ESKGGAVELLKQLASWDEYLPILLIGEPAPADWPEELRRRVLASLEMPPSYNKLLDSLHR 120
148             ESKGGAVELLKQLASWDEYLPILLIGEPAPADWPEELRRRVLASLEMPPSYNKLLDSLHR 120
IGB83           ESKGGAVELLKQLASWDEYLPILLIGEPAPADWPEELRRRVLASLEMPPSYNKLLDSLHR 120
M10             ESKGGAVELLKQLASWDEYLPILLIGEPAPADWPEELRRRVLASLEMPPSYNKLLDSLHR 120
ID4365          ESKGGAVELLKQLASWDEYLPILLIGEPAPADWPEELRRRVLASLEMPPSYNKLLDSLHR 120
                ************************************************************

PAO1            AQVYREMYDQARERGRSREPNLFRSLVGTSRAIQQVRQMMQQVADTDASVLILGESGTGK 180
148             AQVYREMYDQARERGRSREPNLFRSLVGTSRAIQQVRQMMQQVADTDASVLILGESGTGK 180
IGB83           AQVYREMYDQARERGRSREPNLFRSLVGTSRAIQQVRQMMQQVADTDASVLILGESGTGK 180
```

```
M10             AQVYREMYDQARERGRSREPNLFRSLVGTSRAIQQVRQMMQQVADTDASVLILGESGTGK 180
ID4365          AQVYREMYDQARERGRSREPNLFRSLVGTSRAIQQVRQMMQQVADTDASVLILGESGTGK 180
                ************************************************************

PAO1            EVVARNLHYHSKRREGPFVPVNCGAIPAELLESELFGHEKGAFTGAITSRAGRFELANGG 240
148             EVVARNLHYHSKRREGPFVPVNCGAIPAELLESELFGHEKGAFTGAITSRAGRFELANGG 240
IGB83           EVVARNLHYHSKRREGPFVPVNCGAIPAELLESELFGHEKGAFTGAITSRAGRFELANGG 240
M10             EVVARNLHYHSKRREGPFVPVNCGAIPAELLESELFGHEKGAFTGAITSRAGRFELANGG 240
ID4365          EVVARNLHYHSKRREGPFVPVNCGAIPAELLESELFGHEKGAFTGAITSRAGRFELANGG 240
                ************************************************************

PAO1            TLFLDEIGDMPLPMQVKLLRVLQERTFERVGSNKTQNVDVRIIAATHKNLEKMIEDGTFR 300
148             TLFLDEIGDMPLPMQVKLLRVLQERTFERVGSNKTQNVDVRIIAATHKNLEKMIEDGTFC 300
IGB83           TLFLDEIGDMPLPMQVKLLRVLQERTFERVGSNKTQNVDVRIIAATHKNLEKMIEDGTFR 300
M10             TLFLDEIGDMPLPMQVKLLRVLQERTFERVGSNKTQKVDVRIIAATHKNLEKMIEDGTFR 300
ID4365          TLFLDEIGDMPLPMQVKLLRVLQERTFERGGSNKTQNVDVRIIAATHKNLEKMIEDGTFR 300
                ***************************** ******:********************** 

PAO1            EDLYYRLNVFPIEMAPLRERVEDIALLLNELISRMEHEKRGSIRFNSAAIMSLCRHDWPG 360
148             EDLYYRLNVFPIEMAPLRERVEDIALLLNELISRMEHEKRGSIRFNSAAIMSLCRHDWPG 360
IGB83           EDLYYRLNVFPIEMAPLRERVEDIALLLNELISRMEHEKRGSIRFNSAAIMSLCRHDWPG 360
M10             EDLYYRLNVFPIEMAPLRERVEDIALLLNELISRMEHEKRGSIRFNSAAIMSLCRHDWPG 360
ID4365          EDLYYRLNVFPIEMAPLRERVEDIALLLNELISRMEHEKRGSIRFNSAAIMSLCRHDWPG 360
                ************************************************************

PAO1            NVRELANLVERLAIMHPYGVIGVGELPKKFRHVDDEDEQLASSLREELEERAAINAGLPG 420
148             NVRELANLVERLAIMHPYGVIGVGELPKKFRHVDDEDEQLASSLREELEERAAINAGLPG 420
IGB83           NVRELANLVERLAIMHPYGVIGVGELPKKFRHVDDEDEQLASSLREELEERAAINAGLPG 420
M10             NVRELANLVERLAIMHPYGVIGVGELPKKFRHVDDEDEQLASSLREELEERAAINAGLPG 420
ID4365          NVRELANLVERLAIMHPYGVIGVGELPKKFRHVDDEDEQLASSLREELEERAAINAGLPG 420
                ************************************************************

PAO1            MDAPAMLPAEGLDLKDYLANLEQGLIQQALDDAGGVVARAAERLRIRRTTLVEKMRKYGM 480
148             MDAPAMLPAEGLDLKDYLANLEQGLIQQALDDAGGVVARAAERLRIRRTTLVEKMRKYGM 480
IGB83           MDAPAMLPAEGLDLKDYLANLEQGLIQQALDDAGGVVARAAERLRIRRTTLVEKMRKYGM 480
M10             MDAPAMLPAEGLDLKDYLANLEQGLIQQALDDAGGVVARAAERLRIRRTTLVEKMRKYGM 480
ID4365          MDAPAMLPAEGLDLKDYLANLEQGLIQQALDDAGGVVARAAERLRIRRTTLVEKMRKYGM 480
                ************************************************************

PAO1            SRRDDDLSDD 490
148             SRRDDDLSDD 490
IGB83           SRRDDDLSDD 490
M10             SRRDDDLSDD 490
ID4365          SRRDDDLSED 490
                ********:*
```

fleR

```
PAO1            MAAKVLLVEDDRALREALSDTLLLGGHEFVAVDSAEAALPVLAREAFSLVISDVNMPGMD 60
148             MAAKVLLVEDDRALREALSDTLLLGGHEFVAVDSAEAALPVLAREAFSLVISDVNMPGMD 60
IGB83           MAAKVLLVEDDRALREALSDTLLLGGHEFVAVDSAEAALPVLAREAFSLVISDVNMPGMD 60
M10             MAAKVLLVEDDRALREALSDTLLLGGHEFVAVDSAEAALPVLAREAFSLVISDVNMPGMD 60
ID4365          MAAKVLLVEDDRALREALSDTLLLGGHEFVAVDSAEAALPVLAREAFSLVISDVNMPGMD 60
                ************************************************************

PAO1            GHQLLGLIRTRYPHLPVLLMTAYGAVDRAVEAMRQGAADYLVKPFEARALLDLVARHALG 120
148             GHQLLGLIRTRYPHLPVLLMTAYGAVDRAVEAMRQGAADYLVKPFEARALLDLVARHALG 120
IGB83           GHQLLGLIRTRYPHLPVLLMTAYGAVDRAVEAMRQGAADYLVKPFEARALLDLVARHALG 120
M10             GHQLLGLIRTRYPHLPVLLMTAYGAVDRAVEAMRQGAADYLVKPFEARALLDLVARHALG 120
ID4365          GHQLLGLIRTRYPHLPVLLMTAYGAVDRAVEAMRQGAADYLVKPFEARALLDLVARHALG 120
                ************************************************************

PAO1            QLPGSEEDGPVALEPASRQLLELAARVARSDSTVLISGESGTGKEVLANYIHQQSPRAGK 180
148             QLPGSEEDGPVALEPASRQLLELAARVARSDSTVLISGESGTGKEVLANYIHQQSPRAGK 180
IGB83           QLPGSEEDGPVALEPASRQLLELAARVARSDSTVLISGESGTGKEVLANYIHQQSPRAGK 180
M10             QLPGSEEDGPVALEPASRQLLELAARVARSDSTVLISGESGTGKEVLANYIHQQSPRAGK 180
ID4365          QLPGSEEDGPVALEPASRQLLELAARVARSDSTVLISGESGTGKEVLANYIHQQSPRAGK 180
                ************************************************************

PAO1            PFIAINCAAIPDNMLEATLFGHEKGSFTGAIAAQPGKFELADGGTILLDEISEMPLGLQA 240
148             PFIAINCAAIPDNMLEATLFGHEKGSFTGAIAAQPGKFELADGGTILLDEISEMPLGLQA 240
IGB83           PFIAINCAAIPDNMLEATLFGHEKGSFTGAIAAQPGKFELADGGTILLDEISEMPLGLQA 240
M10             PFIAINCAAIPDNMLEATLFGHEKGSFTGAIAAQPGKFELADGGTILLDEISEMPLGLQA 240
ID4365          PFIAINCAAIPDNMLEATLFGHEKGSFTGAIAAQPGKFELADGGTILLDEISEMPLGLQA 240
                ************************************************************

PAO1            KLLRVLQEREVERVGARKPINLDIRVLATTNRDLAAEVAAGRFREDLYYRLSVFPLAWRP 300
148             KLLRVLQEREVERVGARKPINLDIRVLATTNRDLAAEVAAGRFREDLYYRLSVFPLAWRP 300
IGB83           KLLRVLQEREVERVGARKPINLDIRVLATTNRDLAAEVAAGRFREDLYYRLSVFPLAWRP 300
M10             KLLRVLQEREVERVGARKPINLDIRVLATTNRDLAAEVAAGRFREDLYYRLSVFPLAWRP 300
ID4365          KLLRVLQEREVERVGARKPINLDIRVLATTNRDLAAEVAAGRFREDLYYRLSVFPLAWRP 300
                ************************************************************
```

```
PAO1            LRERPADILPLAERLLRKHSRKMNLGAVALGPEAAQCLVRHAWPGNVRELDNAIQRALIL 360
148             LRERPADILPLAERLLRKHSRKMNLGAVALGPEAAQCLVRHAWPGNVRELDNAIQRALIL 360
IGB83           LRERPADILPLAERLLRKHSRKMNLGAVALGPEAAQCLVRHAWPGNVRELDNAIQRALIL 360
M10             LRERPADILPLAERLLRKHSRKMNLGAVALGPEAAQCLVRHAWPGNVRELDNAIQRALIL 360
ID4365          LRERPADILPLAERLLRKHSRKMNLGAVALGPEAAQCLVRHAWPGNVRELDNAIQRALIL 360
                ************************************************************

PAO1            QQGGLIQPADLCLTAPIGMPLAAPVPVPMPAMPPATPPSVEIPSPAAGQDASGALGDDLR 420
148             QQGGLIQPADLCLTAPIGMPLAA--PVPMPAMPPATPPSVEIPSPAAGQDASGALGDDLR 418
IGB83           QQGGLIQPADLCLTAPIGMPLAAPVPVPMPAMPPATPPSVEIPSPAAGQDASGALGDDLR 420
M10             QQGGLIQPADLCLTAPIGMPLAAPVPVPMPAMPPATPPSVEIPSPAAGQDASGALGDDLR 420
ID4365          QQGGLIQPADLCLTAPIGMPLAAPVPVPMPAMPPATPPSVEIPSPAAGQDATGALGDDLR 420
                ***********************  **************************:********

PAO1            RREFQVIIDTLRTERGRRKEAAERLGISPRTLRYKLAQMRDAGMDVEAYLYAI 473
148             RREFQVIIDTLRTERGRRKEAAERLGISPRTLRYKLAQMRDAGMDVEAYLYAI 471
IGB83           RREFQVIIDTLRTERGRRKEAAERLGISPRTLRYKLAQMRDAGMDVEAYLYAI 473
M10             RREFQVIIDTLRTERGRRKEAAERLGISPRTLRYKLAQMRDAGMDVEAYLYAI 473
ID4365          RREFQVIIDTLRTERGRRKEAAERLGISPRTLRYKLAQMRDAGMDVEAYLYAI 473
                *****************************************************
```

fleS

```
PAO1            MQPALNAFTEQPADTAEATSRAGLEQAFALFNQMSSQLSESYSLLEERVTELKGQLALVS 60
148             MQPALNAFTEQPADTAEATSRAGLEQAFALFNQMSSQLSESYSLLEERVTELKGQLALVS 60
M10             MQPALNAFTEQPADTAEATSRAGLEQAFALFNQMSSQLSESYSLLEERVTELKGQLALVS 60
ID4365          MQPALNAFPEQPADTAEATSRAGLEQAFALFNQMSSQLSESYSLLEERVTELKGQLALVS 60
IGB83           MQPALNAFPEQPADTAEATSRAGLEQAFALFNQMSSQLSESYSLLEERVTELKGQLALVS 60
                ********.***************************************************

PAO1            AQRMEELAEKERLANRLQSLLDLLPGGVIVIDAHGVVREANPAALGLLGEPLVGMLWREV 120
148             AQRMEELAEKERLANRLQSLLDLLPGGVIVIDAHGVVREANPAALGLLGEPLVGMLWREV 120
M10             AQRMEELAEKERLANRLQSLLDLLPGGVIVIDAHGVVREANPAALGLLGEPLVGMLWREV 120
ID4365          AQRMEELAEKERLANRLQSLLDLLPGGVIVIDAHGVVREANPAALGLLGEPLVGMLWREV 120
IGB83           AQRMEELAEKERLANRLQSLLDLLPGGVIVIDAHGVVREANPAALGLLGEPLVGMLWREV 120
                ************************************************************

PAO1            IARCFAPREDDGHEISLRDGRRLSIATRSLNGEPGQLILLNDLTDTRRLQEQLARHERLS 180
148             IARCFAPREDDGHEISLRDGRRLSIATRSLNGEPGQLILLNDLTDTRRLQEQLARHERLS 180
M10             IARCFAPREDDGHEISLRDGRRLSIATRSLNGEPGQLILLNDLTDTRRLQEQLARHERLS 180
ID4365          IARCFAPREDDGHEISLRDGRRLSIATRSLNGEPGQLILLNDLTDTRRLQEQLARHERLS 180
IGB83           IARCFAPREDDGHEISLRDGRRLSIATRSLNGEPGQLILLNDLTDTRRLQEQLARHERLS 180
                ************************************************************

PAO1            ALGRMVASLAHQIRTPLSAALLYAGHLSEQALPTDQQQRFAGRLKERLHELEHQVRDMLV 240
148             ALGRMVASLAHQIRTPLSAALLYAGHLSEQALPTDQQQRFAGRLKERLHELEHQVRDMLV 240
M10             ALGRMVASLAHQIRTPLSAALLYAGHLSEQALPTDQQQRFAGRLKERLHELEHQVRDMLV 240
ID4365          ALGRMVASLAHQIRTPLSAALLYAGHLSEQALPTDQQQRFAGRLKERLHELEHQVRDMLV 240
IGB83           ALGRMVASLAHQIRTPLSAALLYAGHLSEQALPTDQQQRFAGRLKERLHELEHQVRDMLV 240
                ************************************************************

PAO1            FARGELPLTDRVAPKALFDSLRAAAEVHVQGLQVRWQCEARGGELLCNRDTLVGTVLNLV 300
148             FARGELPLTDRVAPKALFDSLRAAAEVHVQGLQVRWQCEARGGELLCNRDTLVGTVLNLV 300
M10             FARGELPLTDRVAPKALFDSLRAAAEVHVQGLQVRWQCEARGGELLCNRDTLVGTVLNLV 300
ID4365          FARGELPLTDRVAPKALFDSLRAAAEVHVQGLQVRWQCEARGGELLCNRDTLVGTVLNLV 300
IGB83           FARGELPLTDRVAPKALFDSLRAAAEVHVQGLQVRWQCEARGGELLCNRDTLVGTVLNLV 300
                ************************************************************

PAO1            ENAIQACGPELRLKVHLYARADSLRLSVSDNGPGMDPATLARLGEPFFTTKTTGTGLGLA 360
148             ENAIQACGPELRLKVHLYARADSLRLSVSDNGPGMDPATLARLGEPFFTTKTTGTGLGLA 360
M10             ENAIQACGPELRLKVHLYARADSLRLSVSDNGPGMDPATLARLGEPFFTTKTTGTGLGLA 360
ID4365          ENAIQACGPELRLKVHLYARADSLRLSVSDNGPGMDPATLARLGEPFFTTKTTGTGLGLA 360
IGB83           ENAIQACGPELRLKVHLYARADSLRLSVSDNGPGMDPATLARLGEPFFTTKTTGTGLGLA 360
                ************************************************************

PAO1            VVKAVARAHQGQLQLRSRPGRGTCATLILPLIPAAPLSAIQE 402
148             VVKAVARAHQGQLQLRSRPGRGTCATLILPLIPAAPLSAIQE 402
M10             VVKAVARAHQGQLQLRSRPGRGTCATLILPLIPAAPLSAIQE 402
ID4365          VVKAVARAHQGQLQLRSRPGRGTCATLILPLIPAAPLSAIQE 402
IGB83           VVKAVARAHQGQLQLRSRPGRGTCATLILPLIPAAPLSAIQE 402
                ******************************************
```

fleN

```
PAO1            MKQMGSMHPVQVIAVTGGKGGVGKTNVSVNLALALADLGRRVMLLDADLGLANVDVLLGL 60
148             MKQMGSMHPVQVIAVTGGKGGVGKTNVSVNLALALADLGRRVMLLDADLGLANVDVLLGL 60
ID4365          ---MGSMHPVQVIAVTGGKGGVGKTNVSVNLALALADLGRRVMLLDADLGLANVDVLLGL 57
M10             MKQMGSMHPVQVIAVTGGKGGVGKTNVSVNLALALADLGRRVMLLDADLGLANVDVLLGL 60
IGB83           MKQMGSMHPVQVIAVTGGKGGVGKTNVSVNLALALADLGRRVMLLDADLGLANVDVLLGL 60
                   *********************************************************

PAO1            TPKRTLADVIEGRCELRDVLLLGPGGVRIVPAASGTQSMVHLSPMQHAGLIQAFSDISDN 120
148             TPKRTLADVIEGRCELRDVLLLGPGGVRIVPAASGTQSMVHLSPMQHAGLIQAFSDISDN 120
```

```
ID4365          TPKRTLADVIEGRCELRDVLLLGPGGVRIVPAASGTQSMVHLSPMQHAGLIQAFSDISDN 117
M10             TPKRTLADVIEGRCELRDVLLLGPGGVRIVPAASGTQSMVHLSPMQHAGLIQAFSDISDN 120
IGB83           TPKRTLADVIEGRCELRDVLLLGPGGVRIVPAASGTQSMVHLSPMQHAGLIQAFSDISDN 120
                ************************************************************

PAO1            LDVLVVDTAAGIGDSVVSFVRAAQEVLLVVCDEPTSITDAYALIKLLNRDHGMTRFRVLA 180
148             LDVLVVDTAAGIGDSVVSFVRAAQEVLLVVCDEPTSITDAYALIKLLNRDHGMTRFRVLA 180
ID4365          LDVLVVDTAAGIGDSVVSFVRAAQEVLLVVCDEPTSITDAYALIKLLNRDHGMTRFRVLA 177
M10             LDVLVVDTAAGIGDSVVSFVRAAQEVLLVVCDEPTSITDAYALIKLLNRDHGMTRFRVLA 180
IGB83           LDVLVVDTAAGIGDSVVSFVRAAQEVLLVVCDEPTSITDAYALIKLLNRDHGMTRFRVLA 180
                ************************************************************

PAO1            NMAHSPQEGRNLFAKLTKVTDRFLDVALQYVGVIPYDESVRKAVQKQRAVYEAFPRSKAS 240
148             NMAHSPQEGRNLFAKLTKVTDRFLDVALQYVGVIPYDESVRKAVQKQRAVYEAFPRSKAS 240
ID4365          NMAHSPQEGRNLFAKLTKVTDRFLDVALQYVGVIPYDESVRKAVQKQRAVYEAFPRSKAS 237
M10             NMAHSPQEGRNLFAKLTKVTDRFLDVALQYVGVIPYDESVRKAVQKQRAVYEAFPRSKAS 240
IGB83           NMAHSPQEGRNLFAKLTKVTDRFLDVALQYVGVIPYDESVRKAVQKQRAVYEAFPRSKAS 240
                ************************************************************

PAO1            LAFKAVAQKVDSWPLPANPRGHLEFFVERLVQHPATGSAV 280
148             LAFKAVAQKVDSWPLPANPRGHLEFFVERLVQHPATGSAV 280
ID4365          LAFKAVAQKVDSWPLPANPRGHLEFFVERLVQHPATGSAV 277
M10             LAFKAVAQKVDSWPLPANPRGHLEFFVERLVQHPATGSAV 280
IGB83           LAFKAVAQKVDSWPLPANPRGHLEFFVERLVQHPATGSAV 280
                ****************************************
```

motA

```
IGB83           MSKIIGIIVVFASVLGGFLLSGGKIGAIIQPFEVLIIGGAALGAFLQSNPGSTFMVVLKK 60
M10             MSKIIGIIVVFASVLGGFLLSGGKIGAIIQPFEVLIIGGAALGAFLQSNPGSTFMVVLKK 60
PAO1            MSKIIGIIVVFASVLGGFLLSGGKIGAIIQPFEVLIIGGAALGAFLQSNPGSTFMVVLKK 60
148             MSKIIGIIVVFASVLGGFLLSGGKIGAIIQPFEVLIIGGAALGAFLQANPGSTFMVVLKK 60
ID4365          MSKIIGIIVVFASVLGGFLLSGGKIGAIIQPFEVLIIGGAALGAFLQANPGSTFMVVLKK 60
                ***********************************************:************

IGB83           APKMFSNRFTQTYYLEVLGMLYEILNKSRREGMMAIEADIEDPAASPIFSKYPGVLKDER 120
M10             APKMFSNRFTQTYYLEVLGMLYEILNKSRREGMMAIEADIEDPAASPIFSKYPGVLKDER 120
PAO1            APKMFSNRFTQTYYLEVLGMLYEILNKSRREGMMAIEADIEDPAASPIFSKYPGVLKDER 120
148             APKMFSNRFTQTYYLEVLGMLYEILNKSRREGMMAIEADIEDPAASPIFSKYPGVLKDER 120
ID4365          APKMFSNRFTQTYYLEVLGMLYEILNKSRREGMMAIEADIEDPAASPIFSKYPGVLKDER 120
                ************************************************************

IGB83           MTAYVCDYLRIMSSGNMAPHELEGLFDMELSSLKEDLEHPSHAVTKVADALPGFGIVAAV 180
M10             MTAYVCDYLRIMSSGNMAPHELEGLFDMELSSLKEDLEHPSHAVTKVADALPGFGIVAAV 180
PAO1            MTAYVCDYLRIMSSGNMAPHELEGLFDMELSSLKEDLEHPSHAVTKVADALPGFGIVAAV 180
148             MTAYVCDYLRIMSSGNMAPHELEGLFDMELSSLKEDLEHPSHAVTKVADALPGFGIVAAV 180
ID4365          MTAYVCDYLRIMSSGNMAPHELEGLFDMELSSLKEDLEHPSHAVTKVADALPGFGIVAAV 180
                ************************************************************

IGB83           LGIVITMALLGEGSQAEIGHHVAAALVGTFLGILAAYGFVGPLAGALEHDAKEELNLFEA 240
M10             LGIVITMALLGEGSQAEIGHHVAAALVGTFLGILAAYGFVGPLAGALEHDAKEELNLFEA 240
PAO1            LGIVITMALLGEGSQAEIGHHVAAALVGTFLGILAAYGFVGPLAGALEHDAKEELNLFEA 240
148             LGIVITMALLGEGSQAEIGHHVAAALVGTFLGILAAYGFVGPLAVALEHDAKEELNMFEA 240
ID4365          LGIVITMALLGEGSQAEIGHHVAAALVGTFLGILAAYGFVGPLAVALEHDAKEELNMFEA 240
                ******************************************* ************:***

IGB83           IKACLVASASGMPPSLAVEFGRKVLLPAHRPTFAELEQAVRGR 283
M10             IKACLVASASGMPPSLAVEFGRKVLLPAHRPTFAELEQAVRGR 283
PAO1            IKACLVASASGMPPSLAVEFGRKVLLPAHRPTFAELEQAVRGR 283
148             IKACLVASASGMPPSLAVEFGRKVLLPAHRPTFAELEQAVRGR 283
ID4365          IKACLVASASGMPPSLAVEFGRKVLLPAHRPTFAELEQAVRGR 283
                *******************************************
```

motB

```
PAO1            MDNNQPIIVKRVKRYAAGHHGGSWKIAFADFATAMMAFFLVLWLLSSATPEQKKAISGYF 60
148             MDNNQPIIVKRVKRYAAGHHGGSWKIAFADFATAMMAFFLVLWLLSSATPEQKKAISGYF 60
IGB83           MDNNQPIIVKRVKRYAAGHHGGSWKIAFADFATAMMAFFLVLWLLSSATPEQKKAISGYF 60
M10             MDNNQPIIVKRVKRYAAGHHGGSWKIAFADFATAMMAFFLVLWLLSSATPEQKKAISGYF 60
ID4365          MDNNQPIIVKRVKRYAAGHHGGSWKIAFADFATAMMAFFLVLWLLSSATPEQKKAISGYF 60
                ************************************************************

PAO1            QDPIGFSESASPYVIDLGGTPTPAPDKTLNPQVQAQPDSNESRISPEQDHQVNADQAENL 120
148             QDPIGFSESASPYVIDLGGTPTPAPDKTLNPQVQAQPDSNESRISPEQDHQVNADQAENL 120
IGB83           QDPIGFSESASPYVIDLGGTPTPAPDKTLNPQVQAQPDSNESRISPEQDHQVNADQAENL 120
M10             QDPIGFSESASPYVIDLGGTPTPAPDKTLNPQVQAQPDSNESRISPEQDHQVNADQAENL 120
ID4365          QDPIGFSESASPYVIDLGGTPTPAPDKTLNPQVQAQPDSNESRISPEQDHQVNADQAENL 120
                ************************************************************

PAO1            AEQVERERLALLLQELQNKVDENPMLKDFKDQIHFEITRDGLRIQIVDAANRPMFDLGSA 180
148             AEQVERERLALLLQELQNKVDENPMLKDFKDQIHFEITRDGLRIQIVDAANRPMFDLGSA 180
IGB83           AEQVERERLALLLQELQNKVDENPMLKDFKDQIHFEITRDGLRIQIVDAANRPMFDLGSA 180
M10             AEQVERERLALLLQELQNKVDENPMLKDFKDQIHFEITRDGLRIQIVDAANRPMFDLGSA 180
```

```
ID4365          AEQVERERLALLLQELQNKVDENPMLKDFKDQIHFEITRDGLRIQIVDAANRPMFDLGSA 180
                ************************************************************

PAO1            RLQPYFEDILLAMAETIRQVPNKISISGHTDAKPYAGNGDFGNWELSANRANAARRALVA 240
148             RLQPYFEDILLAMAETIRQVPNKISISGHTDAKPYAGNGDFGNWELSANRANAARRALVA 240
IGB83           RLQPYFEDILLAMAETIRQVPNKISISGHTDAKPYAGNGDFGNWELSANRANAARRALVA 240
M10             RLQPYFEDILLAMAETIRQVPNKISISGHTDAKPYAGNGDFGNWELSANRANAARRALVA 240
ID4365          RLQPYFEDILLAMAETIRQVPNKISISGHTDAKPYAGNGDFGNWELSANRANAARRALVA 240
                ************************************************************

PAO1            GGYPEGQIAQVVGYASARLFDDKDPLNPVNRRIDIVVLTRKAQKAIEGETGAPEASAPAA 300
148             GGYPEGQIAQVVGYASARLFDDKDPLNPVNRRIDIVVLTRKAQKAIEGETGAPEASAPAA 300
IGB83           GGYPEGQIAQVVGYASARLFDDKDPLNPVNRRIDIVVLTRKAQKAIEGETGAPEASAPAA 300
M10             GGYPEGQIAQVVGYASARLFDDKDPLNPVNRRIDIVVLTRKAQKAIEGETGAPEASAPAA 300
ID4365          GGYPEGQIAQVVGYASARLFDDKDPLNPVNRRIDIVVLTRKAQKAIEGETGAPEASAPTA 300
                **********************************************************:*

PAO1            APGEQPKPAAEAPAAGAQPSLPADEVQKKLNLFDDGGSLKLEQIKGD 347
148             APGEQPKPAAEAPAAGAQPSLPADEVQKKLNLFDDGGSLKLEQIKGD 347
IGB83           APGEQPKPAAEAPAAGAQPSLPADEVQKKLNLFDDGGSLKLEQIKGD 347
M10             APGEQPKPAAEAPAAGAQPSLPADEVQKKLNLFDDGGSLKLEQIKGD 347
ID4365          APGEQPKPAAEAPAAGAQPSLPADEVQKKLNLFDDGGSLKLEQIKGD 347
                ***********************************************
```

motC

```
IGB83           MDVLSLVGIILAFVAIVGGNFLEGGHAGALLNGPAALIVIGGTLAAALLQTPVNVLKRGL 60
M10             MDVLSLVGIILAFVAIVGGNFLEGGHAGALLNGPAALIVIGGTLAAALLQTPVNVLKRGL 60
ID4365          MDVLSLVGIILAFVAIVGGNFLEGGHAGALLNGPAALIVIGGTLAAALLQTPVNVLKRGL 60
PAO1            MDVLSLVGIILAFVAIVGGNFLEGGHAGALLNGPAALIVIGGTLAAALLQTPVNVLKRGL 60
148             ----------------------------MPCSTAAALIVIGGTLAAALLQTPVNVLKRGL 32
                                               . .**************************

IGB83           GMLGWVFFPPREDLSGGIDRIVSWSMTARKEGLLGLESIADAEPDPYARKGLQLLVDGAE 120
M10             GMLGWVFFPPREDLSGGIDRIVSWSMTARKEGLLGLESIADAEPDPYARKGLQLLVDGAE 120
ID4365          GMLGWVFFPPREDLSGGIDRIVSWSMTARKEGLLGLESIADAEPDPYARKGLQLLVDGAE 120
PAO1            GMLGWVFFPPREDLSGGIDRIVSWSMTARKEGLLGLESIADAEPDPYARKGLQLLVDGAE 120
148             GMLGWVFFPPREDLSGGIDRIVSWSMTARKEGLLGLESIADAEPDPYARKGLQLLVDGAE 92
                ************************************************************

IGB83           PEVIRSILEVDLFTQESRDLQAAKVFESMGGYAPTIGIIGAVMGLIHVMGNLANPAQLGS 180
M10             PEVIRSILEVDLFTQESRDLQAAKVFESMGGYAPTIGIIGAVMGLIHVMGNLANPAQLGS 180
ID4365          PEVIRSILEVDLFTQESRDLQAAKVFESMGGYAPTIGIIGAVMGLIHVMGNLANPAQLGS 180
PAO1            PEVIRSILEVDLFTQESRDLQAAKVFESMGGYAPTIGIIGAVMGLIHVMGNLANPAQLGS 180
148             PEVIRSILEVDLFTQESRDLQAAKVFESMGGYAPTIGIIGAVMGLIHVMGNLANPAQLGS 152
                ************************************************************

IGB83           GIAVAFVATIYGVGFANLLLLPIGNKLKTLVLRQSRYREMLLEGLLSIAEGENPRSIELK 240
M10             GIAVAFVATIYGVGFANLLLLPIGNKLKTLVLRQSRYREMLLEGLLSIAEGENPRSIELK 240
ID4365          GIAVAFVATIYGVGFANLLLLPIGNKLKTLVLRQSRYREMLLEGLLSIAEGENPRSIELK 240
PAO1            GIAVAFVATIYGVGFANLLLLPIGNKLKTLVLRQSRYREMLLEGLLSIAEGENPRSIELK 240
148             GIAVAFVATIYGVGFANLLLLPIGNKLKTLVLRQSRYREMLLEGLLSIAEGENPRSIELK 212
                ************************************************************

IGB83           LQGFMD 246
M10             LQGFMD 246
ID4365          LQGFMD 246
PAO1            LQGFMD 246
148             LQGFMD 218
                ******
```

motD

```
IGB83           MQRRRRHQEEHENHERWLVSYADFITLLFAFFVVMYSISSINEGKYKILSETLTGVFNQP 60
M10             MQRRRRHQEEHENHERWLVSYADFITLLFAFFVVMYSISSINEGKYKILSETLTGVFNQP 60
ID4365          MQRRRRHQEEHENHERWLVSYADFITLLFAFFVVMYSISSINEGKYKILSETLTGVFNQP 60
PAO1            MQRRRRHQEEHENHERWLVSYADFITLLFAFFVVMYSISSINEGKYKILSETLTGVFNQP 60
148             MQRRRRHQEEHENHERWLVSYADFITLLFAFFVVMYSISSINEGKYKILSETLTGVFNQP 60
                ************************************************************

IGB83           DRSLKPIPIGDERPRTTEPERTSVEEQPSDNAASADSLERIADSVRDAFGDLIASDQLSV 120
M10             DRSLKPIPIGDERPRTTEPERTSVEEQPSDNAASADSLERIADSVRDAFGDLIASDQLSV 120
ID4365          DRSLKPIPIGDERPRTTEPERTSVEEQPSDNAASVDSLERIADSVRDAFGDLIASDQLSV 120
PAO1            DRSLKPIPIGDERPRTTEPERTSVEEQPSDNAASADSLERIADSVRDAFGDLIASDQLSV 120
148             DRSLKPIPIGDERPRTTEPERTSVEEQPSDNAASADSLERIADSVRGAFGDLIASDQLSV 120
                **********************************.***********.*************

IGB83           RGNELWIEITLNSSLLFPSGDALPNDAAFDIVEKVAKILAPYKNPIHVEGFTDDVPIHSP 180
M10             RGNELWIEITLNSSLLFPSGDALPNDAAFDIVEKVAKILAPYKNPIHVEGFTDDVPIHSP 180
ID4365          RGNELWIEITLNSSLLFPSGDALPNDAAFDIVEKVAKILAPYKNPIHVEGFTDDVPIHSP 180
PAO1            RGNELWIEITLNSSLLFPSGDALPNDAAFDIVEKVAKILAPYKNPIHVEGFTDDVPIHSP 180
148             RGNELWIEITLNSSLLFPSGDALPNDAAFDIVEKVAKILAPYKNPIHVEGFTDDVPIHSP 180
                ************************************************************
```

```
IGB83           RYPTNWELSAARAASIVRLLGNDGVEPSRMAAVGYGEFQPVADNASAEGRAKNRRVVLVI 240
M10             RYPTNWELSAARAASIVRLLGNDGVEPSRMAAVGYGEFQPVADNASAEGRAKNRRVVLVI 240
ID4365          RYPTNWELSAARAASIVRLLGNDGVEPSRMAAVGYGEFQPVADNASAEGRAKNRRVVLVI 240
PAO1            RYPTNWELSAARAASIVRLLGNDGVEPSRMAAVGYGEFQPVADNASAEGRAKNRRVVLVI 240
148             RYPTNWELSAARAASIVRLLGNDGVEPSRMAAVGYGEFQPVADNASAEGRAKNRRVVLVI 240
                ************************************************************

IGB83           SRNLEVRRSVSGVGSGKAQPDSALRHAGSAAGTQAADTGGTPAPAPATVNSPSPSP 296
M10             SRNLEVRRSVSGVGSGKAQPDSALRHAGSAAGTQAADTGGTPAPAPATVNSPSPSP 296
ID4365          SRNLEVRRSVSGVGSGKAQPDSALRHAGSAAGTQAADTGGTPAPAPATVNSPSPSP 296
PAO1            SRNLEVRRSVSGVGSGKAQPDSALRHAGSAAGTQAADTGGTPAPAPATVNSPSPSP 296
148             SRNLEVRRSVSGVGSGKAQPDSALRHAGSAAGTQAADTGGTPAPAPATVNSPSPSP 296
                ********************************************************
```

motY

```
148             MQPRLLLLPFLLSSLPALAVTFQTRLESVEWKVEGDQFECRLSQPVANFGVGEFVRRAGE 60
ID4365          MQPRLLLLPFLLSSLPALAVTFQTRLESVEWKVEGDQFECRLSQPVANFGVGEFVRRAGE 60
PAO1            MQPRLLLLPFLLSSLPALAVTFQTRLESVEWKVEGDQFECRLSQPVANFGVGEFVRRAGE 60
IGB83           MQPRLLLLPFLLSSLPALAVTFQTRLESVEWKVEGDQFECRLSQPVANFGVGEFVRRAGE 60
M10             MQPRLLLLPFLLSSLPALAVTFQTRLESVEWKVEGDQFECRLSQPVANFGVGEFVRRAGE 60
                ************************************************************

148             QATFRLKPEAQWLGRGSATLLAAAPPWRPGQGDINLGQVSIGSGEVPFNSNQQQAGRLLT 120
ID4365          QATFRLKPEAQWLGRGSATLLAAAPPWRPGQGDINLGQVSIGSGEVPFNSNQQQAGRLLT 120
PAO1            QATFRLKPEAQWLGRGSATLLAAAPPWRPGQGDINLGQVSIGSGEVPFNSSQQQAGRLLT 120
IGB83           QATFRLKPEAQWLGRGSATLLAAAPPWRPGQGDINLGQVSIGSGEVPFNSSQQQAGRLLT 120
M10             QATFRLKPEAQWLGRGSATLLAAAPPWRPGQGDINLGQVSIGSGEVPFNSSQQQAGRLLT 120
                **************************************************.*********

148             GLLEGRSPLVRHRTWQGDRLEVRLLPARFASVYSQYQACIAKLLPVNFDQVKLAQVGFPD 180
ID4365          GLLEGRSPLVRHRTWQGDRLEVRLLPARFASVYSQYQACIAKLLPVNFDQVKLAQVGFPD 180
PAO1            GLLEGRSPLVRHRTWQGDRLEVRLLPARFASVYSQYQACIAKLLPVNFDQVKLAQVGFPD 180
IGB83           GLLEGRSPLVRHRTWQGDRLEVRLLPARFASVYSQYQACIAKLLPVNFDQVKLAQVGFPD 180
M10             GLLEGRSPLVRHRTWQGDRLEVRLLPARFASVYSQYQACIAKLLPVNFDQVKLAQVGFPD 180
                ************************************************************

148             GGTALNDVARAKLDIILQLLKADPSINRIELDGHSDNSGNRLTNRDLSRRRALAVQEYLK 240
ID4365          GGTALNDVARAKLDIILQLLKADPSINRIELDGHSDNSGNRLTNRDLSRRRALAVQEYLK 240
PAO1            GGTALNDVARAKLDIILQLLKADPSINRIELDGHSDNSGNRLTNRDLSRRRALAVQEYLK 240
IGB83           GGTALNDVARAKLDIILQLLKADPSINRIELDGHSDNSGNRLTNRDLSRRRALAVQEYLK 240
M10             GGTALNDVARAKLDIILQLLKADPSINRIELDGHSDNSGNRLTNRDLSRRRALAVQEYLK 240
                ************************************************************

148             SNGVPESQINVRFYGERYPLVANNSAANRARNRRVTVHLSREAVVEPATEAPKAEDKPAP 300
ID4365          SNGVPESQINVRFYGERYPLVANNSAANRARNRRVTVHLSREAVVEPATEAPKAEDKPAP 300
PAO1            SNGVPESQINVRFYGERYPLVANNSAANRARNRRVTVHLSREAVVEPATEAPKAEDKPAP 300
IGB83           SNGVPESQINVRFYGERYPLVANNSAANRARNRRVTVHLSREAVVEPATEAPKAEDKPAP 300
M10             SNGVPESQINVRFYGERYPLVANNSAANRARNRRVTVHLSREAVVEPATEAPKAEDKPAP 300
                ************************************************************

148             PAAEPAAPKPP-AASLQGKPTV----- 321
ID4365          PAAEPAAPKPP-AASLQGKPTV----- 321
PAO1            PAAEPAAPKPP-AASLQGKPTV----- 321
IGB83           PAAEPAAPKPP-AASLQGKPTV----- 321
M10             PAAEPAAPRSLRPPRFRENPRFESRKR 327
                ********:.  .. :: :* .
```

fliH

```
PAO1            MVPHDKDKDNPSELIRGKDVAAFGLWSLPSFDEPRDEPAVAAPQVPAVAEPAPAPPAVEE 60
148             MVPHDKDKDNPSELIRGKDVAAFGLWSLPSFDEPRDEPAVAAPQVPAVAEPAPAPPAVEE 60
IGB83           MVPHDKDKDNPSELIRGKDVAAFGLWSLPSFDEPRDEPAVAAPQVPAVAEPAPAPPAVEE 60
M10             MVPHDKDKDNPSELIRGKDVAAFGLWSLPSFDEPRDEPAVAAPQVPAVAEPAPAPPAVEE 60
ID4365          MVPHDKDKDNPSELIRGKDVAAFGLWSLPSFDEPRDEPAVAAPQVPAVAEPAPAPAAVEE 60
                *******************************************************.****

PAO1            VELETVKPPTLEEIEAIRQDAYNEGFATGERDGFHAGQLKARQEAEEALKERLQSLERLM 120
148             VELETVKPPTLEEIEAIRQDAYNEGFATGERDGFHAGQLKARQEAEEALKERLQSLERLM 120
IGB83           VELETVKPPTLEEIEAIRQDAYNEGFATGERDGFHAGQLKARQEAEEALKERLQSLERLM 120
M10             VELETVKPPTLEEIEAIRQDAYNEGFATGERDGFHAGQLKARQEAEEALKERLQSLERLM 120
ID4365          VELETVKPPTLEEIEAIRQDAYNEGFATGERDGFHAGQLKARQEAEEALKERLQSLERLM 120
                ************************************************************

PAO1            TQLLEPIAEQDALIEQGMVNLVNHVARQVIQRELHMDSSHVRQVLREALKLLPMGAANIR 180
148             TQLLEPIAEQDALIEQGMVNLVNHVARQVIQRELHMDSSHVRQVLREALKLLPMGAANIR 180
IGB83           TQLLEPIAEQDALIEQGMVNLVNHVARQVIQRELHMDSSHVRQVLREALKLLPMGAANIR 180
M10             TQLLEPIAEQDALIEQGMVNLVNHVARQVIQRELHMDSSHVRQVLREALKLLPMGAANIR 180
ID4365          TQLLEPIAEQDTLIEQGMVNLVNHVARQVIQRELHMDSSHVRQVLREALKLLPMGAANIR 180
                ***********:************************************************

PAO1            IHVNPQDFERVKALRERHEESWRILEDDSLLPGGCRIETEHSRIDATIETRLAQAVKQLF 240
```

```
148             IHVNPQDFERVKALRERHEESWRILEDDSLLPGGCRIETEHSRIDATIETRLAQAVKQLF 240
IGB83           IHVNPQDFERVKALRERHEESWRILEDDSLLPGGCRIETEHSRIDATIETRLAQAVKQLF 240
M10             IHVNPQDFERVKALRERHEESWRILEDDSLLPGGCRIETEHSRIDATIETRLAQAVKQLF 240
ID4365          IHVNPQDFERVKALRERHEESWRILEDDSLLPGGCRIETEHSRIDATIETRLAQAVKQLF 240
                ************************************************************

PAO1            EQQREQATHPLASDIRIDLDAPGDVDAP 268
148             EQQREQATHPLASDIRIDLDAPGDVDAP 268
IGB83           EQQREQATHPLASDIRIDLDAPGDVDAP 268
M10             EQQREQATHPLASDIRIDLDAPGDVDAP 268
ID4365          EQQREQATHPLASDIRIDLDAPGDVDAP 268
                ****************************
```

fliS

```
IGB83           MYAMKAMKQYQQVSIEAQVSDANPHRLIQLLMQGGLERLAQARGAMEREQIPEKGILIGK 60
M10             MYAMKAMKQYQQVSIEAQVSDANPHRLIQLLMQGGLERLAQARGAMEREQIPEKGILIGK 60
PAO1            MYAMKAMKQYQQVSIEAQVSDANPHRLIQLLMQGGLERLAQARGAMEREQIPEKGILIGK 60
ID4365          MYAMKAMKQYQQVSIEAQVSDANPHRLIQLLMQGGLERLAQARGAMEREQIPEKGILIGK 60
148             ---MAAMRQYQNVSTQAQAIDASPHRLIQMLMEGGLTRMAQARGALERGEVALKGELIGK 57
                   * **:***:** :**. **.******:**:*** *:******:** ::. ** ****

IGB83           AIGIIGGLREALDSERGGELAGNLDRLYEYMIARLVEANTSNDTSLLDEVSALLLEVKSG 120
M10             AIGIIGGLREALDSERGGELAGNLDRLYEYMIARLVEANTSNDTSLLDEVSALLLEVKSG 120
PAO1            AIGIIGGLREALDSERGGELAGNLDRLYEYMIARLVEANTSNDTSLLDEVSALLLEVKSG 120
ID4365          AIGIIGGLREALDSERGGELAGNLDRLYEYMIARLVEANTSNDTNLLDEVSALLLEVKSG 120
148             AIAIVGGLREGLDFEAGGELAANLDRLYAYMSMRLTEANLKNDAGKLEEVSELLRNIKSG 117
                **.*:*****.** * *****.****** **  **.*** .**:. *:*** ** ::***

IGB83           WDGISH 126
M10             WDGISH 126
PAO1            WDGISH 126
ID4365          WDGISH 126
148             WDAIAP 123
                **.*:
```
